# Supplementary figures and images for: Acute SARS-CoV-2 infections harbor limited within-host diversity and transmit via tight transmission bottlenecks
Source: PLoS Pathog. 2021 Aug 23;17(8):e1009849. doi: 10.1371/journal.ppat.1009849 (PMC8412271; doi:10.1371/journal.ppat.1009849)

A

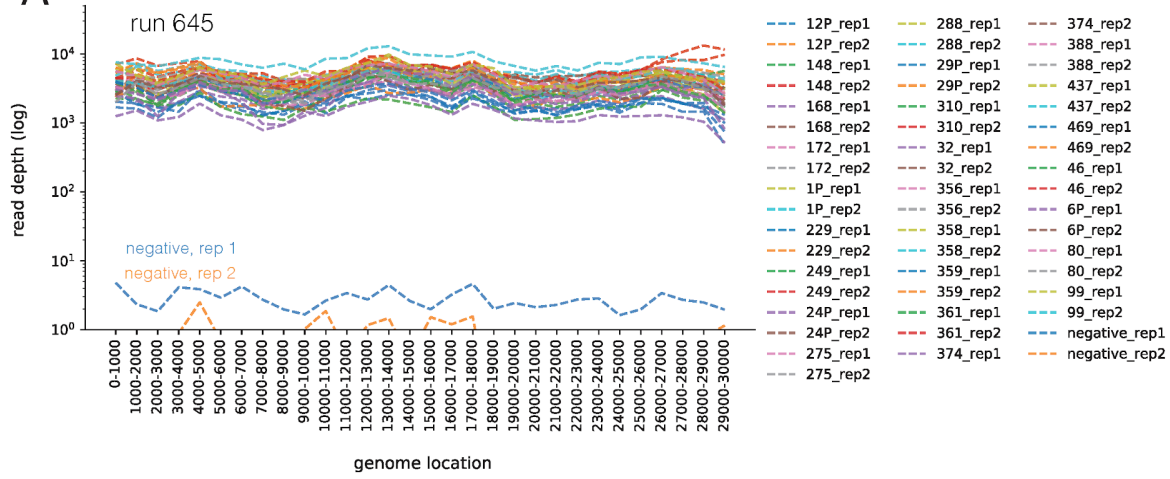

B

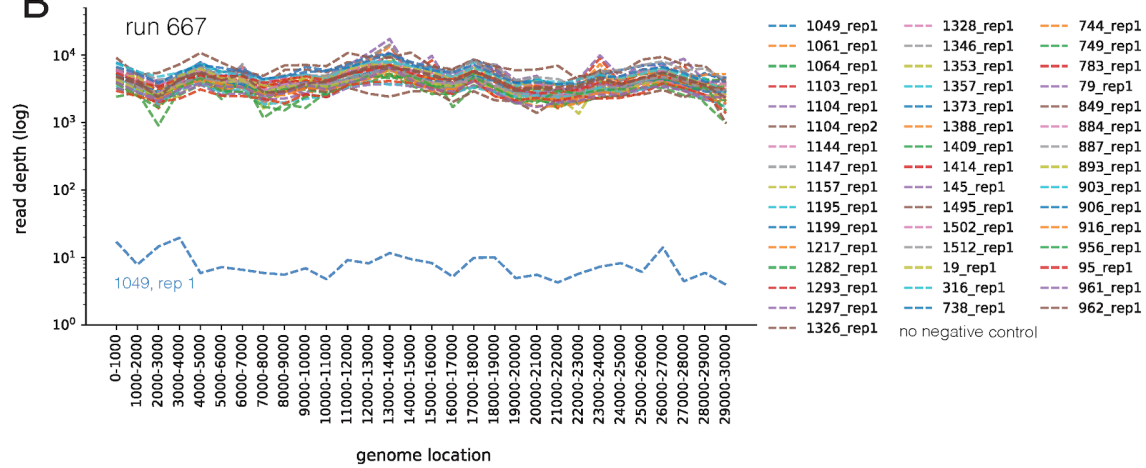

C

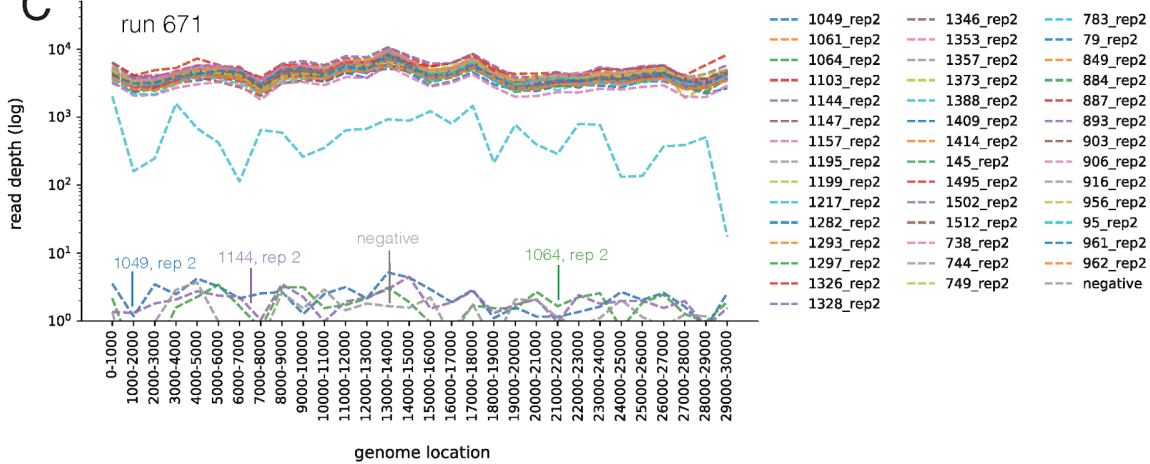

Supplement: S1 Fig — Read depth by genome location in 1,000-bp bins for MiSeq runs a. 627, b. 628, c. 643, and d. 644. Water controls and low-coverage samples are labeled. Samples included in each run are labeled according to the color to the right of each plot. (PDF) [file ppat.1009849.s001.pdf]

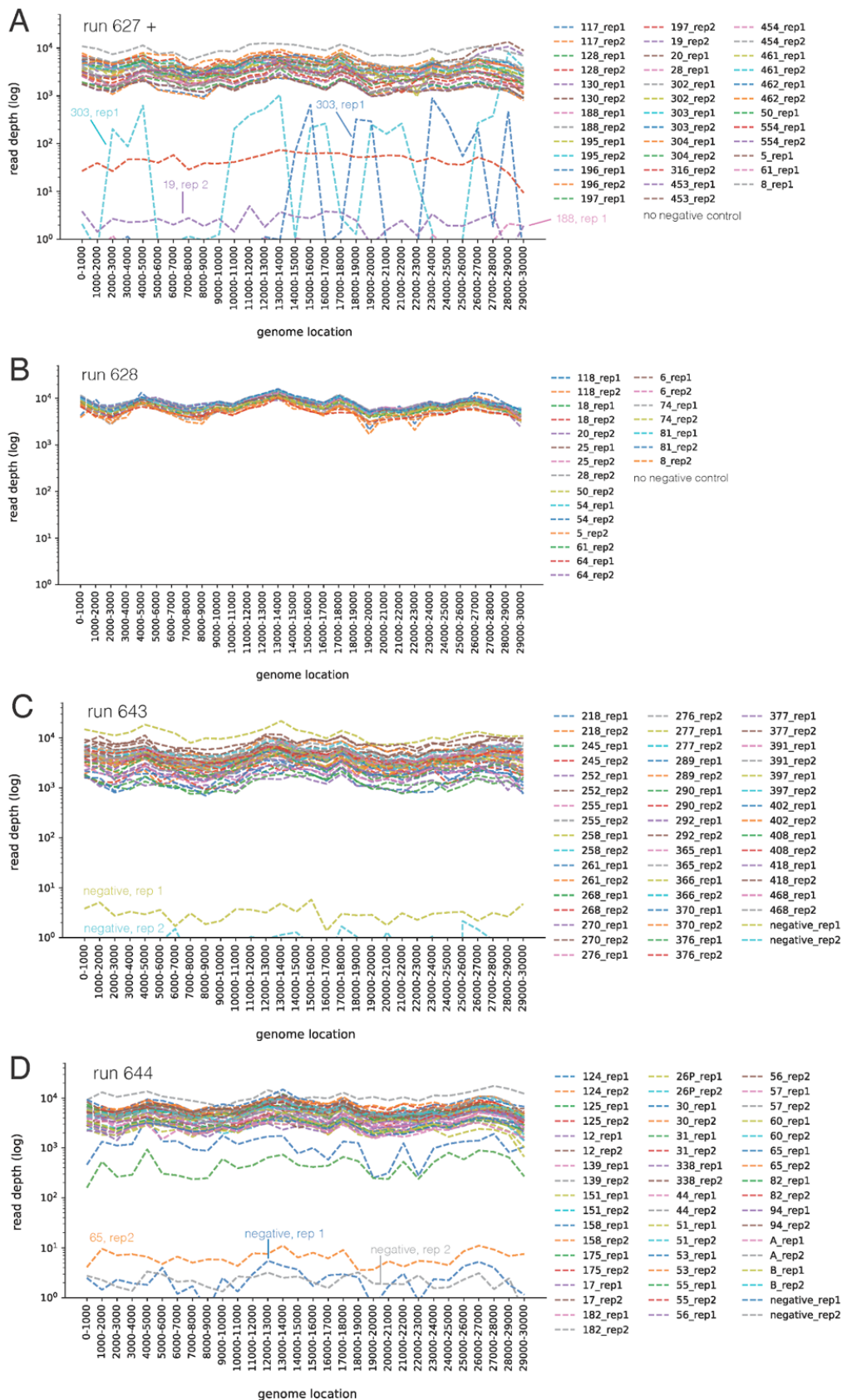

Supplement: S2 Fig — Read depth by genome location in 1,000-bp bins for MiSeq runs a. 645, b. 667, and c. 671. Water controls and low-coverage samples are labeled within each plot. Samples included in each run are labeled according to the color to the right of each plot. (PDF) [file ppat.1009849.s002.pdf]

**a.**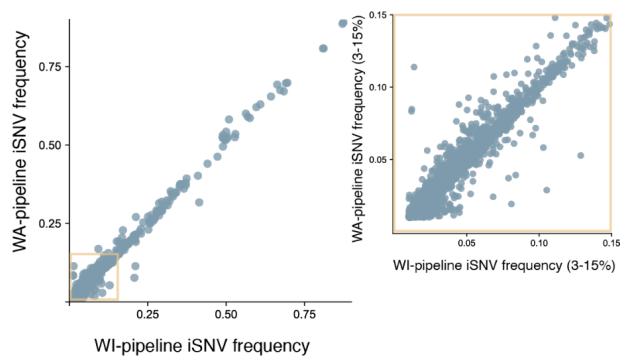**b.**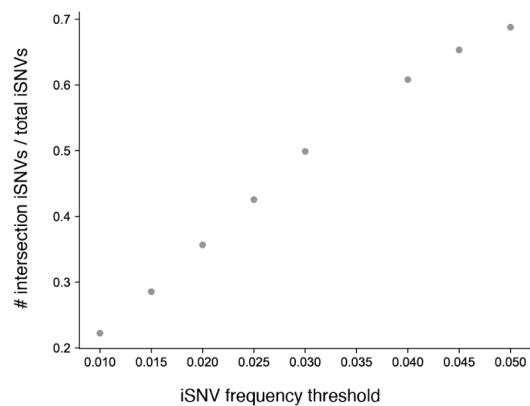**c.**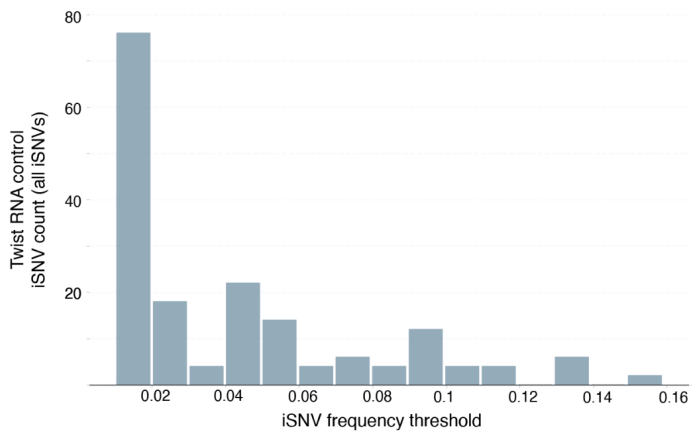**d.**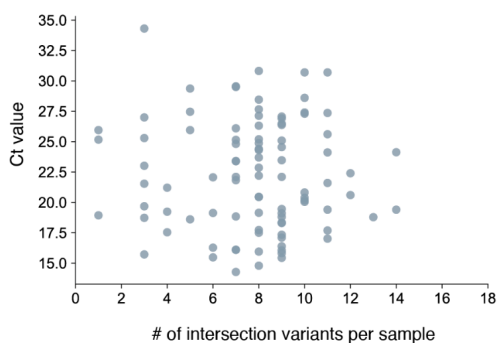**e.**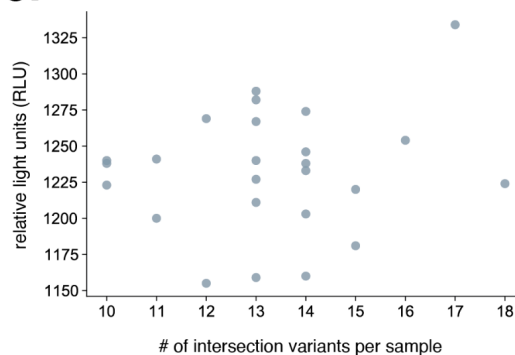

Supplement: S3 Fig — Subplot a. shows variant frequencies generated using the Wisconsin bioinformatic pipelines are shown on the x-axis and frequencies generated using the Washington bioinformatic pipeline are shown on the y-axis. The yellow box highlights low-frequency variants (3–15%), which is expanded out to the right. b. Proportion of intersection iSNVs relative to the total number of iSNVs increases as variant frequency threshold increases. c. The total number of iSNVs detected across both Twist RNA control replicates compared to the iSNV frequency threshold. 57/102 of iSNVs detected in these clonal samples occur <3% frequency. Note that the iSNVs reported in S1 Table are intersection iSNVs only. The identities of all iSNVs detected ≥1% frequency in the Twist RNA control can be found in the GitHub accompanything this manuscript. d. The number of intersection variants, both consensus and iSNVs, is compared to the Ct value for all samples where a Ct value was available. Out of 133 total samples, Ct values were available for 94. e. The number of intersection variants, both consensus and iSNVs, is compared to the RLU (relative light unit) value for all samples where a RLU value was available. (PDF) [file ppat.1009849.s003.pdf]

**a.**

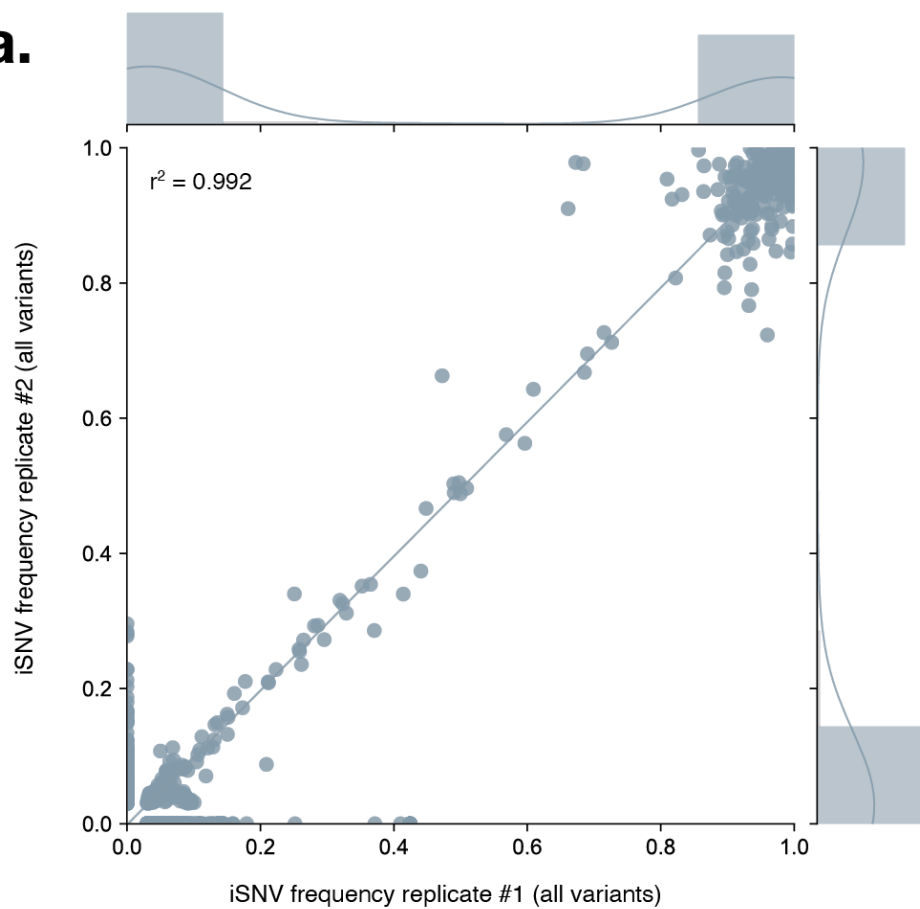

**b.**

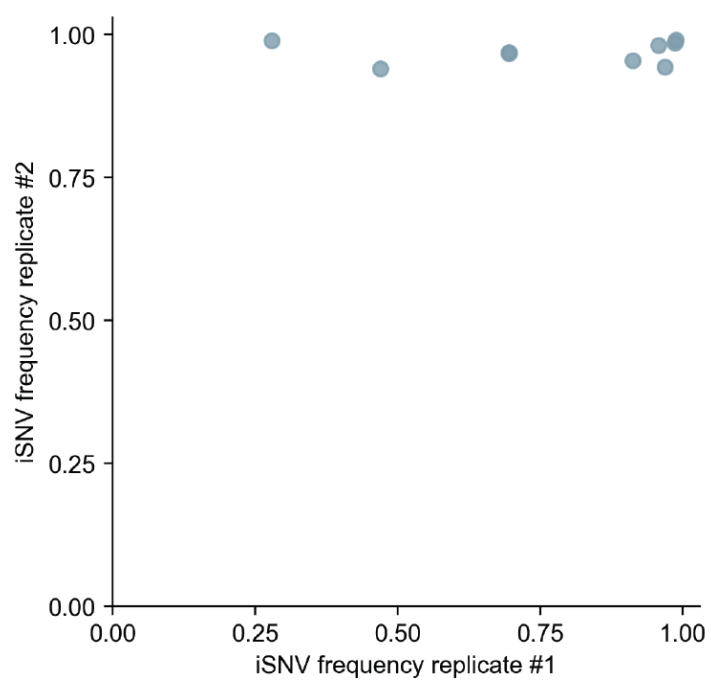

Supplement: S4 Fig — a. Variant frequencies in replicate 1 are shown on the x-axis and frequencies in replicate 2 are shown on y-axis. This plot includes all variants found in both replicates and not just the intersection variants as shown Fig 1A and 1B. Example of one sample with very poor overlap between technical replicates; this sample (sample 1104) was excluded from the experimental dataset. (PDF) [file ppat.1009849.s004.pdf]

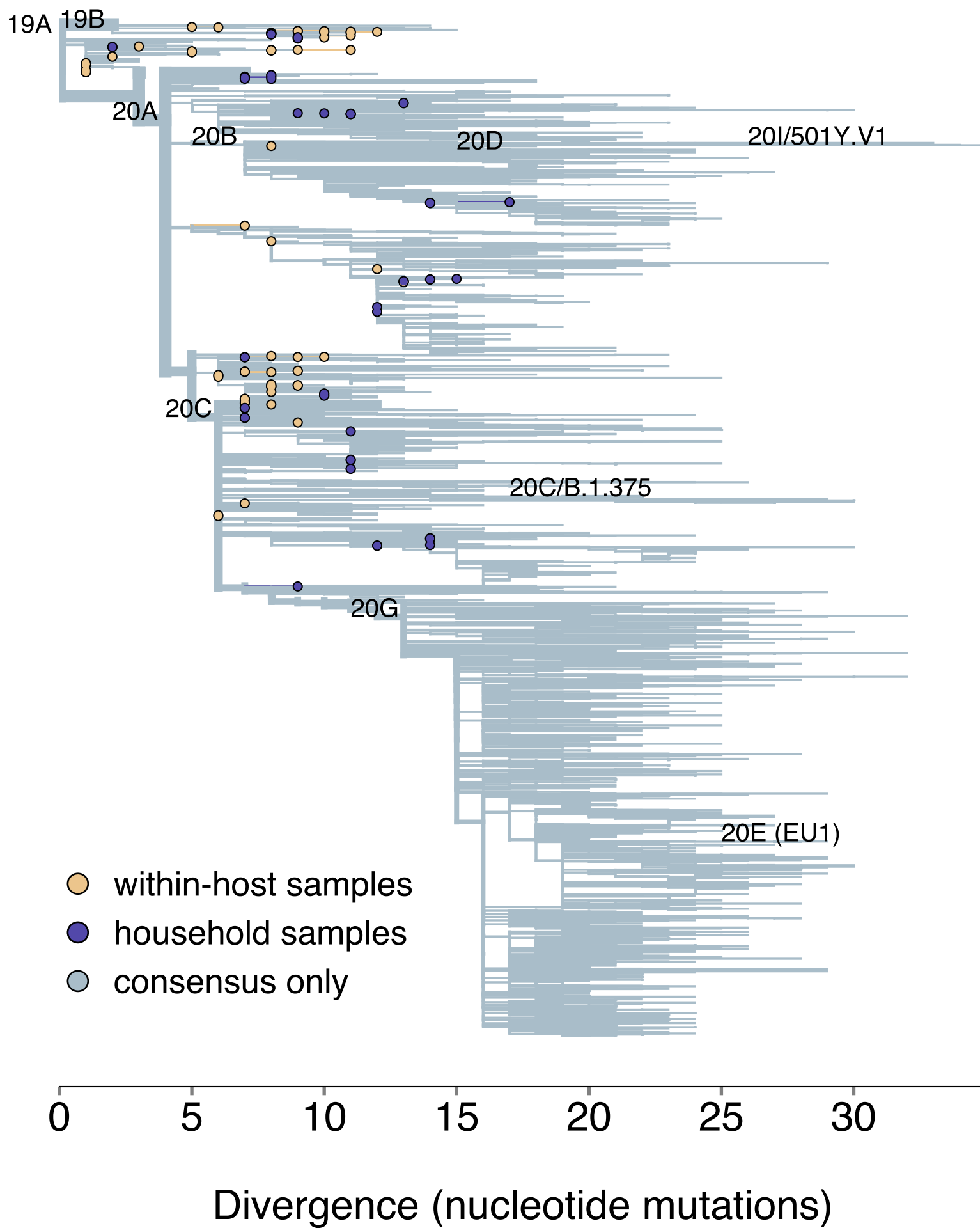

Supplement: S6 Fig — A full-genome phylogenetic tree built showing 6306 Wisconsin consensus sequences with the Nextstrain pipeline is shown. The x-axis represents divergence expressed as the number of nucleotide mutations. Nextstrain clade labels are shown on the corresponding branch. Yellow tips represent Wisconsin samples that were Illumina sequenced in duplicate and analyzed in this manuscript. Purple tips represent samples from households. (PDF) [file ppat.1009849.s006.pdf]

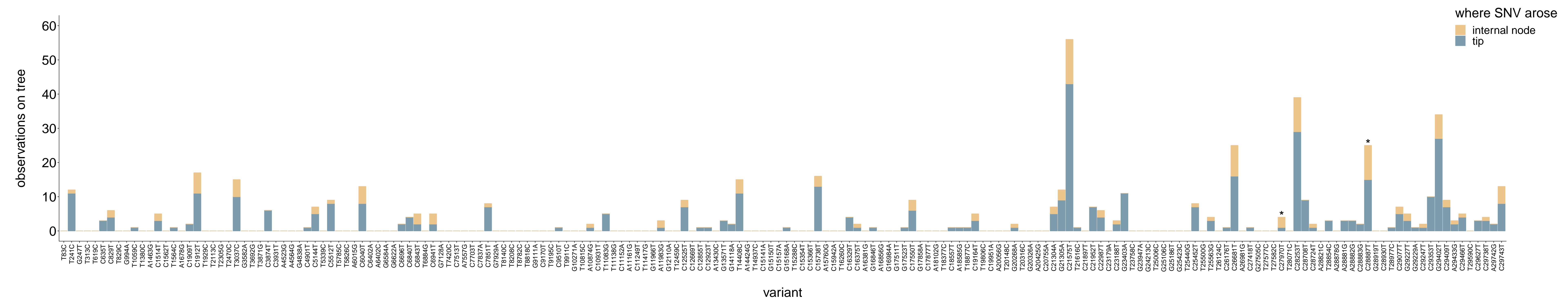

Supplement: S7 Fig — We queried every iSNV that was detected within-host (in at least 1 sample) in the global SARS-CoV-2 phylogenetic tree and quantified the number of times that iSNV was detected on an internal node (yellow bar heights) or on a terminal node/tip (blue bar heights). 42% of all iSNVs detected within-host were found on the tree. Most iSNVs that were detected on the tree were rare, and occurred predominantly on terminal nodes. Please note you will likely need to zoom into this figure to clearly read the labels along the x-axis. (PDF) [file ppat.1009849.s007.pdf]

Pr(x mutations) within  
one serial interval

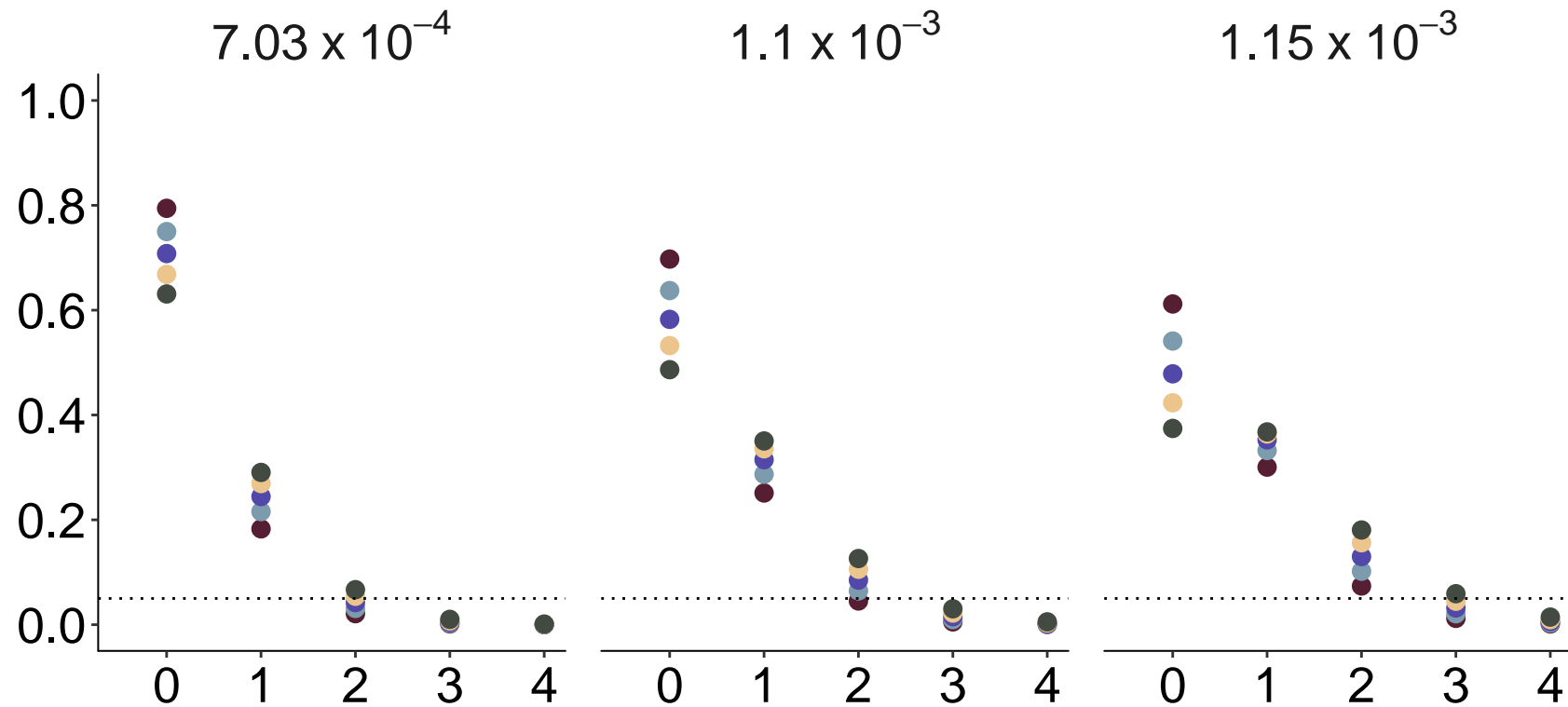

serial interval  
(days)

- 4
- 5
- 6
- 7
- 8

number of mutations

Supplement: S8 Fig — To define whether infections sampled from the same household might be true transmission pairs, we explored the expected number of consensus mutations that should differ between genomes separated by one serial interval. We modeled the probability that 2 consensus genomes will share x mutations as Poisson distributed with lambda equal to the number of mutations expected to accumulate in the SARS-CoV-2 genome over a single serial interval, given a known substitution rate. He et al. estimate a serial interval for SARS-CoV-2 of of 5.8 days, with a 95% confidence interval between 4.8–6.8 days [35]. We therefore evaluated serial intervals of 4, 5, 6, 7, and 8 days. For the substitution rate, we use estimates from Duchene et al [1], who estimate a mean substitution rate of 1.10 x 10^−3^ substitutions per site per year, with a 95% credible interval of 7.03 x 10^−4^ and 1.15 x 10^−3^. We evaluated the probabilities that two consensus genomes differ by 0, 1, 2, 3, and 4 mutations given serial intervals ranging from 4–8, and clock rates at the mean, and upper and lower bounds of the 95% credible interval. For each calculated probability, the serial interval is represented by color and the substitution rate is shown above each plot. The dotted line represents a probability of 0.05. Given these combinations of values, the vast majority of consensus genomes are expected to differ by 0–2 mutations. (PDF) [file ppat.1009849.s008.pdf]

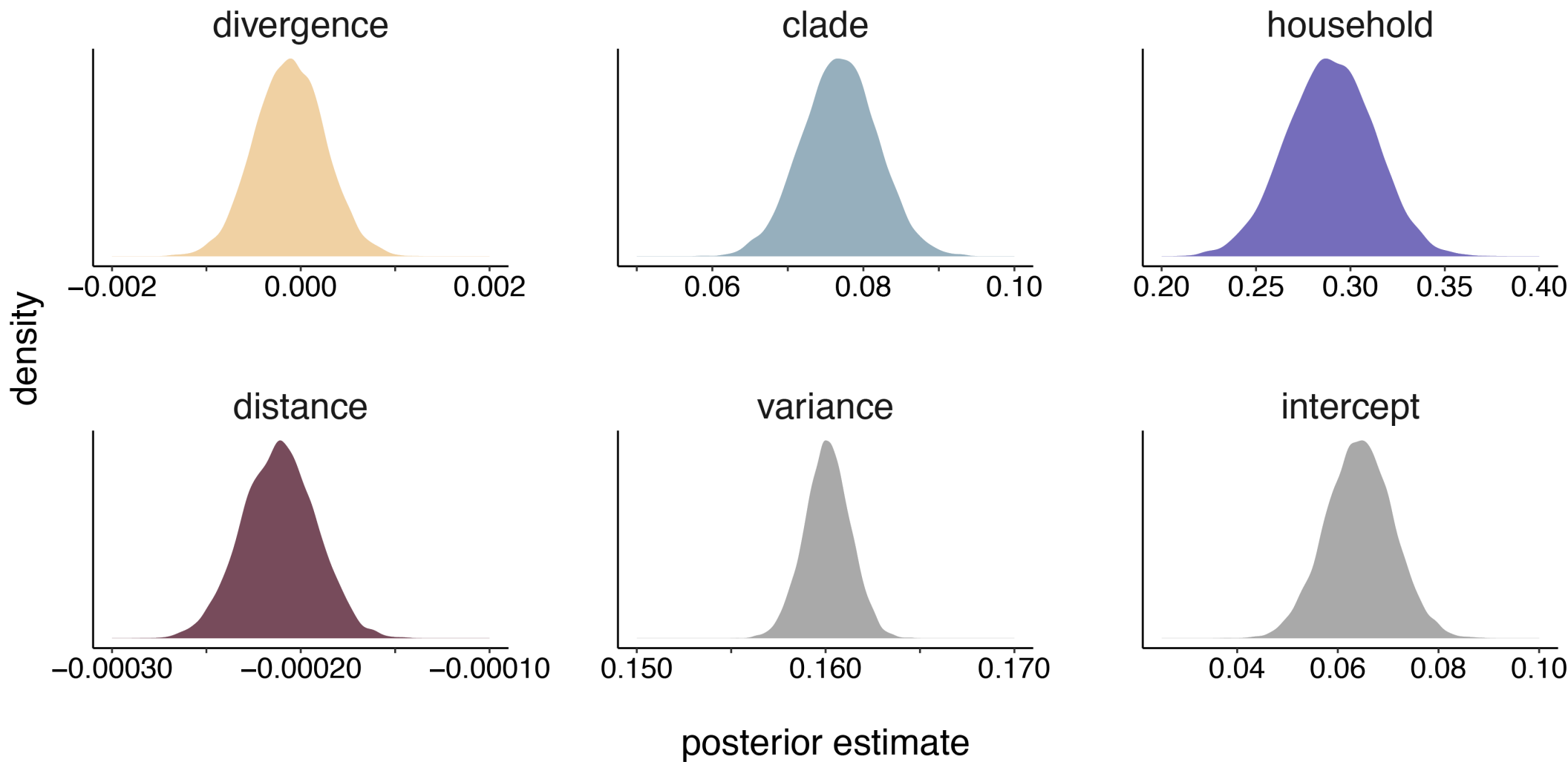

Supplement: S9 Fig — For each regression coefficient evaluated in the combined regression model, the full posterior distribution is shown as a density plot. The posterior distribution of the estimated variance and intercept are also shown. (PDF) [file ppat.1009849.s009.pdf]

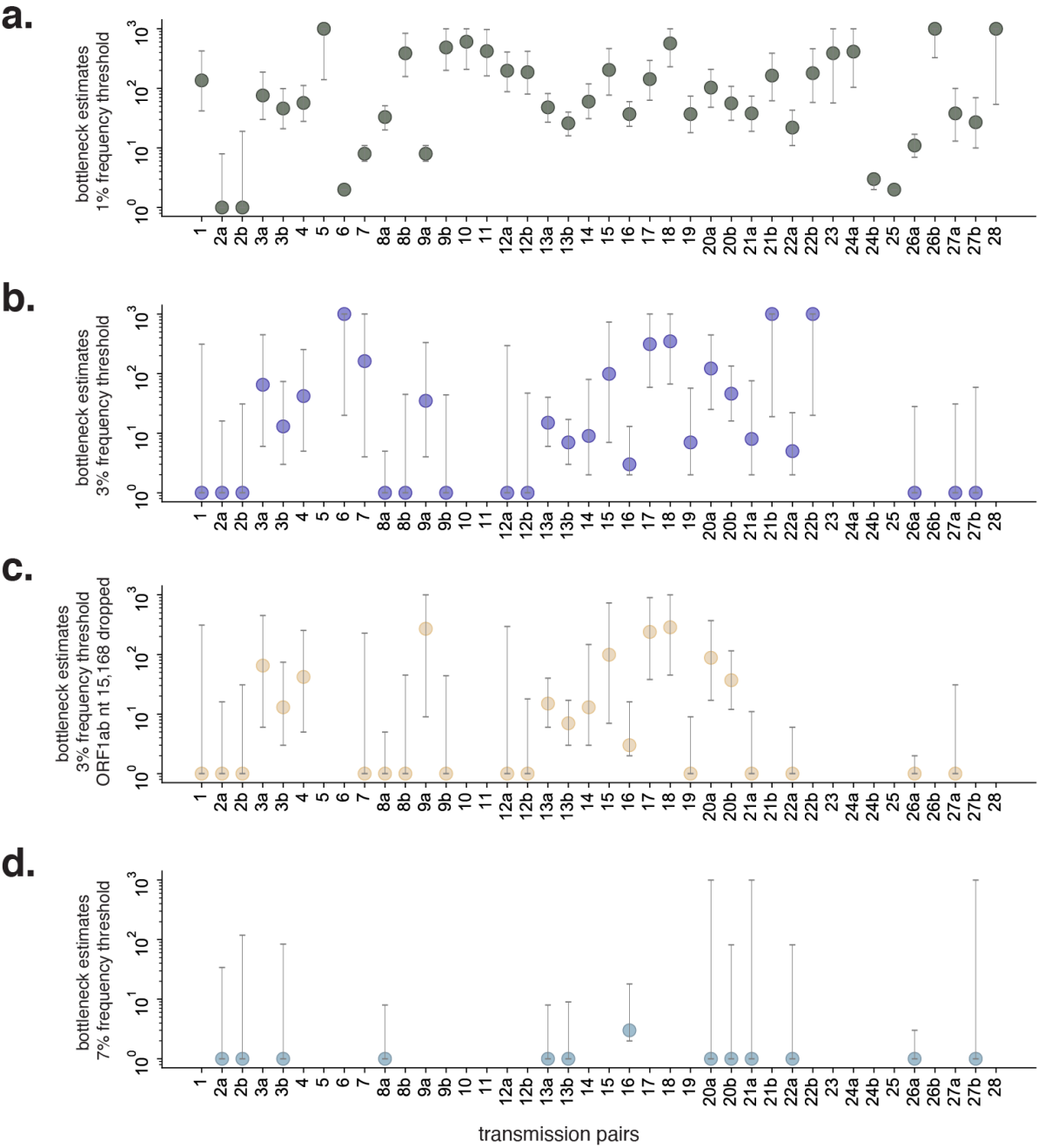

Supplement: S10 Fig — Maximum likelihood estimates for mean transmission bottleneck size in individual donor-recipient pairs using a. 1% frequency threshold, b. 3% frequency threshold, c. excluding site 15,168 as a possible homoplasy with a 3% frequency threshold, and d. 7% frequency threshold. Data are not shown for donor-recipient pairs where no bottleneck estimate could be generated due to lack of variant data. Bidirectional comparisons are indicated with an “a” and “b” following the pair number. (PDF) [file ppat.1009849.s010.pdf]

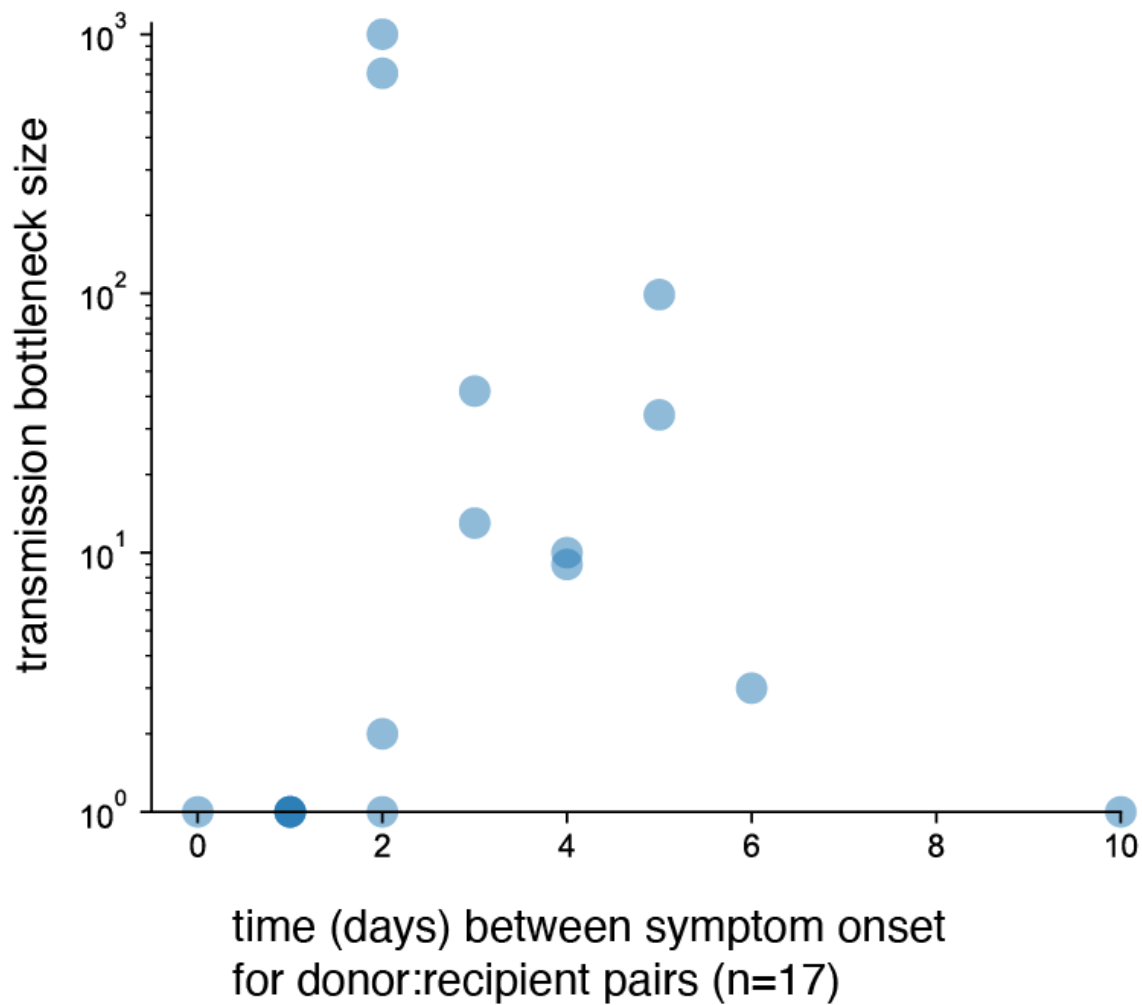

Supplement: S11 Fig — We plotted transmission bottleneck size on the y-axis against time (days) between symptom onset in 17 donor-recipient pairs on the x-axis for which we had symptom metadata. (PDF) [file ppat.1009849.s011.pdf]

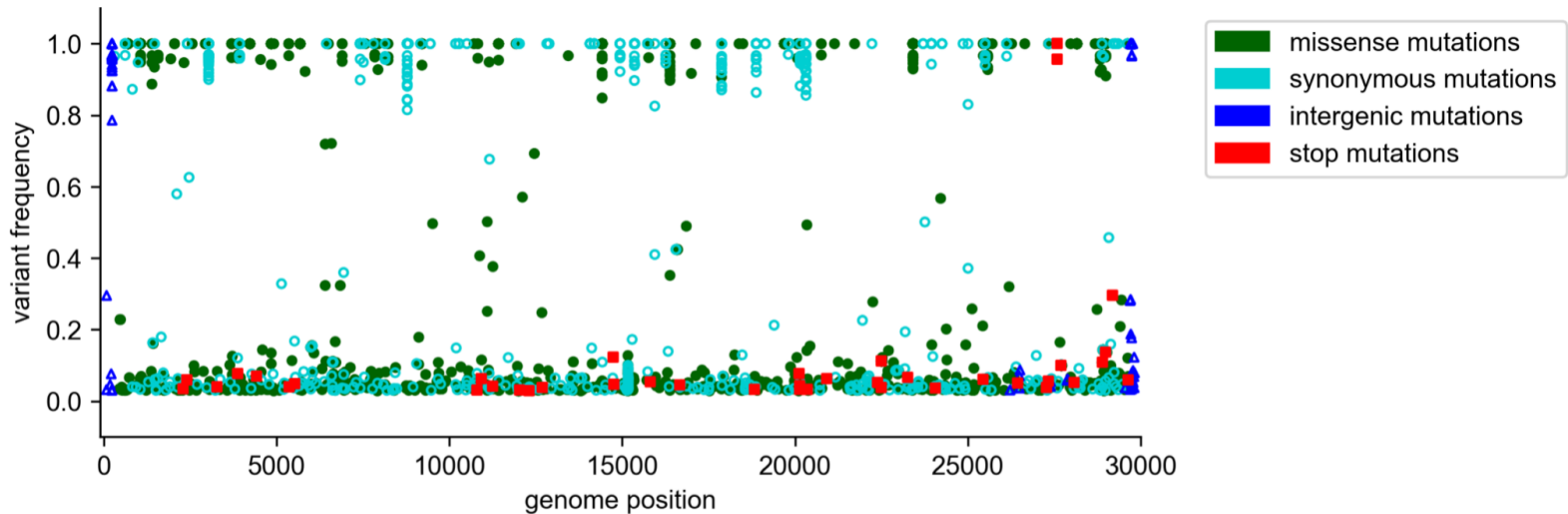

Supplement: S12 Fig — The frequency of all SNVs are plotted across the SARS-CoV-2 genome. Each variant is colored by mutation type. (PDF) [file ppat.1009849.s012.pdf]
