# Supplementary material for: Acute SARS-CoV-2 infections harbor limited within-host diversity and transmit via tight transmission bottlenecks
Source: PLoS Pathog. 2021 Aug 23;17(8):e1009849. doi: 10.1371/journal.ppat.1009849 (PMC8412271; doi:10.1371/journal.ppat.1009849)
Supplement: S5 Fig — iSNVs detected in at least 2 samples are shown on the x-axis and are plotted against the number of times they are detected in our dataset. Each iSNV bar is colored according to the number of times it was detected within each sequencing batch. (PDF) [file ppat.1009849.s005.pdf]

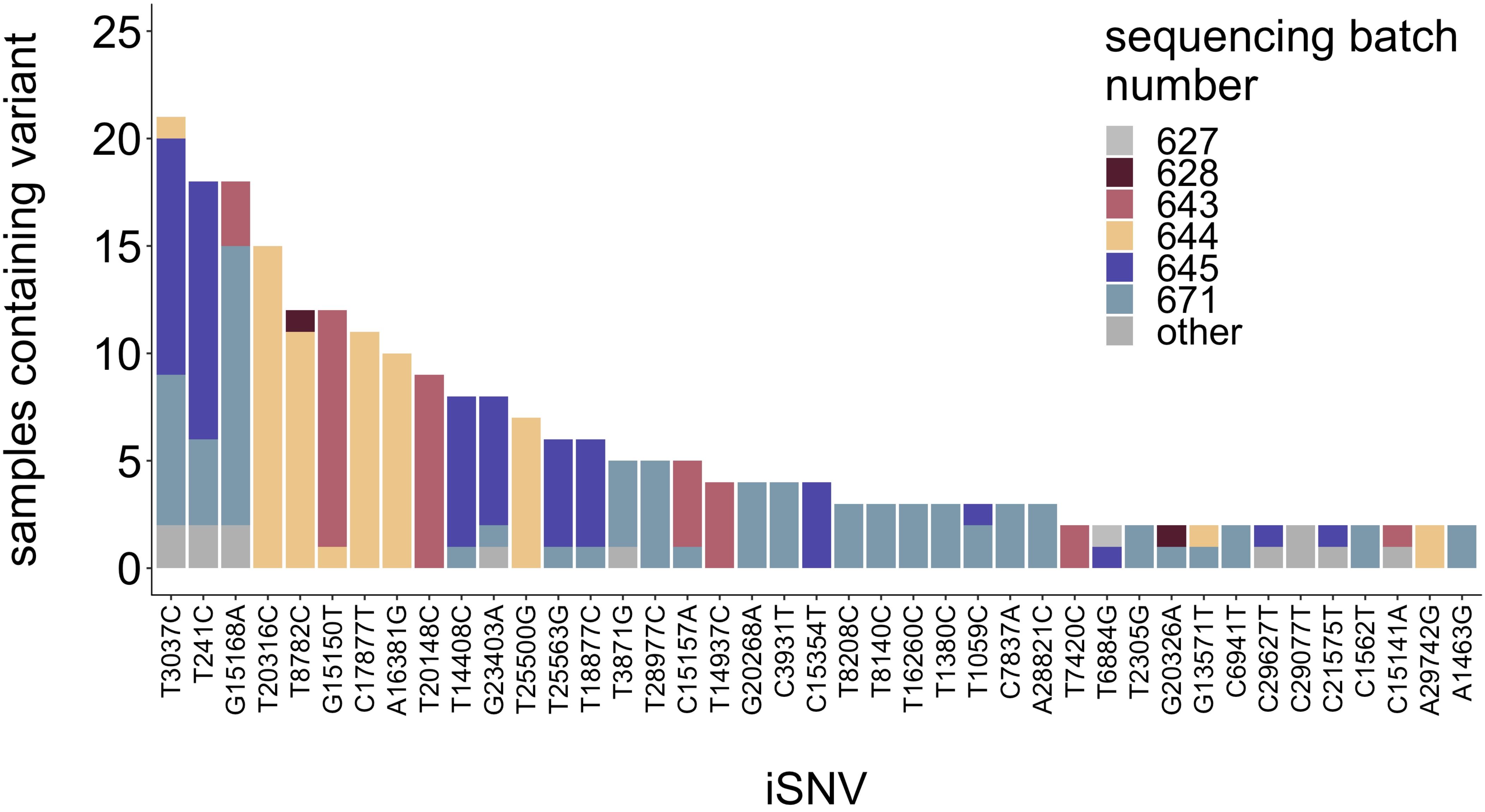

sequencing batch number
627
628
643
644
645
671
other
samples containing variant
0
5
10
15
20
25
T3037C
T241C
G15168A
T20316C
T8782C
G15150T
C17877T
A16381G
T20148C
T14408C
G23403A
T25500G
T25563G
T18877C
T3871G
T28977C
C15157A
T14937C
G20268A
C3931T
C15354T
T8208C
T8140C
T16260C
T1380C
T1059C
C7837A
A28821C
T7420C
T6884G
T23305G
G20326A
G13571T
C6941T
C29627T
C29077T
C21575T
C1562T
C15141A
A29742G
A1463G
iSNV
