# Supplementary material for: Acute SARS-CoV-2 infections harbor limited within-host diversity and transmit via tight transmission bottlenecks
Source: PLoS Pathog. 2021 Aug 23;17(8):e1009849. doi: 10.1371/journal.ppat.1009849 (PMC8412271; doi:10.1371/journal.ppat.1009849)
Supplement: S1 Table — All iSNVs called in the synthetic RNA control from Twist Biosciences are shown. (DOCX) [file ppat.1009849.s013.docx]

| **Mutation** | | | | | | |  | | |
| --- | --- | --- | --- | --- | --- | --- | --- | --- | --- |
| **Gene** | **Reference amino acid** | **Amino acid position** | **Variant amino acid** | **Reference nucleotide** | **Nucleotide position** | **Variant nucleotide** | **rep1 percent** | **rep2 percent** | **Average percent** |
| orf1ab | Ser | 1029 | Cys | A | 3350 | T | 0.0406 | 0.0441 | 0.04235 |
| orf1ab | Trp | 2135 | *Stop | G | 6669 | A | 0.0304 | 0.0347 | 0.03255 |
| orf1ab | Gly | 2863 | Val | G | 8853 | T | 0.0103 | 0.011 | 0.01065 |
| orf1ab | Thr | 2967 | Ser | A | 9164 | T | 0.0125 | 0.0109 | 0.0117 |
| M | Leu | 90 | *Stop | T | 26791 | A | 0.1329 | 0.1368 | 0.13485 |
| M | Met | 90 | Val | A | 26793 | G | 0.1313 | 0.1354 | 0.13335 |
| M | Trp | 92 | Arg | T | 26796 | A | 0.131 | 0.1352 | 0.1331 |
