# Supplementary material for: Acute SARS-CoV-2 infections harbor limited within-host diversity and transmit via tight transmission bottlenecks
Source: PLoS Pathog. 2021 Aug 23;17(8):e1009849. doi: 10.1371/journal.ppat.1009849 (PMC8412271; doi:10.1371/journal.ppat.1009849)
Supplement: S2 Table — This table includes strain name, tube/filename, state of collection, county of collection, collection date, GISAID accession number, Genbank accession number, as well as Ct values and RLU values where available for each sample included in this study. (DOCX) [file ppat.1009849.s014.docx]

|  |  |  |  |  |  |  | **Nanopore data** | **Illumina data** |  |  |  |
| --- | --- | --- | --- | --- | --- | --- | --- | --- | --- | --- | --- |
| **Strain** | **Tube** | **State** | **County** | **Collection Date** | **GISAID Accession** | **Genbank Accession** | **BioProject** | **BioProject** | **N1 Ct**  **value** | **N2 Ct**  **value** | **RLU** |
| USA/WI-UW-06/2020 | A | Wisconsin | Dane County | 2020-3-21 | EPI_ISL_417200 | MT772088 | PRJNA614504 | PRJNA718341 | 26.53 | 27.29 | - |
| USA/WI-UW-07/2020 | B | Wisconsin | Dane County | 2020-3-21 | EPI_ISL_417201 | MT772089 | PRJNA614504 | PRJNA718341 | 16.28 | 16.49 | - |
| USA/WI-UW-11/2020 | 1P | Wisconsin | Dane County | 2020-3-15 | EPI_ISL_417505 | MT706133 | PRJNA614504 | PRJNA718341 | - | - | - |
| USA/WI-UW-29/2020 | 5 | Wisconsin | Dane County | 2020-3-24 | EPI_ISL_421287 | MT706150 | PRJNA614504 | PRJNA718341 | 16.14 | 16.05 | - |
| USA/WI-UW-30/2020 | 6 | Wisconsin | Columbia County | 2020-3-26 | EPI_ISL_421288 | MT706151 | PRJNA614504 | PRJNA718341 | 24.76 | 25.41 | - |
| USA/WI-UW-14/2020 | 6P | Wisconsin | Dane County | 2020-3-16 | EPI_ISL_417513 | MT706136 | PRJNA614504 | PRJNA718341 | - | - | - |
| USA/WI-UW-32/2020 | 8 | Wisconsin | Dane County | 2020-3-24 | EPI_ISL_421290 | MT706153 | PRJNA614504 | PRJNA718341 | 24.27 | 24.83 | - |
| USA/WI-UW-34/2020 | 12 | Wisconsin | Dane County | 2020-3-26 | EPI_ISL_421292 | MT706155 | PRJNA614504 | PRJNA718341 | 27.81 | 29.4 | - |
| USA/WI-UW-17/2020 | 12P | Wisconsin | Dane County | 2020-3-13 | EPI_ISL_417517 | MT706139 | PRJNA614504 | PRJNA718341 | - | - | - |
| USA/WI-UW-38/2020 | 17 | Wisconsin | Dane County | 2020-3-25 | EPI_ISL_421296 | MT706159 | PRJNA614504 | PRJNA718341 | 27.98 | 29.23 | - |
| USA/WI-UW-40/2020 | 19 | Wisconsin | Dane County | 2020-3-24 | EPI_ISL_421298 | MT706161 | PRJNA614504 | PRJNA718341 | 23.52 | 24.46 | - |
| USA/WI-UW-39/2020 | 18 | Wisconsin | Dane County | 2020-3-22 | EPI_ISL_421297 | MT706160 | PRJNA614504 | PRJNA718341 | 18.2 | 20.06 | - |
| USA/WI-UW-41/2020 | 20 | Wisconsin | Dane County | 2020-3-25 | EPI_ISL_421299 | MT706162 | PRJNA614504 | PRJNA718341 | 24.32 | 25.31 | - |
| USA/WI-UW-21/2020 | 24P | Wisconsin | Dane County | 2020-3-16 | EPI_ISL_417508 | MT706142 | PRJNA614504 | PRJNA718341 | - | - | - |
| USA/WI-UW-45/2020 | 25 | Wisconsin | Dane County | 2020-3-22 | EPI_ISL_421303 | MT706166 | PRJNA614504 | PRJNA718341 | 20.22 | 20.7 | - |
| USA/WI-UW-22/2020 | 26P | Wisconsin | Dane County | 2020-3-13 | EPI_ISL_417514 | MT706143 | PRJNA614504 | PRJNA718341 | - | - | - |
| USA/WI-UW-48/2020 | 28 | Wisconsin | Dane County | 2020-3-25 | EPI_ISL_421306 | MT706169 | PRJNA614504 | PRJNA718341 | 23.02 | 23.79 | - |
| USA/WI-UW-24/2020 | 29P | Wisconsin | Dane County | 2020-3-15 | EPI_ISL_417512 | MT706145 | PRJNA614504 | PRJNA718341 | - | - | - |
| USA/WI-UW-50/2020 | 30 | Wisconsin | Green County | 2020-3-25 | EPI_ISL_421308 | MT706171 | PRJNA614504 | PRJNA718341 | 19.13 | 19.78 | - |
| USA/WI-UW-51/2020 | 31 | Wisconsin | Dane County | 2020-3-20 | EPI_ISL_421309 | MT706172 | PRJNA614504 | PRJNA718341 | 17.11 | 17.3 | - |

| USA/WI-UW-52/2020 | 32 | Wisconsin | Dane County | 2020-3-18 | EPI_ISL_421310 | MT706173 | PRJNA614504 | PRJNA718341 | 15.98 | 16.57 | - |
| --- | --- | --- | --- | --- | --- | --- | --- | --- | --- | --- | --- |
| USA/WI-UW-61/2020 | 44 | Wisconsin | Dane County | 2020-3-23 | EPI_ISL_421319 | MT706182 | PRJNA614504 | PRJNA718341 | 23.28 | 24.11 | - |
| USA/WI-UW-63/2020 | 46 | Wisconsin | Dane County | 2020-3-24 | EPI_ISL_421321 | MT706184 | PRJNA614504 | PRJNA718341 | 24.54 | 25.21 | - |
| USA/WI-UW-65/2020 | 50 | Wisconsin | Dane County | 2020-3-22 | EPI_ISL_421323 | MT706186 | PRJNA614504 | PRJNA718341 | 15.54 | 15.33 | - |
| USA/WI-UW-66/2020 | 51 | Wisconsin | Dane County | 2020-3-24 | EPI_ISL_421324 | MT706187 | PRJNA614504 | PRJNA718341 | 26.52 | 27.61 | - |
| USA/WI-UW-67/2020 | 53 | Wisconsin | Dane County | 2020-3-25 | EPI_ISL_421325 | MT706188 | PRJNA614504 | PRJNA718341 | 25.62 | 27.01 | - |
| USA/WI-UW-68/2020 | 54 | Wisconsin | Dane County | 2020-3-24 | EPI_ISL_421326 | MT706189 | PRJNA614504 | PRJNA718341 | 15.96 | 16.13 | - |
| USA/WI-UW-69/2020 | 55 | Wisconsin | Dane County | 2020-3-19 | EPI_ISL_421327 | MT706190 | PRJNA614504 | PRJNA718341 | 15.83 | 16.07 | - |
| USA/WI-UW-70/2020 | 56 | Wisconsin | Dane County | 2020-3-19 | EPI_ISL_421328 | MT706191 | PRJNA614504 | PRJNA718341 | 20.12 | 20.77 | - |
| USA/WI-UW-71/2020 | 57 | Wisconsin | Dane County | 2020-3-24 | EPI_ISL_421329 | MT706192 | PRJNA614504 | PRJNA718341 | 18.93 | 18.64 | - |
| USA/WI-UW-73/2020 | 60 | Wisconsin | Dane County | 2020-3-24 | EPI_ISL_421331 | MT706194 | PRJNA614504 | PRJNA718341 | 23.69 | 24.93 | - |
| USA/WI-UW-74/2020 | 61 | Wisconsin | Dane County | 2020-3-20 | EPI_ISL_421332 | MT706195 | PRJNA614504 | PRJNA718341 | 14.19 | 14.36 | - |
| USA/WI-UW-76/2020 | 64 | Wisconsin | Dane County | 2020-3-22 | EPI_ISL_421334 | MT706197 | PRJNA614504 | PRJNA718341 | 17.49 | 17.59 | - |
| USA/WI-UW-77/2020 | 65 | Wisconsin | Dane County | 2020-3-19 | EPI_ISL_421335 | MT706198 | PRJNA614504 | PRJNA718341 | 20.19 | 20.65 | - |
| USA/WI-UW-84/2020 | 74 | Wisconsin | Dane County | 2020-3-24 | EPI_ISL_421343 | MT706205 | PRJNA614504 | PRJNA718341 | 23.12 | 23.82 | - |
| USA/WI-UW-85/2020 | 79 | Wisconsin | Dane County | 2020-4-2 | EPI_ISL_425142 | MT706206 | PRJNA614504 | PRJNA718341 | 24.4 | - | - |
| USA/WI-UW-86/2020 | 80 | Wisconsin | Dane County | 2020-4-2 | EPI_ISL_425143 | MT706207 | PRJNA614504 | PRJNA718341 | 22.1 | - | - |
| USA/WI-UW-87/2020 | 81 | Wisconsin | Dane County | 2020-4-2 | EPI_ISL_425144 | MT706208 | PRJNA614504 | PRJNA718341 | 22.1 | - | - |
| USA/WI-UW-88/2020 | 82 | Wisconsin | Dane County | 2020-4-5 | EPI_ISL_425145 | MT706209 | PRJNA614504 | PRJNA718341 | 25.29 | 25.93 | - |
| USA/WI-UW-96/2020 | 94 | Wisconsin | Dane County | 2020-4-1 | EPI_ISL_425153 | MT706216 | PRJNA614504 | PRJNA718341 | 17.33 | 18.05 | - |
| USA/WI-UW-97/2020 | 95 | Wisconsin | Dane County | 2020-3-30 | EPI_ISL_425154 | MT706217 | PRJNA614504 | PRJNA718341 | 27.3 | - | - |
| USA/WI-UW-99/2020 | 99 | Wisconsin | Dane County | 2020-4-2 | EPI_ISL_425156 | MT706219 | PRJNA614504 | PRJNA718341 | 18.8 | - | - |

| USA/WI-UW-110/2020 | 117 | Wisconsin | Dane County | 2020-3-31 | EPI_ISL_425167 | MT706230 | PRJNA614504 | PRJNA718341 | 22.2 | - | - |
| --- | --- | --- | --- | --- | --- | --- | --- | --- | --- | --- | --- |
| USA/WI-UW-111/2020 | 118 | Wisconsin | Dane County | 2020-3-31 | EPI_ISL_425168 | MT706231 | PRJNA614504 | PRJNA718341 | 25.61 | 26.29 | - |
| USA/WI-UW-116/2020 | 124 | Wisconsin | Dane County | 2020-3-30 | EPI_ISL_425173 | MT706236 | PRJNA614504 | PRJNA718341 | 31.81 | 33.31 | - |
| USA/WI-UW-117/2020 | 125 | Wisconsin | Dane County | 2020-3-30 | EPI_ISL_425174 | MT706237 | PRJNA614504 | PRJNA718341 | 28.2 | - | - |
| USA/WI-UW-119/2020 | 128 | Wisconsin | Dane County | 2020-4-10 | EPI_ISL_425176 | MT706239 | PRJNA614504 | PRJNA718341 | 14.76 | 14.82 | - |
| USA/WI-UW-120/2020 | 130 | Wisconsin | Dane County | 2020-4-13 | EPI_ISL_427427 | MT706240 | PRJNA614504 | PRJNA718341 | 18.3 | - | - |
| USA/WI-UW-255/2020 | 139 | Wisconsin | Dane County | 2020-4-2 | EPI_ISL_428729 | MT706248 | PRJNA614504 | PRJNA718341 | - | - | - |
| USA/WI-UW-124/2020 | 145 | Wisconsin | Dane County | 2020-4-7 | EPI_ISL_427431 | MT706252 | PRJNA614504 | PRJNA718341 | 17.5 | - | - |
| USA/WI-UW-127/2020 | 148 | Wisconsin | Dane County | 2020-4-7 | EPI_ISL_427434 | MT706255 | PRJNA614504 | PRJNA718341 | 19.1 | - | - |
| USA/WI-UW-129/2020 | 151 | Wisconsin | Dane County | 2020-4-6 | EPI_ISL_427436 | MT706257 | PRJNA614504 | PRJNA718341 | 30.7 | - | - |
| USA/WI-UW-132/2020 | 158 | Wisconsin | Rock County | 2020-4-10 | EPI_ISL_427439 | MT706260 | PRJNA614504 | PRJNA718341 | 17.1 | - | - |
| USA/WI-UW-140/2020 | 168 | Wisconsin | Dane County | 2020-4-9 | EPI_ISL_427447 | MT706268 | PRJNA614504 | PRJNA718341 | 21.43 | - | - |
| USA/WI-UW-144/2020 | 172 | Wisconsin | Dane County | 2020-4-6 | EPI_ISL_427451 | MT706272 | PRJNA614504 | PRJNA718341 | 23.92 | 24.31 | - |
| USA/WI-UW-146/2020 | 175 | Wisconsin | Dane County | 2020-4-6 | EPI_ISL_427453 | MT706274 | PRJNA614504 | PRJNA718341 | 26.1 | - | - |
| USA/IL-UW-149/2020 | 182 | Illinois | Winnebago County | 2020-4-7 | EPI_ISL_427456 | MT706277 | PRJNA614504 | PRJNA718341 | 12.6 | - | - |
| USA/WI-UW-154/2020 | 188 | Wisconsin | Monroe County | 2020-4-12 | EPI_ISL_427461 | MT706282 | PRJNA614504 | PRJNA718341 | 21.5 | - | - |
| USA/WI-UW-158/2020 | 195 | Wisconsin | Milwaukee County | 2020-3-15 | EPI_ISL_428254 | MT706286 | PRJNA614504 | PRJNA718341 | 23.93 | 24.33 | - |
| USA/WI-UW-159/2020 | 196 | Wisconsin | Milwaukee County | 2020-3-15 | EPI_ISL_428255 | MT706287 | PRJNA614504 | PRJNA718341 | 20.57 | 21.06 | - |
| USA/WI-UW-160/2020 | 197 | Wisconsin | Milwaukee County | 2020-3-15 | EPI_ISL_428256 | MT706288 | PRJNA614504 | PRJNA718341 | 18.6 | 19.08 | - |
| USA/WI-UW-179/2020 | 218 | Wisconsin | Milwaukee County | 2020-3-21 | EPI_ISL_428275 | MT706307 | PRJNA614504 | PRJNA718341 | 19.89 | 18.58 | - |
| USA/WI-UW-188/2020 | 229 | Wisconsin | Milwaukee County | 2020-3-23 | EPI_ISL_428284 | MT706316 | PRJNA614504 | PRJNA718341 | 30.05 | 29.06 | - |
| USA/WI-UW-201/2020 | 245 | Wisconsin | Ozaukee County | 2020-3-25 | EPI_ISL_428297 | MT706329 | PRJNA614504 | PRJNA718341 | 21.19 | 21.23 | - |

| USA/WI-UW-205/2020 | 249 | Wisconsin | Milwaukee County | 2020-3-25 | EPI_ISL_428301 | MT706333 | PRJNA614504 | PRJNA718341 | 27.03 | 27.23 | - |
| --- | --- | --- | --- | --- | --- | --- | --- | --- | --- | --- | --- |
| USA/WI-UW-208/2020 | 252 | Wisconsin | Milwaukee County | 2020-3-25 | EPI_ISL_428304 | MT706336 | PRJNA614504 | PRJNA718341 | 25.72 | 24.87 | - |
| USA/WI-UW-211/2020 | 255 | Wisconsin | Milwaukee County | 2020-3-25 | EPI_ISL_428307 | MT706339 | PRJNA614504 | PRJNA718341 | 26.72 | 27.21 | - |
| USA/WI-UW-214/2020 | 258 | Wisconsin | Ozaukee County | 2020-3-25 | EPI_ISL_428310 | MT706342 | PRJNA614504 | PRJNA718341 | 21.56 | 21.52 | - |
| USA/WI-UW-217/2020 | 261 | Wisconsin | Milwaukee County | 2020-3-25 | EPI_ISL_428313 | MT706345 | PRJNA614504 | PRJNA718341 | 29.14 | 29.6 | - |
| USA/WI-UW-223/2020 | 268 | Wisconsin | Milwaukee County | 2020-3-26 | EPI_ISL_428319 | MT706351 | PRJNA614504 | PRJNA718341 | 26.67 | 28.25 | - |
| USA/WI-UW-225/2020 | 270 | Wisconsin | Milwaukee County | 2020-3-26 | EPI_ISL_428321 | MT706353 | PRJNA614504 | PRJNA718341 | 18.55 | 18.91 | - |
| USA/WI-UW-230/2020 | 275 | Wisconsin | Milwaukee County | 2020-3-26 | EPI_ISL_428326 | MT706358 | PRJNA614504 | PRJNA718341 | 24.4 | 25.99 | - |
| USA/WI-UW-231/2020 | 276 | Wisconsin | Milwaukee County | 2020-3-26 | EPI_ISL_428327 | MT706359 | PRJNA614504 | PRJNA718341 | 19.42 | 19.94 | - |
| USA/WI-UW-232/2020 | 277 | Wisconsin | Milwaukee County | 2020-3-27 | EPI_ISL_428328 | MT706360 | PRJNA614504 | PRJNA718341 | 19.54 | 18.33 | - |
| USA/WI-UW-238/2020 | 283 | Wisconsin | Milwaukee County | 2020-3-27 | EPI_ISL_428334 | MT706366 | PRJNA614504 | PRJNA718341 | 26.38 | 25.52 | - |
| USA/WI-UW-242/2020 | 288 | Wisconsin | Milwaukee County | 2020-3-28 | EPI_ISL_428338 | MT706370 | PRJNA614504 | PRJNA718341 | 17.93 | 17.52 | - |
| USA/WI-UW-243/2020 | 289 | Wisconsin | Ozaukee County | 2020-3-28 | EPI_ISL_428339 | MT706371 | PRJNA614504 | PRJNA718341 | 25.29 | 25.04 | - |
| USA/WI-UW-244/2020 | 290 | Wisconsin | Ozaukee County | 2020-3-28 | EPI_ISL_428340 | MT706372 | PRJNA614504 | PRJNA718341 | 19.65 | 19.25 | - |
| USA/WI-UW-246/2020 | 292 | Wisconsin | Milwaukee County | 2020-3-28 | EPI_ISL_428342 | - | PRJNA614504 | PRJNA718341 | 22.16 | 21.97 | - |
| Tube-302 | 302 | Wisconsin | Dane County | 2020-4-16 | - | - | - | PRJNA718341 | 29.5 | - | - |
| Tube-303 | 303 | Wisconsin | Monroe County | 2020-4-16 | - | - | - | PRJNA718341 | - | - | - |
| Tube-304 | 304 | Wisconsin | Dane County | 2020-4-19 | - | - | - | PRJNA718341 | 30.7 | - | - |
| USA/WI-UW-348/2020 | 310 | Wisconsin | Dane County | 2020-4-15 | EPI_ISL_450702 | MT506887 | PRJNA614504 | PRJNA718341 | - | - | - |
| USA/WI-UW-351/2020 | 316 | Wisconsin | Dane County | 2020-4-14 | EPI_ISL_450705 | MT506890 | PRJNA614504 | PRJNA718341 | 25.51 | 26.64 | - |
| USA/WI-UW-367/2020 | 338 | Wisconsin | Dane County | 2020-4-1 | EPI_ISL_450721 | MT506906 | PRJNA614504 | PRJNA718341 | - | - | - |
| USA/WI-UW-273/2020 | 356 | Wisconsin | Milwaukee County | 2020-3-31 | EPI_ISL_436567 | MT706381 | PRJNA614504 | PRJNA718341 | 20.29 | 20.47 | - |

| USA/WI-UW-275/2020 | 358 | Wisconsin | Milwaukee County | 2020-4-1 | EPI_ISL_436569 | MT706383 | PRJNA614504 | PRJNA718341 | 18.54 | 18.14 | - |
| --- | --- | --- | --- | --- | --- | --- | --- | --- | --- | --- | --- |
| USA/WI-UW-276/2020 | 359 | Wisconsin | Milwaukee County | 2020-4-1 | EPI_ISL_436570 | MT706384 | PRJNA614504 | PRJNA718341 | 19.27 | 19.02 | - |
| USA/WI-UW-277/2020 | 361 | Wisconsin | Milwaukee County | 2020-4-2 | EPI_ISL_436571 | MT706385 | PRJNA614504 | PRJNA718341 | 20.28 | 20.06 | - |
| USA/WI-UW-278/2020 | 365 | Wisconsin | Milwaukee County | 2020-4-3 | EPI_ISL_436572 | MT706386 | PRJNA614504 | PRJNA718341 | 16.08 | 16.12 | - |
| USA/WI-UW-279/2020 | 366 | Wisconsin | Milwaukee County | 2020-4-3 | EPI_ISL_436573 | MT706387 | PRJNA614504 | PRJNA718341 | 15.6 | 15.35 | - |
| USA/WI-UW-282/2020 | 370 | Wisconsin | Milwaukee County | 2020-4-6 | EPI_ISL_436576 | MT706390 | PRJNA614504 | PRJNA718341 | 15.39 | 14.91 | - |
| USA/WI-UW-285/2020 | 374 | Wisconsin | Milwaukee County | 2020-4-6 | EPI_ISL_436579 | MT706393 | PRJNA614504 | PRJNA718341 | 27.55 | 27.17 | - |
| USA/WI-UW-286/2020 | 376 | Wisconsin | Milwaukee County | 2020-4-6 | EPI_ISL_436580 | MT706394 | PRJNA614504 | PRJNA718341 | 25.2 | 25.09 | - |
| USA/WI-UW-287/2020 | 377 | Wisconsin | Milwaukee County | 2020-4-6 | EPI_ISL_436581 | MT706395 | PRJNA614504 | PRJNA718341 | 23.43 | 23.35 | - |
| USA/WI-UW-296/2020 | 388 | Wisconsin | Milwaukee County | 2020-4-9 | EPI_ISL_436590 | MT706404 | PRJNA614504 | PRJNA718341 | 16.13 | 15.52 | - |
| USA/WI-UW-299/2020 | 391 | Wisconsin | Milwaukee County | 2020-4-13 | EPI_ISL_436593 | MT706407 | PRJNA614504 | PRJNA718341 | 18.84 | 18.37 | - |
| USA/WI-UW-302/2020 | 397 | Wisconsin | Milwaukee County | 2020-4-13 | EPI_ISL_436596 | MT706410 | PRJNA614504 | PRJNA718341 | 17.6 | 17.08 | - |
| USA/WI-UW-306/2020 | 402 | Wisconsin | Milwaukee County | 2020-4-14 | EPI_ISL_436600 | MT706414 | PRJNA614504 | PRJNA718341 | 30.42 | 31.22 | - |
| USA/WI-UW-310/2020 | 408 | Wisconsin | Milwaukee County | 2020-4-15 | EPI_ISL_436604 | MT706418 | PRJNA614504 | PRJNA718341 | 20.64 | 19.45 | - |
| USA/WI-UW-315/2020 | 418 | Wisconsin | Milwaukee County | 2020-4-17 | EPI_ISL_436609 | MT706423 | PRJNA614504 | PRJNA718341 | 26.33 | 26.42 | - |
| USA/WI-UW-323/2020 | 437 | Wisconsin | Milwaukee County | 2020-4-23 | EPI_ISL_436617 | MT706430 | PRJNA614504 | PRJNA718341 | 26.2 | 28.6 | - |
| USA/WI-UW-333/2020 | 453 | Wisconsin | Milwaukee County | 2020-3-24 | EPI_ISL_436627 | MT706439 | PRJNA614504 | PRJNA718341 | 27.45 | 26.54 | - |
| USA/WI-UW-334/2020 | 454 | Wisconsin | Milwaukee County | 2020-3-24 | EPI_ISL_436628 | MT706440 | PRJNA614504 | PRJNA718341 | 23.24 | 22.79 | - |
| USA/WI-UW-337/2020 | 461 | Wisconsin | Milwaukee County | 2020-3-26 | EPI_ISL_436631 | MT706443 | PRJNA614504 | PRJNA718341 | 26.46 | 26.5 | - |
| USA/WI-UW-338/2020 | 462 | Wisconsin | Milwaukee County | 2020-3-26 | EPI_ISL_436632 | MT706444 | PRJNA614504 | PRJNA718341 | 27.72 | 27.6 | - |
| USA/WI-UW-340/2020 | 468 | Wisconsin | Milwaukee County | 2020-4-1 | EPI_ISL_436634 | MT706446 | PRJNA614504 | PRJNA718341 | 35.15 | 33.47 | - |
| USA/WI-UW-341/2020 | 469 | Wisconsin | Milwaukee County | 2020-4-2 | EPI_ISL_436635 | MT706447 | PRJNA614504 | PRJNA718341 | 23.18 | 22.53 | - |

| USA/WI-UW-389/2020 | 552 | Wisconsin | Dane County | 2020-5-26 | EPI_ISL_480371 | MT772540 | PRJNA614504 | PRJNA718341 | 23.2 | - | - |
| --- | --- | --- | --- | --- | --- | --- | --- | --- | --- | --- | --- |
| USA/WI-UW-391/2020 | 554 | Wisconsin | Dane County | 2020-5-26 | EPI_ISL_480373 | MT772542 | PRJNA614504 | PRJNA718341 | 17.7 | - | - |
| USA/WI-UW-432/2020 | 738 | Wisconsin | Dane County | 2020-6-17 | EPI_ISL_484807 | MT750020 | PRJNA614504 | PRJNA718341 | 21.6 | - | - |
| USA/WI-UW-438/2020 | 744 | Wisconsin | Dane County | 2020-6-15 | EPI_ISL_484813 | MT750026 | PRJNA614504 | PRJNA718341 | 19.4 | - | - |
| USA/WI-UW-443/2020 | 749 | Wisconsin | Dane County | 2020-6-19 | EPI_ISL_484818 | MT750030 | PRJNA614504 | PRJNA718341 | 19.4 | - | - |
| USA/WI-UW-476/2020 | 783 | Wisconsin | Dane County | 2020-6-12 | EPI_ISL_484851 | MT750060 | PRJNA614504 | PRJNA718341 | 20.6 | - | - |
| USA/WI-UW-536/2020 | 849 | Wisconsin | Dane County | 2020-6-24 | EPI_ISL_484911 | MT750116 | PRJNA614504 | PRJNA718341 | - | - | 1211 |
| USA/WI-UW-544/2020 | 884 | Wisconsin | Dane County | 2020-6-22 | EPI_ISL_484919 | MT750124 | PRJNA614504 | PRJNA718341 | - | - | 1334 |
| USA/WI-UW-546/2020 | 887 | Wisconsin | Dane County | 2020-6-19 | EPI_ISL_484921 | MT750126 | PRJNA614504 | PRJNA718341 | - | - | 1282 |
| USA/WI-UW-551/2020 | 893 | Wisconsin | Dane County | 2020-6-23 | EPI_ISL_484926 | MT750131 | PRJNA614504 | PRJNA718341 | - | - | 1254 |
| USA/WI-UW-575/2020 | 903 | Wisconsin | Dane County | 2020-6-23 | EPI_ISL_484950 | MT750154 | PRJNA614504 | PRJNA718341 | - | - | 1238 |
| USA/WI-UW-577/2020 | 906 | Wisconsin | Dane County | 2020-6-24 | EPI_ISL_484952 | MT750156 | PRJNA614504 | PRJNA718341 | - | - | 1288 |
| USA/WI-UW-586/2020 | 916 | Wisconsin | Dane County | 2020-6-19 | EPI_ISL_484961 | MT750165 | PRJNA614504 | PRJNA718341 | - | - | 1274 |
| USA/WI-UW-598/2020 | 956 | Wisconsin | Dane County | 2020-6-27 | EPI_ISL_484973 | MT750176 | PRJNA614504 | PRJNA718341 | - | - | 1240 |
| USA/WI-UW-601/2020 | 961 | Wisconsin | Dane County | 2020-6-25 | EPI_ISL_484976 | MT750179 | PRJNA614504 | PRJNA718341 | - | - | 1155 |
| USA/WI-UW-602/2020 | 962 | Wisconsin | Dane County | 2020-6-28 | EPI_ISL_484977 | - | PRJNA614504 | PRJNA718341 | - | - | 1203 |
| USA/WI-UW-689/2020 | 1049 | Wisconsin | Dane County | 2020-6-28 | EPI_ISL_491369 | MT772466 | PRJNA614504 | PRJNA718341 | - | - | 1217 |
| USA/WI-UW-694/2020 | 1061 | Wisconsin | Dane County | 2020-6-30 | EPI_ISL_491372 | - | PRJNA614504 | PRJNA718341 | - | - | 1218 |
| USA/WI-UW-697/2020 | 1064 | Wisconsin | Dane County | 2020-7-2 | EPI_ISL_491375 | MT772473 | PRJNA614504 | PRJNA718341 | - | - | 1257 |
| USA/WI-UW-721/2020 | 1103 | Wisconsin | Dane County | 2020-7-1 | EPI_ISL_491396 | - | PRJNA614504 | PRJNA718341 | - | - | 1177 |
| USA/WI-UW-722/2020 | 1104 | Wisconsin | Dane County | 2020-6-30 | EPI_ISL_491397 | - | PRJNA614504 | PRJNA718341 | - | - | 1276 |
| USA/WI-UW-747/2020 | 1144 | Wisconsin | Dane County | 2020-7-2 | EPI_ISL_491420 | MT772518 | PRJNA614504 | PRJNA718341 | - | - | 1222 |

| USA/WI-UW-749/2020 | 1147 | Wisconsin | Dane County | 2020-6-30 | EPI_ISL_491422 | - | PRJNA614504 | PRJNA718341 | - | - | 1287 |
| --- | --- | --- | --- | --- | --- | --- | --- | --- | --- | --- | --- |
| USA/WI-UW-756/2020 | 1157 | Wisconsin | Dane County | 2020-7-5 | EPI_ISL_495461 | MT795871 | PRJNA614504 | PRJNA718341 | - | - | 1233 |
| USA/WI-UW-780/2020 | 1195 | Wisconsin | Dane County | 2020-7-3 | EPI_ISL_495484 | MT795891 | PRJNA614504 | PRJNA718341 | - | - | 1269 |
| USA/WI-UW-784/2020 | 1199 | Wisconsin | Dane County | 2020-7-6 | EPI_ISL_495488 | - | PRJNA614504 | PRJNA718341 | - | - | 1238 |
| USA/WI-UW-798/2020 | 1217 | Wisconsin | Dane County | 2020-7-6 | EPI_ISL_495502 | - | PRJNA614504 | PRJNA718341 | - | - | 1223 |
| USA/WI-UW-855/2020 | 1282 | Wisconsin | Dane County | 2020-7-9 | EPI_ISL_509861 | MT846545 | PRJNA614504 | PRJNA718341 | - | - | 1247 |
| USA/WI-UW-861/2020 | 1293 | Wisconsin | Dane County | 2020-7-14 | EPI_ISL_509864 | MT846550 | PRJNA614504 | PRJNA718341 | - | - | 1210 |
| USA/WI-UW-863/2020 | 1297 | Wisconsin | Dane County | 2020-7-13 | EPI_ISL_509866 | MT846552 | PRJNA614504 | PRJNA718341 | - | - | 1159 |
| USA/WI-UW-874/2020 | 1326 | Wisconsin | Dane County | 2020-7-13 | EPI_ISL_509876 | MT846562 | PRJNA614504 | PRJNA718341 | - | - | 1227 |
| USA/WI-UW-876/2020 | 1328 | Wisconsin | Dane County | 2020-7-13 | EPI_ISL_509878 | MT846564 | PRJNA614504 | PRJNA718341 | - | - | 1241 |
| USA/WI-UW-893/2020 | 1346 | Wisconsin | Dane County | 2020-7-12 | EPI_ISL_509895 | MT846581 | PRJNA614504 | PRJNA718341 | - | - | 1181 |
| USA/WI-UW-895/2020 | 1353 | Wisconsin | Dane County | 2020-7-13 | EPI_ISL_509897 | MT846583 | PRJNA614504 | PRJNA718341 | - | - | 1200 |
| USA/WI-UW-897/2020 | 1357 | Wisconsin | Dane County | 2020-7-12 | EPI_ISL_509899 | MT846585 | PRJNA614504 | PRJNA718341 | - | - | 1224 |
| USA/WI-UW-906/2020 | 1373 | Wisconsin | Dane County | 2020-7-15 | EPI_ISL_509907 | MT846593 | PRJNA614504 | PRJNA718341 | - | - | 1246 |
| USA/WI-UW-916/2020 | 1388 | Wisconsin | Dane County | 2020-7-12 | EPI_ISL_509917 | MT846603 | PRJNA614504 | PRJNA718341 | - | - | 1209 |
| USA/WI-UW-927/2020 | 1409 | Wisconsin | Dane County | 2020-7-13 | EPI_ISL_509927 | MT846614 | PRJNA614504 | PRJNA718341 | - | - | 1160 |
| USA/WI-UW-931/2020 | 1414 | Wisconsin | Dane County | 2020-7-13 | EPI_ISL_509931 | MT846618 | PRJNA614504 | PRJNA718341 | - | - | 1221 |
| USA/WI-UW-986/2020 | 1495 | Wisconsin | Dane County | 2020-7-16 | EPI_ISL_509982 | MT846672 | PRJNA614504 | PRJNA718341 | - | - | 1267 |
| USA/WI-UW-991/2020 | 1502 | Wisconsin | Dane County | 2020-7-16 | EPI_ISL_509986 | MT846677 | PRJNA614504 | PRJNA718341 | - | - | 1265 |
| USA/WI-UW-997/2020 | 1512 | Wisconsin | Dane County | 2020-7-16 | EPI_ISL_509991 | MT846683 | PRJNA614504 | PRJNA718341 | - | - | 1220 |
