# Supplementary material for: Acute SARS-CoV-2 infections harbor limited within-host diversity and transmit via tight transmission bottlenecks
Source: PLoS Pathog. 2021 Aug 23;17(8):e1009849. doi: 10.1371/journal.ppat.1009849 (PMC8412271; doi:10.1371/journal.ppat.1009849)
Supplement: S3 Table — (DOCX) [file ppat.1009849.s015.docx]

| **name** | **pool** | **sequence** | **length** | **%gc** | **tm (use 65)** |
| --- | --- | --- | --- | --- | --- |
| nCoV-2019_1_LEFT | nCoV-2019_1 | ACCAACCAACTTTCGATCTCTTGT | 24 | 41.67 | 60.69 |
| nCoV-2019_1_RIGHT | nCoV-2019_1 | CATCTTTAAGATGTTGACGTGCCTC | 25 | 44 | 60.45 |
| nCoV-2019_2_LEFT | nCoV-2019_2 | CTGTTTTACAGGTTCGCGACGT | 22 | 50 | 61.67 |
| nCoV-2019_2_RIGHT | nCoV-2019_2 | TAAGGATCAGTGCCAAGCTCGT | 22 | 50 | 61.74 |
| nCoV-2019_3_LEFT | nCoV-2019_1 | CGGTAATAAAGGAGCTGGTGGC | 22 | 54.55 | 61.32 |
| nCoV-2019_3_RIGHT | nCoV-2019_1 | AAGGTGTCTGCAATTCATAGCTCT | 24 | 41.67 | 60.32 |
| nCoV-2019_4_LEFT | nCoV-2019_2 | GGTGTATACTGCTGCCGTGAAC | 22 | 54.55 | 61.56 |
| nCoV-2019_4_RIGHT | nCoV-2019_2 | CACAAGTAGTGGCACCTTCTTTAGT | 25 | 44 | 60.97 |
| nCoV-2019_5_LEFT | nCoV-2019_1 | TGGTGAAACTTCATGGCAGACG | 22 | 50 | 61.39 |
| nCoV-2019_5_RIGHT | nCoV-2019_1 | ATTGATGTTGACTTTCTCTTTTTGGAGT | 28 | 32.14 | 60.17 |
| nCoV-2019_6_LEFT | nCoV-2019_2 | GGTGTTGTTGGAGAAGGTTCCG | 22 | 54.55 | 61.64 |
| nCoV-2019_6_RIGHT | nCoV-2019_2 | TAGCGGCCTTCTGTAAAACACG | 22 | 50 | 61.18 |
| nCoV-2019_7_LEFT | nCoV-2019_1 | ATCAGAGGCTGCTCGTGTTGTA | 22 | 50 | 61.73 |
| nCoV-2019_7_LEFT_alt0 | nCoV-2019_1 | CATTTGCATCAGAGGCTGCTCG | 22 | 54.55 | 62.44 |
| nCoV-2019_7_RIGHT | nCoV-2019_1 | TGCACAGGTGACAATTTGTCCA | 22 | 45.45 | 60.95 |
| nCoV-2019_7_RIGHT_alt5 | nCoV-2019_1 | AGGTGACAATTTGTCCACCGAC | 22 | 50 | 61.07 |
| nCoV-2019_8_LEFT | nCoV-2019_2 | AGAGTTTCTTAGAGACGGTTGGGA | 24 | 45.83 | 61 |
| nCoV-2019_8_RIGHT | nCoV-2019_2 | GCTTCAACAGCTTCACTAGTAGGT | 24 | 45.83 | 60.56 |
| nCoV-2019_9_LEFT | nCoV-2019_1 | TCCCACAGAAGTGTTAACAGAGGA | 24 | 45.83 | 61.18 |
| nCoV-2019_9_LEFT_alt4 | nCoV-2019_1 | TTCCCACAGAAGTGTTAACAGAGG | 24 | 45.83 | 60.44 |
| nCoV-2019_9_RIGHT | nCoV-2019_1 | ATGACAGCATCTGCCACAACAC | 22 | 50 | 61.71 |
| nCoV-2019_9_RIGHT_alt2 | nCoV-2019_1 | GACAGCATCTGCCACAACACAG | 22 | 54.55 | 62.26 |
| nCoV-2019_10_LEFT | nCoV-2019_2 | TGAGAAGTGCTCTGCCTATACAGT | 24 | 45.83 | 61.12 |
| nCoV-2019_10_RIGHT | nCoV-2019_2 | TCATCTAACCAATCTTCTTCTTGCTCT | 27 | 37.04 | 60.31 |
| nCoV-2019_11_LEFT | nCoV-2019_1 | GGAATTTGGTGCCACTTCTGCT | 22 | 50 | 61.66 |
| nCoV-2019_11_RIGHT | nCoV-2019_1 | TCATCAGATTCAACTTGCATGGCA | 24 | 41.67 | 61.35 |
| nCoV-2019_12_LEFT | nCoV-2019_2 | AAACATGGAGGAGGTGTTGCAG | 22 | 50 | 61.08 |
| nCoV-2019_12_RIGHT | nCoV-2019_2 | TTCACTCTTCATTTCCAAAAAGCTTGA | 27 | 33.33 | 60.36 |
| nCoV-2019_13_LEFT | nCoV-2019_1 | TCGCACAAATGTCTACTTAGCTGT | 24 | 41.67 | 60.56 |

| nCoV-2019_13_RIGHT | nCoV-2019_1 | ACCACAGCAGTTAAAACACCCT | 22 | 45.45 | 60.36 |
| --- | --- | --- | --- | --- | --- |
| nCoV-2019_14_LEFT | nCoV-2019_2 | CATCCAGATTCTGCCACTCTTGT | 23 | 47.83 | 60.62 |
| nCoV-2019_14_LEFT_alt4 | nCoV-2019_2 | TGGCAATCTTCATCCAGATTCTGC | 24 | 45.83 | 61.47 |
| nCoV-2019_14_RIGHT | nCoV-2019_2 | AGTTTCCACACAGACAGGCATT | 22 | 45.45 | 60.42 |
| nCoV-2019_14_RIGHT_alt2 | nCoV-2019_2 | TGCGTGTTTCTTCTGCATGTGC | 22 | 50 | 62.76 |
| nCoV-2019_15_LEFT | nCoV-2019_1 | ACAGTGCTTAAAAAGTGTAAAAGTGCC | 27 | 37.04 | 61.32 |
| nCoV-2019_15_LEFT_alt1 | nCoV-2019_1 | AGTGCTTAAAAAGTGTAAAAGTGCCT | 26 | 34.62 | 60.13 |
| nCoV-2019_15_RIGHT | nCoV-2019_1 | AACAGAAACTGTAGCTGGCACT | 22 | 45.45 | 60.16 |
| nCoV-2019_15_RIGHT_alt3 | nCoV-2019_1 | ACTGTAGCTGGCACTTTGAGAGA | 23 | 47.83 | 61.57 |
| nCoV-2019_16_LEFT | nCoV-2019_2 | AATTTGGAAGAAGCTGCTCGGT | 22 | 45.45 | 60.82 |
| nCoV-2019_16_RIGHT | nCoV-2019_2 | CACAACTTGCGTGTGGAGGTTA | 22 | 50 | 61.32 |
| nCoV-2019_17_LEFT | nCoV-2019_1 | CTTCTTTCTTTGAGAGAAGTGAGGACT | 27 | 40.74 | 60.69 |
| nCoV-2019_17_RIGHT | nCoV-2019_1 | TTTGTTGGAGTGTTAACAATGCAGT | 25 | 36 | 60.11 |
| nCoV-2019_18_LEFT | nCoV-2019_2 | TGGAAATACCCACAAGTTAATGGTTTAAC | 29 | 34.48 | 60.69 |
| nCoV-2019_18_LEFT_alt2 | nCoV-2019_2 | ACTTCTATTAAATGGGCAGATAACAACTGT | 30 | 33.33 | 61.38 |
| nCoV-2019_18_RIGHT | nCoV-2019_2 | AGCTTGTTTACCACACGTACAAGG | 24 | 45.83 | 61.51 |
| nCoV-2019_18_RIGHT_alt1 | nCoV-2019_2 | GCTTGTTTACCACACGTACAAGG | 23 | 47.83 | 60.3 |
| nCoV-2019_19_LEFT | nCoV-2019_1 | GCTGTTATGTACATGGGCACACT | 23 | 47.83 | 61.18 |
| nCoV-2019_19_RIGHT | nCoV-2019_1 | TGTCCAACTTAGGGTCAATTTCTGT | 25 | 40 | 60.4 |
| nCoV-2019_20_LEFT | nCoV-2019_2 | ACAAAGAAAACAGTTACACAACAACCA | 27 | 33.33 | 60.68 |
| nCoV-2019_20_RIGHT | nCoV-2019_2 | ACGTGGCTTTATTAGTTGCATTGTT | 25 | 36 | 60.28 |
| nCoV-2019_21_LEFT | nCoV-2019_1 | TGGCTATTGATTATAAACACTACACACCC | 29 | 37.93 | 61.49 |
| nCoV-2019_21_LEFT_alt2 | nCoV-2019_1 | GGCTATTGATTATAAACACTACACACCCT | 29 | 37.93 | 61.29 |
| nCoV-2019_21_RIGHT | nCoV-2019_1 | TAGATCTGTGTGGCCAACCTCT | 22 | 50 | 60.83 |
| nCoV-2019_21_RIGHT_alt0 | nCoV-2019_1 | GATCTGTGTGGCCAACCTCTTC | 22 | 54.55 | 61.2 |
| nCoV-2019_22_LEFT | nCoV-2019_2 | ACTACCGAAGTTGTAGGAGACATTATACT | 29 | 37.93 | 61.25 |
| nCoV-2019_22_RIGHT | nCoV-2019_2 | ACAGTATTCTTTGCTATAGTAGTCGGC | 27 | 40.74 | 60.73 |
| nCoV-2019_23_LEFT | nCoV-2019_1 | ACAACTACTAACATAGTTACACGGTGT | 27 | 37.04 | 60.26 |
| nCoV-2019_23_RIGHT | nCoV-2019_1 | ACCAGTACAGTAGGTTGCAATAGTG | 25 | 44 | 60.57 |
| nCoV-2019_24_LEFT | nCoV-2019_2 | AGGCATGCCTTCTTACTGTACTG | 23 | 47.83 | 60.37 |
| nCoV-2019_24_RIGHT | nCoV-2019_2 | ACATTCTAACCATAGCTGAAATCGGG | 26 | 42.31 | 61.19 |
| nCoV-2019_25_LEFT | nCoV-2019_1 | GCAATTGTTTTTCAGCTATTTTGCAGT | 27 | 33.33 | 60.73 |

| nCoV-2019_25_RIGHT | nCoV-2019_1 | ACTGTAGTGACAAGTCTCTCGCA | 23 | 47.83 | 61.3 |
| --- | --- | --- | --- | --- | --- |
| nCoV-2019_26_LEFT | nCoV-2019_2 | TTGTGATACATTCTGTGCTGGTAGT | 25 | 40 | 60.28 |
| nCoV-2019_26_RIGHT | nCoV-2019_2 | TCCGCACTATCACCAACATCAG | 22 | 50 | 60.42 |
| nCoV-2019_27_LEFT | nCoV-2019_1 | ACTACAGTCAGCTTATGTGTCAACC | 25 | 44 | 60.8 |
| nCoV-2019_27_RIGHT | nCoV-2019_1 | AATACAAGCACCAAGGTCACGG | 22 | 50 | 61.13 |
| nCoV-2019_28_LEFT | nCoV-2019_2 | ACATAGAAGTTACTGGCGATAGTTGT | 26 | 38.46 | 60.13 |
| nCoV-2019_28_RIGHT | nCoV-2019_2 | TGTTTAGACATGACATGAACAGGTGT | 26 | 38.46 | 60.91 |
| nCoV-2019_29_LEFT | nCoV-2019_1 | ACTTGTGTTCCTTTTTGTTGCTGC | 24 | 41.67 | 61.39 |
| nCoV-2019_29_RIGHT | nCoV-2019_1 | AGTGTACTCTATAAGTTTTGATGGTGTGT | 29 | 34.48 | 60.69 |
| nCoV-2019_30_LEFT | nCoV-2019_2 | GCACAACTAATGGTGACTTTTTGCA | 25 | 40 | 61.19 |
| nCoV-2019_30_RIGHT | nCoV-2019_2 | ACCACTAGTAGATACACAAACACCAG | 26 | 42.31 | 60.3 |
| nCoV-2019_31_LEFT | nCoV-2019_1 | TTCTGAGTACTGTAGGCACGGC | 22 | 54.55 | 62.03 |
| nCoV-2019_31_RIGHT | nCoV-2019_1 | ACAGAATAAACACCAGGTAAGAATGAGT | 28 | 35.71 | 60.69 |
| nCoV-2019_32_LEFT | nCoV-2019_2 | TGGTGAATACAGTCATGTAGTTGCC | 25 | 44 | 61.09 |
| nCoV-2019_32_RIGHT | nCoV-2019_2 | AGCACATCACTACGCAACTTTAGA | 24 | 41.67 | 60.56 |
| nCoV-2019_33_LEFT | nCoV-2019_1 | ACTTTTGAAGAAGCTGCGCTGT | 22 | 45.45 | 61.58 |
| nCoV-2019_33_RIGHT | nCoV-2019_1 | TGGACAGTAAACTACGTCATCAAGC | 25 | 44 | 61.08 |
| nCoV-2019_34_LEFT | nCoV-2019_2 | TCCCATCTGGTAAAGTTGAGGGT | 23 | 47.83 | 61.02 |
| nCoV-2019_34_RIGHT | nCoV-2019_2 | AGTGAAATTGGGCCTCATAGCA | 22 | 45.45 | 60.03 |
| nCoV-2019_35_LEFT | nCoV-2019_1 | TGTTCGCATTCAACCAGGACAG | 22 | 50 | 61.39 |
| nCoV-2019_35_RIGHT | nCoV-2019_1 | ACTTCATAGCCACAAGGTTAAAGTCA | 26 | 38.46 | 60.69 |
| nCoV-2019_36_LEFT | nCoV-2019_2 | TTAGCTTGGTTGTACGCTGCTG | 22 | 50 | 61.44 |
| nCoV-2019_36_RIGHT | nCoV-2019_2 | GAACAAAGACCATTGAGTACTCTGGA | 26 | 42.31 | 60.74 |
| nCoV-2019_37_LEFT | nCoV-2019_1 | ACACACCACTGGTTGTTACTCAC | 23 | 47.83 | 60.93 |
| nCoV-2019_37_RIGHT | nCoV-2019_1 | GTCCACACTCTCCTAGCACCAT | 22 | 54.55 | 61.48 |
| nCoV-2019_38_LEFT | nCoV-2019_2 | ACTGTGTTATGTATGCATCAGCTGT | 25 | 40 | 60.86 |
| nCoV-2019_38_RIGHT | nCoV-2019_2 | CACCAAGAGTCAGTCTAAAGTAGCG | 25 | 48 | 61.13 |
| nCoV-2019_39_LEFT | nCoV-2019_1 | AGTATTGCCCTATTTTCTTCATAACTGGT | 29 | 34.48 | 61 |
| nCoV-2019_39_RIGHT | nCoV-2019_1 | TGTAACTGGACACATTGAGCCC | 22 | 50 | 60.55 |
| nCoV-2019_40_LEFT | nCoV-2019_2 | TGCACATCAGTAGTCTTACTCTCAGT | 26 | 42.31 | 61.25 |
| nCoV-2019_40_RIGHT | nCoV-2019_2 | CATGGCTGCATCACGGTCAAAT | 22 | 50 | 62.09 |
| nCoV-2019_41_LEFT | nCoV-2019_1 | GTTCCCTTCCATCATATGCAGCT | 23 | 47.83 | 60.75 |

| nCoV-2019_41_RIGHT | nCoV-2019_1 | TGGTATGACAACCATTAGTTTGGCT | 25 | 40 | 60.75 |
| --- | --- | --- | --- | --- | --- |
| nCoV-2019_42_LEFT | nCoV-2019_2 | TGCAAGAGATGGTTGTGTTCCC | 22 | 50 | 61.08 |
| nCoV-2019_42_RIGHT | nCoV-2019_2 | CCTACCTCCCTTTGTTGTGTTGT | 23 | 47.83 | 60.69 |
| nCoV-2019_43_LEFT | nCoV-2019_1 | TACGACAGATGTCTTGTGCTGC | 22 | 50 | 60.93 |
| nCoV-2019_43_RIGHT | nCoV-2019_1 | AGCAGCATCTACAGCAAAAGCA | 22 | 45.45 | 61.14 |
| nCoV-2019_44_LEFT | nCoV-2019_2 | TGCCACAGTACGTCTACAAGCT | 22 | 50 | 61.66 |
| nCoV-2019_44_LEFT_alt3 | nCoV-2019_2 | CCACAGTACGTCTACAAGCTGG | 22 | 54.55 | 60.67 |
| nCoV-2019_44_RIGHT | nCoV-2019_2 | AACCTTTCCACATACCGCAGAC | 22 | 50 | 60.87 |
| nCoV-2019_44_RIGHT_alt0 | nCoV-2019_2 | CGCAGACGGTACAGACTGTGTT | 22 | 54.55 | 62.77 |
| nCoV-2019_45_LEFT | nCoV-2019_1 | TACCTACAACTTGTGCTAATGACCC | 25 | 44 | 60.57 |
| nCoV-2019_45_LEFT_alt2 | nCoV-2019_1 | AGTATGTACAAATACCTACAACTTGTGCT | 29 | 34.48 | 60.94 |
| nCoV-2019_45_RIGHT | nCoV-2019_1 | AAATTGTTTCTTCATGTTGGTAGTTAGAGA | 30 | 30 | 60.01 |
| nCoV-2019_45_RIGHT_alt7 | nCoV-2019_1 | TTCATGTTGGTAGTTAGAGAAAGTGTGTC | 29 | 37.93 | 61.53 |
| nCoV-2019_46_LEFT | nCoV-2019_2 | TGTCGCTTCCAAGAAAAGGACG | 22 | 50 | 61.38 |
| nCoV-2019_46_LEFT_alt1 | nCoV-2019_2 | CGCTTCCAAGAAAAGGACGAAGA | 23 | 47.83 | 61.35 |
| nCoV-2019_46_RIGHT | nCoV-2019_2 | CACGTTCACCTAAGTTGGCGTA | 22 | 50 | 60.86 |
| nCoV-2019_46_RIGHT_alt2 | nCoV-2019_2 | CACGTTCACCTAAGTTGGCGTAT | 23 | 47.83 | 61.17 |
| nCoV-2019_47_LEFT | nCoV-2019_1 | AGGACTGGTATGATTTTGTAGAAAACCC | 28 | 39.29 | 61.42 |
| nCoV-2019_47_RIGHT | nCoV-2019_1 | AATAACGGTCAAAGAGTTTTAACCTCTC | 28 | 35.71 | 60.06 |
| nCoV-2019_48_LEFT | nCoV-2019_2 | TGTTGACACTGACTTAACAAAGCCT | 25 | 40 | 61.09 |
| nCoV-2019_48_RIGHT | nCoV-2019_2 | TAGATTACCAGAAGCAGCGTGC | 22 | 50 | 60.74 |
| nCoV-2019_49_LEFT | nCoV-2019_1 | AGGAATTACTTGTGTATGCTGCTGA | 25 | 40 | 60.57 |
| nCoV-2019_49_RIGHT | nCoV-2019_1 | TGACGATGACTTGGTTAGCATTAATACA | 28 | 35.71 | 61.05 |
| nCoV-2019_50_LEFT | nCoV-2019_2 | GTTGATAAGTACTTTGATTGTTACGATGGT | 30 | 33.33 | 60.59 |
| nCoV-2019_50_RIGHT | nCoV-2019_2 | TAACATGTTGTGCCAACCACCA | 22 | 45.45 | 60.95 |
| nCoV-2019_51_LEFT | nCoV-2019_1 | TCAATAGCCGCCACTAGAGGAG | 22 | 54.55 | 61.34 |
| nCoV-2019_51_RIGHT | nCoV-2019_1 | AGTGCATTAACATTGGCCGTGA | 22 | 45.45 | 61.14 |
| nCoV-2019_52_LEFT | nCoV-2019_2 | CATCAGGAGATGCCACAACTGC | 22 | 54.55 | 61.83 |
| nCoV-2019_52_RIGHT | nCoV-2019_2 | GTTGAGAGCAAAATTCATGAGGTCC | 25 | 44 | 60.62 |
| nCoV-2019_53_LEFT | nCoV-2019_1 | AGCAAAATGTTGGACTGAGACTGA | 24 | 41.67 | 60.69 |
| nCoV-2019_53_RIGHT | nCoV-2019_1 | AGCCTCATAAAACTCAGGTTCCC | 23 | 47.83 | 60.31 |
| nCoV-2019_54_LEFT | nCoV-2019_2 | TGAGTTAACAGGACACATGTTAGACA | 26 | 38.46 | 60.18 |

| nCoV-2019_54_RIGHT | nCoV-2019_2 | AACCAAAAACTTGTCCATTAGCACA | 25 | 36 | 60.11 |
| --- | --- | --- | --- | --- | --- |
| nCoV-2019_55_LEFT | nCoV-2019_1 | ACTCAACTTTACTTAGGAGGTATGAGCT | 28 | 39.29 | 61.43 |
| nCoV-2019_55_RIGHT | nCoV-2019_1 | GGTGTACTCTCCTATTTGTACTTTACTGT | 29 | 37.93 | 60.54 |
| nCoV-2019_56_LEFT | nCoV-2019_2 | ACCTAGACCACCACTTAACCGA | 22 | 50 | 60.49 |
| nCoV-2019_56_RIGHT | nCoV-2019_2 | ACACTATGCGAGCAGAAGGGTA | 22 | 50 | 61.21 |
| nCoV-2019_57_LEFT | nCoV-2019_1 | ATTCTACACTCCAGGGACCACC | 22 | 54.55 | 61.16 |
| nCoV-2019_57_RIGHT | nCoV-2019_1 | GTAATTGAGCAGGGTCGCCAAT | 22 | 50 | 61.26 |
| nCoV-2019_58_LEFT | nCoV-2019_2 | TGATTTGAGTGTTGTCAATGCCAGA | 25 | 40 | 61.44 |
| nCoV-2019_58_RIGHT | nCoV-2019_2 | CTTTTCTCCAAGCAGGGTTACGT | 23 | 47.83 | 61.06 |
| nCoV-2019_59_LEFT | nCoV-2019_1 | TCACGCATGATGTTTCATCTGCA | 23 | 43.48 | 61.42 |
| nCoV-2019_59_RIGHT | nCoV-2019_1 | AAGAGTCCTGTTACATTTTCAGCTTG | 26 | 38.46 | 60.02 |
| nCoV-2019_60_LEFT | nCoV-2019_2 | TGATAGAGACCTTTATGACAAGTTGCA | 27 | 37.04 | 60.53 |
| nCoV-2019_60_RIGHT | nCoV-2019_2 | GGTACCAACAGCTTCTCTAGTAGC | 24 | 50 | 60.44 |
| nCoV-2019_61_LEFT | nCoV-2019_1 | TGTTTATCACCCGCGAAGAAGC | 22 | 50 | 61.5 |
| nCoV-2019_61_RIGHT | nCoV-2019_1 | ATCACATAGACAACAGGTGCGC | 22 | 50 | 61.25 |
| nCoV-2019_62_LEFT | nCoV-2019_2 | GGCACATGGCTTTGAGTTGACA | 22 | 50 | 61.91 |
| nCoV-2019_62_RIGHT | nCoV-2019_2 | GTTGAACCTTTCTACAAGCCGC | 22 | 50 | 60.35 |
| nCoV-2019_63_LEFT | nCoV-2019_1 | TGTTAAGCGTGTTGACTGGACT | 22 | 45.45 | 60.16 |
| nCoV-2019_63_RIGHT | nCoV-2019_1 | ACAAACTGCCACCATCACAACC | 22 | 50 | 61.85 |
| nCoV-2019_64_LEFT | nCoV-2019_2 | TCGATAGATATCCTGCTAATTCCATTGT | 28 | 35.71 | 60.11 |
| nCoV-2019_64_RIGHT | nCoV-2019_2 | AGTCTTGTAAAAGTGTTCCAGAGGT | 25 | 40 | 60.1 |
| nCoV-2019_65_LEFT | nCoV-2019_1 | GCTGGCTTTAGCTTGTGGGTTT | 22 | 50 | 61.92 |
| nCoV-2019_65_RIGHT | nCoV-2019_1 | TGTCAGTCATAGAACAAACACCAATAGT | 28 | 35.71 | 60.9 |
| nCoV-2019_66_LEFT | nCoV-2019_2 | GGGTGTGGACATTGCTGCTAAT | 22 | 50 | 61.21 |
| nCoV-2019_66_RIGHT | nCoV-2019_2 | TCAATTTCCATTTGACTCCTGGGT | 24 | 41.67 | 60.45 |
| nCoV-2019_67_LEFT | nCoV-2019_1 | GTTGTCCAACAATTACCTGAAACTTACT | 28 | 35.71 | 60.43 |
| nCoV-2019_67_RIGHT | nCoV-2019_1 | CAACCTTAGAAACTACAGATAAATCTTGGG | 30 | 36.67 | 60.4 |
| nCoV-2019_68_LEFT | nCoV-2019_2 | ACAGGTTCATCTAAGTGTGTGTGT | 24 | 41.67 | 60.14 |
| nCoV-2019_68_RIGHT | nCoV-2019_2 | CTCCTTTATCAGAACCAGCACCA | 23 | 47.83 | 60.31 |
| nCoV-2019_69_LEFT | nCoV-2019_1 | TGTCGCAAAATATACTCAACTGTGTCA | 27 | 37.04 | 61.43 |
| nCoV-2019_69_RIGHT | nCoV-2019_1 | TCTTTATAGCCACGGAACCTCCA | 23 | 47.83 | 61.14 |
| nCoV-2019_70_LEFT | nCoV-2019_2 | ACAAAAGAAAATGACTCTAAAGAGGGTTT | 29 | 31.03 | 60.13 |

| nCoV-2019_70_RIGHT | nCoV-2019_2 | TGACCTTCTTTTAAAGACATAACAGCAG | 28 | 35.71 | 60.27 |
| --- | --- | --- | --- | --- | --- |
| nCoV-2019_71_LEFT | nCoV-2019_1 | ACAAATCCAATTCAGTTGTCTTCCTATTC | 29 | 34.48 | 60.54 |
| nCoV-2019_71_RIGHT | nCoV-2019_1 | TGGAAAAGAAAGGTAAGAACAAGTCCT | 27 | 37.04 | 60.8 |
| nCoV-2019_72_LEFT | nCoV-2019_2 | ACACGTGGTGTTTATTACCCTGAC | 24 | 45.83 | 61.04 |
| nCoV-2019_72_RIGHT | nCoV-2019_2 | ACTCTGAACTCACTTTCCATCCAAC | 25 | 44 | 60.97 |
| nCoV-2019_73_LEFT | nCoV-2019_1 | CAATTTTGTAATGATCCATTTTTGGGTGT | 29 | 31.03 | 60.29 |
| nCoV-2019_73_RIGHT | nCoV-2019_1 | CACCAGCTGTCCAACCTGAAGA | 22 | 54.55 | 62.45 |
| nCoV-2019_74_LEFT | nCoV-2019_2 | ACATCACTAGGTTTCAAACTTTACTTGC | 28 | 35.71 | 60.68 |
| nCoV-2019_74_RIGHT | nCoV-2019_2 | GCAACACAGTTGCTGATTCTCTTC | 24 | 45.83 | 60.85 |
| nCoV-2019_75_LEFT | nCoV-2019_1 | AGAGTCCAACCAACAGAATCTATTGT | 26 | 38.46 | 60.24 |
| nCoV-2019_75_RIGHT | nCoV-2019_1 | ACCACCAACCTTAGAATCAAGATTGT | 26 | 38.46 | 60.69 |
| nCoV-2019_76_LEFT | nCoV-2019_2 | AGGGCAAACTGGAAAGATTGCT | 22 | 45.45 | 60.76 |
| nCoV-2019_76_LEFT_alt3 | nCoV-2019_2 | GGGCAAACTGGAAAGATTGCTGA | 23 | 47.83 | 61.87 |
| nCoV-2019_76_RIGHT | nCoV-2019_2 | ACACCTGTGCCTGTTAAACCAT | 22 | 45.45 | 60.42 |
| nCoV-2019_76_RIGHT_alt0 | nCoV-2019_2 | ACCTGTGCCTGTTAAACCATTGA | 23 | 43.48 | 60.69 |
| nCoV-2019_77_LEFT | nCoV-2019_1 | CCAGCAACTGTTTGTGGACCTA | 22 | 50 | 60.75 |
| nCoV-2019_77_RIGHT | nCoV-2019_1 | CAGCCCCTATTAAACAGCCTGC | 22 | 54.55 | 61.59 |
| nCoV-2019_78_LEFT | nCoV-2019_2 | CAACTTACTCCTACTTGGCGTGT | 23 | 47.83 | 60.55 |
| nCoV-2019_78_RIGHT | nCoV-2019_2 | TGTGTACAAAAACTGCCATATTGCA | 25 | 36 | 60.22 |
| nCoV-2019_79_LEFT | nCoV-2019_1 | GTGGTGATTCAACTGAATGCAGC | 23 | 47.83 | 60.92 |
| nCoV-2019_79_RIGHT | nCoV-2019_1 | CATTTCATCTGTGAGCAAAGGTGG | 24 | 45.83 | 60.62 |
| nCoV-2019_80_LEFT | nCoV-2019_2 | TTGCCTTGGTGATATTGCTGCT | 22 | 45.45 | 60.89 |
| nCoV-2019_80_RIGHT | nCoV-2019_2 | TGGAGCTAAGTTGTTTAACAAGCG | 24 | 41.67 | 60.02 |
| nCoV-2019_81_LEFT | nCoV-2019_1 | GCACTTGGAAAACTTCAAGATGTGG | 25 | 44 | 61.24 |
| nCoV-2019_81_RIGHT | nCoV-2019_1 | GTGAAGTTCTTTTCTTGTGCAGGG | 24 | 45.83 | 60.73 |
| nCoV-2019_82_LEFT | nCoV-2019_2 | GGGCTATCATCTTATGTCCTTCCCT | 25 | 48 | 61.52 |
| nCoV-2019_82_RIGHT | nCoV-2019_2 | TGCCAGAGATGTCACCTAAATCAA | 24 | 41.67 | 60.02 |
| nCoV-2019_83_LEFT | nCoV-2019_1 | TCCTTTGCAACCTGAATTAGACTCA | 25 | 40 | 60.46 |
| nCoV-2019_83_RIGHT | nCoV-2019_1 | TTTGACTCCTTTGAGCACTGGC | 22 | 50 | 61.33 |
| nCoV-2019_84_LEFT | nCoV-2019_2 | TGCTGTAGTTGTCTCAAGGGCT | 22 | 50 | 61.61 |
| nCoV-2019_84_RIGHT | nCoV-2019_2 | AGGTGTGAGTAAACTGTTACAAACAAC | 27 | 37.04 | 60.36 |
| nCoV-2019_85_LEFT | nCoV-2019_1 | ACTAGCACTCTCCAAGGGTGTT | 22 | 50 | 61.03 |

| nCoV-2019_85_RIGHT | nCoV-2019_1 | ACACAGTCTTTTACTCCAGATTCCC | 25 | 44 | 60.51 |
| --- | --- | --- | --- | --- | --- |
| nCoV-2019_86_LEFT | nCoV-2019_2 | TCAGGTGATGGCACAACAAGTC | 22 | 50 | 61.07 |
| nCoV-2019_86_RIGHT | nCoV-2019_2 | ACGAAAGCAAGAAAAAGAAGTACGC | 25 | 40 | 61.01 |
| nCoV-2019_87_LEFT | nCoV-2019_1 | CGACTACTAGCGTGCCTTTGTA | 22 | 50 | 60.16 |
| nCoV-2019_87_RIGHT | nCoV-2019_1 | ACTAGGTTCCATTGTTCAAGGAGC | 24 | 45.83 | 60.81 |
| nCoV-2019_88_LEFT | nCoV-2019_2 | CCATGGCAGATTCCAACGGTAC | 22 | 54.55 | 61.58 |
| nCoV-2019_88_RIGHT | nCoV-2019_2 | TGGTCAGAATAGTGCCATGGAGT | 23 | 47.83 | 61.4 |
| nCoV-2019_89_LEFT | nCoV-2019_1 | GTACGCGTTCCATGTGGTCATT | 22 | 50 | 61.5 |
| nCoV-2019_89_LEFT_alt2 | nCoV-2019_1 | CGCGTTCCATGTGGTCATTCAA | 22 | 50 | 62.01 |
| nCoV-2019_89_RIGHT | nCoV-2019_1 | ACCTGAAAGTCAACGAGATGAAACA | 25 | 40 | 60.91 |
| nCoV-2019_89_RIGHT_alt4 | nCoV-2019_1 | ACGAGATGAAACATCTGTTGTCACT | 25 | 40 | 60.74 |
| nCoV-2019_90_LEFT | nCoV-2019_2 | ACACAGACCATTCCAGTAGCAGT | 23 | 47.83 | 61.58 |
| nCoV-2019_90_RIGHT | nCoV-2019_2 | TGAAATGGTGAATTGCCCTCGT | 22 | 45.45 | 60.82 |
| nCoV-2019_91_LEFT | nCoV-2019_1 | TCACTACCAAGAGTGTGTTAGAGGT | 25 | 44 | 60.93 |
| nCoV-2019_91_RIGHT | nCoV-2019_1 | TTCAAGTGAGAACCAAAAGATAATAAGCA | 29 | 31.03 | 60.03 |
| nCoV-2019_92_LEFT | nCoV-2019_2 | TTTGTGCTTTTTAGCCTTTCTGCT | 24 | 37.5 | 60.14 |
| nCoV-2019_92_RIGHT | nCoV-2019_2 | AGGTTCCTGGCAATTAATTGTAAAAGG | 27 | 37.04 | 60.53 |
| nCoV-2019_93_LEFT | nCoV-2019_1 | TGAGGCTGGTTCTAAATCACCCA | 23 | 47.83 | 61.59 |
| nCoV-2019_93_RIGHT | nCoV-2019_1 | AGGTCTTCCTTGCCATGTTGAG | 22 | 50 | 60.55 |
| nCoV-2019_94_LEFT | nCoV-2019_2 | GGCCCCAAGGTTTACCCAATAA | 22 | 50 | 60.56 |
| nCoV-2019_94_RIGHT | nCoV-2019_2 | TTTGGCAATGTTGTTCCTTGAGG | 23 | 43.48 | 60.18 |
| nCoV-2019_95_LEFT | nCoV-2019_1 | TGAGGGAGCCTTGAATACACCA | 22 | 50 | 61.1 |
| nCoV-2019_95_RIGHT | nCoV-2019_1 | CAGTACGTTTTTGCCGAGGCTT | 22 | 50 | 61.95 |
| nCoV-2019_96_LEFT | nCoV-2019_2 | GCCAACAACAACAAGGCCAAAC | 22 | 50 | 61.82 |
| nCoV-2019_96_RIGHT | nCoV-2019_2 | TAGGCTCTGTTGGTGGGAATGT | 22 | 50 | 61.36 |
| nCoV-2019_97_LEFT | nCoV-2019_1 | TGGATGACAAAGATCCAAATTTCAAAGA | 28 | 32.14 | 60.22 |
| nCoV-2019_97_RIGHT | nCoV-2019_1 | ACACACTGATTAAAGATTGCTATGTGAG | 28 | 35.71 | 60.17 |
| nCoV-2019_98_LEFT | nCoV-2019_2 | AACAATTGCAACAATCCATGAGCA | 24 | 37.5 | 60.5 |
| nCoV-2019_98_RIGHT | nCoV-2019_2 | TTCTCCTAAGAAGCTATTAAAATCACATGG | 30 | 33.33 | 60.01 |
