# Supplementary material for: Acute SARS-CoV-2 infections harbor limited within-host diversity and transmit via tight transmission bottlenecks
Source: PLoS Pathog. 2021 Aug 23;17(8):e1009849. doi: 10.1371/journal.ppat.1009849 (PMC8412271; doi:10.1371/journal.ppat.1009849)
Supplement: S4 Table — (DOCX) [file ppat.1009849.s016.docx]

| C Column 3 | Column 4 Column 5 | Column 6 | Column 7 | Column 8 | Column 9 | Column 10 | Column 11 | Column 12 | Column 13 | Column 14 |
| --- | --- | --- | --- | --- | --- | --- | --- | --- | --- | --- |
| 0 tip1 | tip2 muts_between_tips | muts | probability_1_serial_inteval | tube_IDs | time_between_test | direction_based_on_test_date | time_between_symptoms | direction_based_on_symptoms | #comparisons | pair_number |
| 0 USA/WI-UW-41/2020 | USA/WI-UW-48/2020 0 | [] | 0.7496528051576963 | 20,28 | 0 | 28 <--> 20 | 1 | 28 <--> 20 | 2 | pair1, pair1a (28 --> 20), pair 1b (20 --> 28) |
| 0 USA/WI-UW-65/2020 | USA/WI-UW-32/2020 0 | [] | 0.7496528051576963 | 50,8 | 2 | 8 <--> 50 | 2 | 8 <--> 50 | 2 | pair2, pair2a (8 --> 50), pair2b (50 --> 8) |
| 0 USA/WI-UW-69/2020 | USA/WI-UW-61/2020 0 | [] | 0.7496528051576963 | 55,44 | 4 | 55 --> 44 | 3 | 55 <--> 44 | 2 | pair3, pair3a (55 --> 44), pair3b (44 --> 55) |
| 0 USA/WI-UW-70/2020 | USA/WI-UW-67/2020 0 | [] | 0.7496528051576963 | 56,53 | 6 | 56 --> 53 | 5 | 56 --> 53 | 1 | pair4 |
| 0 USA/WI-UW-74/2020 | USA/WI-UW-29/2020 0 | [] | 0.7496528051576963 | 61,5 | 4 | 61 --> 5 | 4 | 61 --> 5 | 1 | pair5 |
| 0 USA/WI-UW-438/202 | USA/WI-UW-432/202 0 | [] | 0.7496528051576963 | 744,738 | 2 | 738 --> 744 | 2 | 738 --> 744 | 2 | pair6, pair 6a (738 --> 744), pair 6b (744 --> 738) |
| 0 USA/WI-UW-544/202 | USA/WI-UW-551/202 1 | ['T4917C'] | 0.21600878707411836 | 893,884 | 1 | 893 <--> 884 | 4 | 884 --> 893 | 1 | pair7 |
| 0 USA/WI-UW-544/202 | USA/WI-UW-575/202 0 | [] | 0.7496528051576963 | 884,903 | 1 | 884 <--> 903 | asx | 884 <--> 903 | 2 | pair8, pair8a (884 --> 903), pair8b (903 --> 884) |
| 0 USA/WI-UW-551/202 | USA/WI-UW-575/202 1 | ['T4917C'] | 0.21600878707411836 | 893,903 | 0 | 893 <--> 903 | asx | 893 <--> 903 | 2 | pair9, pair9a (893 --> 903), pair9b (903 --> 893) |
| 0 USA/WI-UW-546/202 | USA/WI-UW-586/202 0 | [] | 0.7496528051576963 | 887,916 | 0 | 887 <--> 916 | 1 | 887 <--> 916 | 2 | pair10, pair10a (887 --> 916), pair 10b (916 --> 887) |
| 0 USA/WI-UW-546/202 | USA/WI-UW-443/202 0 | [] | 0.7496528051576963 | 887,749 | 0 | 887 <--> 749 | 0 | 887 <--> 749 | 2 | pair11, pair 11a (887 --> 749), pair 11b (749 --> 887) |
| 0 USA/WI-UW-586/202 | USA/WI-UW-443/202 0 | [] | 0.7496528051576963 | 916,749 | 0 | 916 <--> 749 | 1 | 916 <--> 749 | 2 | pair12, pair12a (916 --> 749), pair12b (749 --> 916) |
| 0 USA/WI-UW-577/202 | USA/WI-UW-536/202 0 | [] | 0.7496528051576963 | 906,849 | 0 | 906 <--> 849 | asx | 906 <--> 849 | 2 | pair13, pair13a (906 --> 849), piar13b (849 --> 906) |
| 0 USA/WI-UW-598/202 | USA/WI-UW-602/202 0 | [] | 0.7496528051576963 | 956,962 | 3 | 956 --> 962 | 4 | 962 --> 956 | 2 | pair14 |
| 0 USA/WI-UW-601/202 USA/WI-UW-780/202 0 [] | | | 0.7496528051576963 | 961,1195 | 8 | 961 --> 1195 | 5 | 961 --> 1195 | 1 | pair15 |
| ? USA/WI-UW-756/202 USA/WI-UW-893/202 0 [] | | | 0.7496528051576963 | 1157,1346 | 7 | 1157 --> 1346 | 6 | 1157 --> 1346 | 1 | pair16 |
| 0 USA/WI-UW-874/202 USA/WI-UW-986/202 0 [] | | | 0.7496528051576963 | 1326,1495 | 3 | 1326 --> 1495 | asx | 1326 <--> 1495 | 1 | pair17 |
| 0 USA/WI-UW-874/202 USA/WI-UW-997/202 0 [] | | | 0.7496528051576963 | 1326,1512 | 3 | 1326 --> 1512 | asx | 1326 <--> 1512 | 1 | pair18 |
| 0 USA/WI-UW-874/202 USA/WI-UW-991/202 0 [] | | | 0.7496528051576963 | 1326,1502 | 3 | 1326 --> 1502 | asx | 1326 <--> 1502 | 1 | pair19 |
| 0 USA/WI-UW-986/202 USA/WI-UW-997/202 0 [] | | | 0.7496528051576963 | 1495,1512 | 0 | 1495 <--> 1512 | asx | 1495 <--> 1512 | 2 | pair20, pair20a (1495 --> 1512), pair20b (1512 --> 1495) |
| 0 USA/WI-UW-986/202 USA/WI-UW-991/202 0 [] | | | 0.7496528051576963 | 1495,1502 | 0 | 1495 <--> 1502 | asx | 1495 <--> 1502 | 2 | pair21, pair21a (1495 --> 1502), pair21b (1502 --> 1495) |
| 0 USA/WI-UW-997/202 USA/WI-UW-991/202 0 [] | | | 0.7496528051576963 | 1512,1502 | 0 | 1512 <--> 1502 | asx | 1512 <--> 1502 | 2 | pair22, pair22a (1512 --> 1502), pair22b (1502 --> 1512) |
| 0 USA/WI-UW-895/202 USA/WI-UW-876/202 0 [] | | | 0.7496528051576963 | 1353,1328 | 0 | 1353 <--> 1328 | 3 | 1353 --> 1328 | 1 | pair23 |
| 0 USA/WI-UW-895/202 USA/WI-UW-863/202 2 ['A15942C', 'C25006T'] | | | 0.031120937434107567 | 1353,1297 | 0 | 1353 <--> 1297 | 3 | 1353 --> 1297 | 1 | pair24 |
| 0 USA/WI-UW-876/202 USA/WI-UW-863/202 2 ['A15942C', 'C25006T'] | | | 0.031120937434107567 | 1328,1297 | 0 | 1297 <--> 1328 | 0 | 1297 <--> 1328 | 1 | pair25, pair25a (1297 --> 1328), pair25b (1328 --> 1297) |
| 0 USA/WI-UW-158/202 USA/WI-UW-160/202 0 [] | | | 0.7496528051576963 | 195,197 | 0 | 195 <--> 197 | NA | 195 <--> 197 | 2 | pair26, pair26a (195 --> 197), pair26b (197 --> 195) |
| 0 USA/WI-UW-333/202 USA/WI-UW-334/202 0 [] | | | 0.7496528051576963 | 453,454 | 0 | 453 <--> 454 | NA | 453 <--> 454 | 2 | pair27, pair27a (453 --> 454), pair27b (454 --> 453) |
| 0 USA/WI-UW-119/202 USA/WI-UW-120/202 0 [] | | | 0.7496528051576963 | 128,130 | 3 | 128 --> 130 | 10 | 130 --> 128 | 1 | pair28 |
