# Supplementary material for: Acute SARS-CoV-2 infections harbor limited within-host diversity and transmit via tight transmission bottlenecks
Source: PLoS Pathog. 2021 Aug 23;17(8):e1009849. doi: 10.1371/journal.ppat.1009849 (PMC8412271; doi:10.1371/journal.ppat.1009849)
Supplement: S5 Table — (PDF) [file ppat.1009849.s017.pdf]

Supplemental Table 5. List of all iSNVs and their respective frequencies.

protein	nucleotide change	amino acid change	SNP frequency in all samples
E	103A>G	Thr35Ala	[0.0376]
E	13G>A	Val5Ile	[1.0]
E	153T>C	Leu51Leu	[0.0956]
E	187A>T	Lys63*	[0.0501]
E	224T>C	Val75Ala	[0.0427]
E	80T>C	Leu27Ser	[0.0806]
E	81G>C	Leu27Phe	[0.0569]
E	96C>T	Ala32Ala	[0.0345]
E	98T>C	Ile33Thr	[0.0635]
M	112G>T	Ala38Ser	[1.0]
M	159C>T	Phe53Phe	[0.0489]
M	170T>C	Leu57Ser	[0.0351]
M	240T>C	Ile80Ile	[0.0572]
M	247G>T	Ala83Ser	[0.0368]
M	300C>T	Phe100Phe	[0.0345]
M	406A>G	Ser136Gly	[0.0446]
M	429G>T	Val143Val	[0.0533]
M	459A>G	Gly153Gly	[0.12745]
M	495T>A	Pro165Pro	[0.0589]
M	509T>C	Val170Ala	[0.0306]
M	524C>T	Thr175Met	[0.0562]
M	592C>T	Arg198Cys	[0.0312]
N	1011C>A	Ile337Ile	[0.04]
N	104C>T	Ala35Val	[0.0308]
N	1074C>T	As58Asp	[1.0]
N	1080C>T	Tyr360Tyr	[0.0319]
N	1081A>C	Lys361Gln	[0.0789]
N	1082A>T	Lys361Ile	[0.0789]
N	1129G>T	As77Tyr	[0.21025]

N	1132G>A	Glu378Lys	[0.0733]
N	1136C>T	Thr379Ile	[0.0479]
N	1160A>G	Lys387Arg	[0.284]
N	1186C>T	Pro396Ser	[0.0398]
N	1193C>T	Ala398Val	[0.04675]
N	119G>A	Arg40His	[0.0542]
N	1206T>C	As02Asp	[0.0476]
N	1247C>T	Ser416Leu	[0.0328]
N	254G>T	Gly85Val	[0.052000000000000005]
N	282T>G	Ile94Met	[0.045]
N	284G>T	Arg95Leu	[0.0457]
N	339A>G	Leu113Leu	[0.0337]
N	400G>C	Ala134Pro	[0.05]
N	404C>T	Thr135Ile	[1.0]
N	451C>T	Pro151Ser	[0.25670000000000004]
N	48G>T	Thr16Thr	[0.0594]
N	519A>T	Ala173Ala	[0.0484]
N	540T>C	Ser180Ser	[0.0317]
N	548C>A	Ser183Tyr	[1.0, 1.0, 1.0, 0.95565, 0.9204, 1.0, 0.9665]
N	560C>T	Ser187Leu	[0.034, 0.0571]
N	576C>T	Asn192Asn	[0.0303]
N	581C>T	Ser194Leu	[0.9277, 1.0]
N	593C>T	Thr198Ile	[0.0453]
N	598G>A	Gly200Ser	[1.0, 1.0]
N	605G>A	Ser202Asn	[1.0, 0.9667, 1.0, 1.0, 1.0, 1.0, 1.0, 1.0, 1.0, 1.0, 1.0, 1.0, 1.0, 1.0, 1.0, 1.0, 1.0, 1.0, 1.0]
N	608G>A	Arg203Lys	[1.0, 0.9686, 1.0]
N	608G>T	Arg203Met	[0.1083]
N	609G>A	Arg203Arg	[1.0, 0.9681, 1.0]
N	609G>T	Arg203Ser	[0.1083]
N	610G>C	Gly204Arg	[1.0, 0.9686, 1.0]
N	610G>T	Gly204*	[0.1083]

N	612A>T	Gly204Gly	[0.1083]
N	613A>G	Thr205Ala	[0.1083]
N	614C>T	Thr205Ile	[0.0515]
N	617C>T	Ser206Phe	[0.1083]
N	619C>T	Pro207Ser	[0.1083]
N	626G>A	Arg209Lys	[0.1432]
N	626G>T	Arg209Ile	[0.1083]
N	627A>C	Arg209Ser	[0.1432]
N	627A>G	Arg209Arg	[0.0397]
N	627A>T	Arg209Ser	[0.1083]
N	628A>C	Met210Leu	[0.1432]
N	629T>C	Met210Thr	[0.1432]
N	630G>A	Met210Ile	[0.1432]
N	631G>A	Ala211Thr	[0.1432]
N	632C>T	Ala211Val	[0.1432]
N	633T>A	Ala211Ala	[0.1434]
N	634G>A	Gly212Ser	[0.1448]
N	635G>T	Gly212Val	[0.1434]
N	636C>A	Gly212Gly	[0.1434]
N	637A>C	Asn213His	[0.1434]
N	638A>T	Asn213Ile	[0.1436]
N	639T>G	Asn213Lys	[0.1436]
N	646G>T	As16Tyr	[0.08485000000000001]
N	649G>A	Ala217Thr	[0.0301]
N	657T>C	Leu219Leu	[0.96295]
N	662T>C	Leu221Ser	[0.0302]
N	702G>T	Met234Ile	[0.0699]
N	703T>A	Ser235Thr	[0.1364, 0.045]
N	703T>G	Ser235Ala	[0.0423]
N	704C>T	Ser235Phe	[0.045, 1.0, 1.0, 0.96965, 1.0, 1.0, 0.9646, 1.0, 1.0, 0.91135, 1.0, 0.96635, 0.9642]
N	705T>A	Ser235Ser	[0.1364, 0.0305]

N	706G>A	Gly236Ser	[0.1364]
N	706G>C	Gly236Arg	[0.0493]
N	707G>A	Gly236Asp	[0.0423]
N	707G>C	Gly236Ala	[0.1364]
N	708T>C	Gly236Gly	[0.0563]
N	708T>G	Gly236Gly	[0.1364]
N	709A>T	Lys237*	[0.1364]
N	710A>C	Lys237Thr	[0.1364]
N	710A>G	Lys237Arg	[0.0426]
N	711A>G	Lys237Lys	[0.1364]
N	712G>C	Gly238Arg	[0.0305]
N	735T>C	Thr245Thr	[0.1364]
N	736G>T	Val246Phe	[0.1364]
N	737T>G	Val246Gly	[0.1364]
N	804C>T	Tyr268Tyr	[0.06565, 0.4574]
N	851G>A	Gly284Glu	[0.0328]
N	855T>A	Asn285Lys	[0.0302, 0.0992]
N	861G>C	Gly287Gly	[0.1591]
N	898C>T	His300Tyr	[0.0508]
N	916C>T	Gln306*	[0.2959]
N	933T>C	Ala311Ala	[1.0]
N	944T>C	Phe315Ser	[0.077]
N	954G>T	Ser318Ser	[0.04905]
N	955C>T	Arg319Cys	[0.0408]
N	956G>A	Arg319His	[0.06815]
N	960T>C	Ile320Ile	[0.0805]
N	974C>T	Thr325Ile	[0.05865]
N	981G>T	Ser327Ser	[1.0]
N	996C>T	Thr332Thr	[1.0]
ORF10	102C>T	Asn34Asn	[0.0342]
ORF10	57C>T	Cys19Cys	[1.0, 1.0]

ORF10	5G>T	Gly2Val	[0.0448]
ORF10	70C>T	Arg24Cys	[0.043300000000000005, 0.121, 1.0, 1.0, 1.0]
ORF10	75C>G	Asn25Lys	[0.0705]
ORF10	85C>T	Gln29*	[0.0596]
ORF3a	108G>T	Pro36Pro	[1.0, 0.9609, 0.96325, 1.0, 1.0, 1.0, 1.0, 0.95735, 1.0, 1.0, 0.9673, 0.96645, 1.0, 1.0, 1.0, 1.0, 1.0, 0.96575, 0.9399]
ORF3a	120A>C	Ser40Ser	[0.0328]
ORF3a	146G>A	Gly49Asp	[0.0423]
ORF3a	148G>T	Val50Phe	[0.0436]
ORF3a	171G>T	Gln57His	[1.0, 1.0, 1.0, 1.0, 1.0, 1.0, 1.0, 1.0, 1.0, 1.0, 1.0, 1.0, 1.0, 1.0, 1.0, 1.0, 1.0, 1.0, 1.0, 0.9655, 1.0, 1.0, 1.0, 1.0, 0.9287, 0.0574, 0.93475, 1.0, 1.0, 1.0, 1.0, 1.0, 1.0, 1.0, 0.9617, 1.0, 1.0, 0.96885, 0.0388, 1.0, 1.0, 1.0, 1.0, 1.0, 1.0, 1.0, 1.0, 1.0, 1.0, 1.0, 1.0, 1.0, 0.9662, 1.0, 1.0]
ORF3a	195C>T	Leu65Leu	[0.0659]
ORF3a	232C>T	His78Tyr	[0.0301]
ORF3a	26C>T	Thr9Ile	[0.0425]
ORF3a	288C>T	Leu96Leu	[0.0312]
ORF3a	295G>A	Ala99Thr	[1.0]
ORF3a	317T>G	Leu106Arg	[0.066]
ORF3a	31G>C	Gly11Arg	[0.2108]
ORF3a	377G>A	Arg126Lys	[0.0365]
ORF3a	385C>T	Leu129Phe	[0.0304, 0.0365]
ORF3a	418C>T	Leu140Phe	[0.0543]
ORF3a	48G>T	Lys16Asn	[1.0, 0.96665, 1.0, 1.0]
ORF3a	49C>T	Gln17*	[0.0607]
ORF3a	60C>T	Ile20Ile	[0.06220000000000001]
ORF3a	650C>T	Thr217Ile	[0.0317]
ORF3a	732C>T	Val244Val	[1.0, 1.0, 1.0, 0.96495, 1.0, 1.0, 1.0]
ORF3a	760G>A	Gly254Arg	[0.0638]
ORF3a	784C>T	Pro262Ser	[0.3202]
ORF3a	800C>T	Pro267Leu	[1.0]
ORF3a	85G>T	Val29Phe	[0.0393]
ORF3a	88C>T	Arg30Cys	[0.039]
ORF6	102C>G	Asn34Lys	[0.0379]

ORF6	103C>A	Leu35Ile	[0.0379]
ORF6	111T>G	Ile37Met	[0.0379]
ORF6	115A>C	Asn39His	[0.0379]
ORF6	116A>T	Asn39Ile	[0.0379]
ORF6	121T>A	Ser41Thr	[0.0565]
ORF6	122C>T	Ser41Phe	[0.0565]
ORF6	123T>C	Ser41Ser	[0.0565]
ORF6	124A>C	Lys42Gln	[0.0565]
ORF6	127T>A	Ser43Thr	[0.0565]
ORF6	128C>A	Ser43*	[0.0565]
ORF6	137A>G	Glu46Gly	[1.0, 1.0]
ORF6	147T>C	Tyr49Tyr	[1.0, 1.0]
ORF6	169C>T	Pro57Ser	[0.0553]
ORF6	182A>C	As1Ala	[0.0381]
ORF6	183T>C	As1Asp	[0.0381]
ORF6	60G>A	Arg20Arg	[0.0507]
ORF6	74C>A	Ser25Tyr	[0.038]
ORF6	75C>T	Ser25Ser	[0.0382]
ORF6	76A>G	Ile26Val	[0.0382]
ORF6	77T>C	Ile26Thr	[0.038]
ORF6	80G>A	Tr7*	[0.038]
ORF6	81G>T	Tr7Cys	[0.0381]
ORF6	82A>T	Asn28Tyr	[0.0381]
ORF6	88G>T	As0Tyr	[0.0379]
ORF6	89A>T	As0Val	[0.038]
ORF6	90T>G	As0Glu	[0.0379]
ORF6	91T>G	Tyr31Asp	[0.0379]
ORF6	92A>T	Tyr31Phe	[0.0379]
ORF6	93C>T	Tyr31Tyr	[0.0304, 0.0379]
ORF6	94A>C	Ile32Leu	[0.0379]
ORF6	98T>G	Ile33Arg	[0.0379]

ORF6	99A>C	Ile33Ile	[0.0379]
ORF7a	112G>C	Gly38Arg	[0.04925]
ORF7a	184C>T	Gln62*	[0.95675, 1.0]
ORF7a	189T>C	Phe63Phe	[0.036500000000000005]
ORF7a	219C>T	His73His	[0.0555]
ORF7a	232C>A	Arg78Ser	[0.0447]
ORF7a	233G>T	Arg78Leu	[0.0447]
ORF7a	25C>T	Leu9Leu	[0.06265]
ORF7a	269A>G	Gln90Arg	[0.1656]
ORF7a	291C>A	Tyr97*	[0.1003]
ORF7a	292T>A	Ser98Thr	[0.1003]
ORF7a	294T>A	Ser98Ser	[0.0998]
ORF7a	296C>A	Pro99Gln	[0.1003]
ORF7a	297A>G	Pro99Pro	[0.1007]
ORF7a	299T>C	Ile100Thr	[0.1003]
ORF7a	300T>C	Ile100Ile	[0.1003]
ORF7a	301T>A	Phe101Ile	[0.1003]
ORF7a	305T>C	Leu102Pro	[0.1003]
ORF7a	306T>A	Leu102Leu	[0.1003]
ORF7a	307A>G	Ile103Val	[0.1003]
ORF7a	309T>A	Ile103Ile	[0.1012]
ORF7a	311T>C	Val104Ala	[0.1003]
ORF7a	312T>A	Val104Val	[0.1017]
ORF7a	314C>T	Ala105Val	[0.1003]
ORF7a	316G>A	Ala106Thr	[0.1003]
ORF7a	322G>T	Val108Leu	[0.1012]
ORF8	113C>T	Pro38Leu	[0.0309]
ORF8	178T>C	Leu60Leu	[0.035500000000000004]
ORF8	183C>A	Cys61*	[0.0516]
ORF8	251T>C	Leu84Ser	[1.0, 1.0, 1.0, 1.0, 1.0, 1.0, 1.0, 1.0, 1.0, 1.0, 1.0, 1.0, 1.0, 1.0, 1.0, 1.0, 1.0, 1.0, 1.0, 1.0, 1.0]
ORF8	321C>T	As07Asp	[0.0383, 0.0914]

ORF8	32C>T	Thr11Ile	[0.0542]
ORF8	345T>A	Arg115Arg	[0.048]
ORF8	346G>T	Val116Phe	[0.048]
ORF8	347T>C	Val116Ala	[0.048]
ORF8	349G>C	Val117Leu	[0.048]
ORF8	352T>G	Leu118Val	[0.048]
ORF8	358T>G	Phe120Val	[0.0534]
ORF8	360C>T	Phe120Phe	[0.0534, 0.036500000000000005]
ORF8	361A>C	Ile121Leu	[0.0534]
ORF8	54A>C	Gln18His	[0.0396]
ORF8	71C>T	Ser24Leu	[1.0, 0.0417]
ORF8	77C>T	Thr26Ile	[0.06885000000000001]
S	1035C>T	Thr345Thr	[0.0468]
S	1100T>C	Val367Ala	[0.0716]
S	1114G>T	Ala372Ser	[0.0863]
S	1129T>C	Phe377Leu	[0.1417]
S	1136G>T	Cys379Phe	[0.0447]
S	1173C>T	Cys391Cys	[0.031]
S	122A>G	Lys41Arg	[0.042]
S	1282G>T	As28Tyr	[0.109]
S	1342A>T	Asn448Tyr	[0.0851]
S	1343A>T	Asn448Ile	[0.0856]
S	1346A>T	Tyr449Phe	[0.0856]
S	1347T>C	Tyr449Tyr	[0.0846]
S	1354C>A	Leu452Met	[0.0856]
S	1355T>C	Leu452Pro	[0.0856]
S	1358A>G	Tyr453Cys	[0.0856]
S	1359T>C	Tyr453Tyr	[0.0856]
S	1362A>G	Arg454Arg	[0.0856]
S	1363T>C	Leu455Leu	[0.0861]
S	1372A>G	Lys458Glu	[0.0861]

S	1373A>C	Lys458Thr	[0.0856]
S	1374G>T	Lys458Asn	[0.0861]
S	1375T>G	Ser459Ala	[0.0861]
S	1376C>A	Ser459Tyr	[0.0851]
S	1377T>A	Ser459Ser	[0.0856]
S	1378A>C	Asn460His	[0.0856]
S	1381C>G	Leu461Val	[0.0856]
S	1388C>T	Pro463Leu	[0.0376]
S	13C>T	Leu5Phe	[0.0487, 0.0519]
S	1411G>A	Glu471Lys	[0.0763]
S	1411G>C	Glu471Gln	[0.0861]
S	1413A>T	Glu471Asp	[0.0856]
S	1416C>G	Ile472Met	[0.0856]
S	1417T>A	Tyr473Asn	[0.0855]
S	1418A>G	Tyr473Cys	[0.0855]
S	1423G>T	Ala475Ser	[0.0855]
S	1424C>G	Ala475Gly	[0.0855]
S	1425C>A	Ala475Ala	[0.0865]
S	1425C>T	Ala475Ala	[0.0324, 0.086]
S	1426G>C	Gly476Arg	[0.086]
S	1427G>A	Gly476Asp	[0.086]
S	145C>T	His49Tyr	[1.0]
S	1525A>G	Arg509Gly	[0.0353]
S	1617C>A	Val539Val	[0.0905]
S	1626C>T	Asn542Asn	[0.1939]
S	1686C>T	Phe562Phe	[0.0382]
S	1690C>T	Gln564*	[0.0668]
S	1694T>C	Phe565Ser	[0.0324]
S	1716T>C	Thr572Thr	[0.046]
S	1739A>G	Gln580Arg	[0.0441]
S	1800A>G	Pro600Pro	[0.0319]

S	1831C>T	Leu611Phe	[0.031]
S	1841A>G	As14Gly	[1.0, 1.0, 1.0, 1.0, 1.0, 0.96265, 1.0, 1.0, 1.0, 1.0, 1.0, 1.0, 1.0, 1.0, 1.0, 1.0, 1.0, 1.0, 1.0, 1.0, 0.969, 1.0, 1.0, 1.0, 1.0, 1.0, 1.0, 0.96245, 1.0, 1.0, 0.9462, 1.0, 1.0, 1.0, 0.9295, 0.9343, 1.0, 1.0, 1.0, 1.0, 1.0, 1.0, 1.0, 0.9546, 1.0, 1.0, 0.9656, 1.0, 1.0, 1.0, 1.0, 1.0, 1.0, 1.0, 1.0, 1.0, 1.0, 1.0, 1.0, 1.0, 1.0, 1.0, 1.0, 1.0, 1.0, 1.0, 1.0, 1.0, 1.0, 1.0, 1.0, 1.0, 1.0, 1.0, 1.0, 1.0, 1.0, 1.0, 1.0, 1.0, 1.0]
S	1848C>T	Asn616Asn	[0.0356]
S	1966G>T	Val656Phe	[0.0507]
S	1975T>C	Ser659Pro	[0.0389]
S	1989C>A	As63Glu	[0.0391]
S	1992A>T	Ile664Ile	[0.0391, 0.0374]
S	199G>A	Ala67Thr	[0.0315]
S	2007T>C	Gly669Gly	[0.0508]
S	2013C>T	Cys671Cys	[0.0549]
S	2047C>T	Arg683Trp	[0.0337]
S	2072C>T	Ser691Phe	[0.0394]
S	2116G>T	Ala706Ser	[0.0389]
S	2117C>T	Ala706Val	[0.0328]
S	2119T>C	Tyr707His	[0.0351]
S	2130C>T	Asn710Asn	[0.0402]
S	2145C>T	Pro715Pro	[1.0, 1.0]
S	214G>A	Gly72Arg	[0.0697]
S	2183C>T	Pro728Leu	[0.0389]
S	2196C>T	Thr732Thr	[0.50105]
S	219C>A	Thr73Thr	[0.0455]
S	2204C>T	Ser735Leu	[0.0402]
S	224G>T	Gly75Val	[0.0313]
S	228T>C	Thr76Thr	[0.0303]
S	2297C>T	Ala766Val	[0.0343]
S	2317G>A	Glu773Lys	[0.0584]
S	2362A>T	Ile788Phe	[0.0308]
S	2385A>G	Lys795Lys	[1.0, 1.0, 1.0, 1.0, 0.9426, 1.0, 1.0, 1.0, 1.0]
S	2387A>T	As96Val	[0.1578]
S	2392G>T	Gly798Cys	[0.0558]

S	2412A>C	Gln804His	[0.0465]
S	2424T>C	As08Asp	[0.1247]
S	2434C>T	Pro812Ser	[0.0564, 0.0302]
S	2472C>T	Asn824Asn	[0.0361]
S	2484T>C	Leu828Leu	[0.0413]
S	2506C>T	Gln836*	[0.0355]
S	25C>T	Pro9Ser	[0.0706]
S	2619C>T	Tyr873Tyr	[0.0458]
S	2651C>G	Ser884Cys	[0.56755]
S	2661C>T	Thr887Thr	[0.0356]
S	267T>G	Gly89Gly	[0.0307]
S	268G>T	Val90Phe	[0.0307]
S	2691A>T	Pro897Pro	[1.0, 1.0, 1.0]
S	2713A>G	Arg905Gly	[0.0319]
S	2755A>G	Asn919Asp	[0.0456]
S	2766G>A	Leu922Leu	[0.0398]
S	2770G>T	Ala924Ser	[0.0425]
S	2786G>T	Ser929Ile	[0.0757]
S	2789C>T	Ala930Val	[0.0414]
S	2796C>A	Gly932Gly	[0.0351, 0.0318]
S	27A>G	Pro9Pro	[0.0398]
S	2813T>C	Leu938Pro	[0.0389, 0.2029]
S	2824G>T	Ala942Ser	[0.1164]
S	2855T>C	Val952Ala	[0.0352]
S	2949T>C	Arg983Arg	[0.0343]
S	3066T>A	Ala1022Ala	[0.0496]
S	3076G>T	Ala1026Ser	[0.0475]
S	3104G>T	Gly1035Val	[0.0682]
S	3113A>C	Lys1038Thr	[0.0306]
S	3131G>A	Gly1044Glu	[0.0303]
S	3185T>C	Phe1062Ser	[0.0524]

S	3201T>C	Tyr1067Tyr	[0.0347]
S	3296G>A	Gly1099Asp	[0.032]
S	3300A>G	Thr1100Thr	[1.0]
S	3301C>T	His1101Tyr	[0.0448]
S	3304T>C	Tr102Arg	[0.0315]
S	3334C>G	Pro1112Ala	[0.0357]
S	3342C>T	Ile1114Ile	[0.0423]
S	335C>T	Ser112Leu	[0.08185]
S	3363T>C	Phe1121Phe	[0.033]
S	3371G>T	Gly1124Val	[0.0325, 0.0516, 0.1575]
S	3376T>C	Cys1126Arg	[0.0368]
S	3390A>G	Ile1130Met	[0.0384]
S	3444C>T	Phe1148Phe	[1.0, 0.3715, 0.8301000000000001]
S	3544G>C	Glu1182Gln	[0.2581]
S	3546A>T	Glu1182Asp	[0.0316]
S	3598C>T	Leu1200Phe	[0.0462]
S	3602A>C	Gln1201Pro	[0.0929]
S	3614A>T	Lys1205Met	[0.0308]
S	390C>T	Val130Val	[0.22575, 0.0365]
S	399T>C	Phe133Phe	[0.0486]
S	39T>C	Ser13Ser	[0.0323]
S	502T>C	Phe168Leu	[0.0307]
S	522T>C	Pro174Pro	[0.0649, 0.0564]
S	542G>A	Gly181Glu	[0.0441]
S	543A>G	Gly181Gly	[0.035]
S	555T>C	Asn185Asn	[0.0542]
S	570G>A	Arg190Arg	[0.0303]
S	579G>T	Val193Val	[0.0705]
S	593A>G	As98Gly	[0.0315]
S	601T>C	Phe201Leu	[0.0422]
S	621C>T	His207His	[0.0516]

S	663G>T	Ser221Ser	[1.0]
S	664G>T	Ala222Ser	[0.0471]
S	683A>T	As28Val	[0.0471]
S	688C>T	Pro230Ser	[0.2777]
S	72A>C	Leu24Phe	[0.0307]
S	76C>G	Pro26Ala	[0.042]
S	77C>A	Pro26His	[0.042]
S	7G>T	Val3Phe	[0.0424]
S	80C>A	Ala27Glu	[0.042]
S	80C>T	Ala27Val	[0.0493]
S	81A>T	Ala27Ala	[0.042]
S	832A>T	Lys278*	[0.0515]
S	83A>T	Tyr28Phe	[0.0444]
S	84C>T	Tyr28Tyr	[0.042]
S	857C>T	Thr286Ile	[0.0343]
S	859G>T	As87Tyr	[0.0315]
S	88A>G	Asn30Asp	[0.042]
S	890C>T	Ser297Leu	[0.0323]
S	922G>T	Val308Leu	[0.0348]
S	924A>C	Val308Val	[0.0376]
S	925G>A	Glu309Lys	[0.11199999999999999, 0.0376]
S	927A>T	Glu309Asp	[0.11199999999999999, 0.0484]
S	928A>T	Lys310*	[0.11199999999999999, 0.0376]
S	929A>T	Lys310Ile	[0.11199999999999999, 0.0376]
S	930A>T	Lys310Asn	[0.11199999999999999, 0.0484]
S	932G>T	Gly311Val	[0.11199999999999999, 0.0376]
S	936C>G	Ile312Met	[0.11199999999999999, 0.0376]
S	937T>A	Tyr313Asn	[0.11199999999999999, 0.0376]
S	938A>T	Tyr313Phe	[0.11199999999999999, 0.0376]
S	939T>C	Tyr313Tyr	[0.11199999999999999, 0.0376]
S	942A>T	Gln314His	[0.11199999999999999, 0.0484]

S	943A>T	Thr315Ser	[0.11199999999999999, 0.0484]
S	944C>T	Thr315Ile	[0.1232, 0.0376]
S	947C>G	Ser316Cys	[0.11199999999999999, 0.0376]
S	948T>G	Ser316Ser	[0.11199999999999999, 0.0484]
S	949A>G	Asn317Asp	[0.1148, 0.0376]
S	94T>A	Phe32Ile	[0.042]
S	950A>T	Asn317Ile	[0.11199999999999999, 0.0484]
S	951C>G	Asn317Lys	[0.11199999999999999, 0.0376]
S	95T>A	Phe32Tyr	[0.042]
S	96C>G	Phe32Leu	[0.042]
orf1ab	10006G>A	Val3336Ile	[0.04225]
orf1ab	10014C>T	Leu3338Leu	[1.0, 1.0, 1.0]
orf1ab	10052A>T	Lys3351Met	[0.0961]
orf1ab	10054C>T	Leu3352Phe	[0.0414]
orf1ab	10205C>T	Ser3402Leu	[0.0491]
orf1ab	10212T>A	Leu3404Leu	[0.0312]
orf1ab	10232T>C	Val3411Ala	[0.0439]
orf1ab	10234G>A	Gly3412Ser	[0.043]
orf1ab	10236T>C	Gly3412Gly	[0.0448]
orf1ab	10242C>T	Asn3414Asn	[1.0, 0.0569]
orf1ab	10285G>A	Glu3429Lys	[0.0857, 0.064]
orf1ab	1029T>C	Phe343Phe	[0.0535]
orf1ab	10340C>T	Pro3447Leu	[0.0358]
orf1ab	10354C>A	Gln3452Lys	[0.0449]
orf1ab	10361C>T	Ala3454Val	[0.0651]
orf1ab	10447C>T	Leu3483Phe	[1.0]
orf1ab	10524C>T	As508Asp	[0.0582]
orf1ab	10536A>T	Ile3512Ile	[0.0476]
orf1ab	10537C>T	Leu3513Leu	[0.0775]
orf1ab	10538T>C	Leu3513Pro	[0.0343]
orf1ab	10540G>T	Gly3514*	[0.0317]

orf1ab	10550C>T	Ser3517Phe	[0.0324, 1.0, 0.9602]
orf1ab	10580T>C	Met3527Thr	[0.0476]
orf1ab	10609A>G	Asn3537Asp	[0.40715]
orf1ab	10651G>T	Glu3551*	[0.0621]
orf1ab	10664C>T	Thr3555Ile	[0.0621]
orf1ab	10666C>T	Pro3556Ser	[0.1164]
orf1ab	10702T>C	Phe3568Leu	[0.0493]
orf1ab	10732G>A	Gly3578Ser	[0.0355]
orf1ab	10810T>C	Phe3604Leu	[0.0417, 0.0367, 0.0368]
orf1ab	10811T>C	Phe3604Ser	[0.047]
orf1ab	10818G>T	Leu3606Phe	[0.0304, 0.2518, 0.5027]
orf1ab	10854T>C	Ile3618Ile	[0.0394]
orf1ab	10856T>C	Ile3619Thr	[0.0328]
orf1ab	10859C>T	Ala3620Val	[0.0876]
orf1ab	10868C>T	Ala3623Val	[0.0358]
orf1ab	10873G>T	Ala3625Ser	[0.9496]
orf1ab	10887C>A	Val3629Val	[0.0779]
orf1ab	10896G>A	Lys3632Lys	[0.6767]
orf1ab	10902A>T	Ala3634Ala	[0.0428]
orf1ab	10908C>T	Leu3636Leu	[0.0323]
orf1ab	10977G>A	Tr659*	[0.040999999999999995]
orf1ab	10977G>T	Tr659Cys	[0.0348]
orf1ab	10978G>A	Val3660Met	[0.040999999999999995]
orf1ab	10984C>T	Arg3662Cys	[0.37695]
orf1ab	1108T>C	Cys370Arg	[0.0838]
orf1ab	1109G>T	Cys370Phe	[0.0632]
orf1ab	11140G>T	Val3714Phe	[0.0579]
orf1ab	11152G>T	Val3718Phe	[1.0, 1.0, 1.0, 1.0, 0.9551, 1.0, 1.0, 1.0, 1.0, 0.0304]
orf1ab	11159A>T	Lys3720Ile	[0.0316]
orf1ab	1115C>T	Ala372Val	[1.0, 0.9679, 0.8876, 0.9481]
orf1ab	11172T>A	Gly3724Gly	[0.0577]

orf1ab	11361C>T	Gly3787Gly	[0.0439]
orf1ab	11389T>C	Phe3797Leu	[0.0554]
orf1ab	11439C>T	Tyr3813Tyr	[0.1216]
orf1ab	11477A>G	Gln3826Arg	[0.0335]
orf1ab	1151T>C	Leu384Pro	[0.162]
orf1ab	1152T>C	Leu384Leu	[0.1632]
orf1ab	11539G>T	Val3847Phe	[0.0605]
orf1ab	11651C>T	Ser3884Leu	[1.0]
orf1ab	11653T>G	Ser3885Ala	[1.0]
orf1ab	11685C>T	His3895His	[0.0432]
orf1ab	11691C>T	As897Asp	[1.0, 1.0]
orf1ab	11701G>T	Ala3901Ser	[0.0979]
orf1ab	11718A>G	Glu3906Glu	[0.07175000000000001, 0.0321]
orf1ab	11722T>C	Phe3908Leu	[0.03]
orf1ab	11726A>C	Glu3909Ala	[0.03]
orf1ab	11727A>C	Glu3909Asp	[0.03]
orf1ab	11731A>T	Met3911Leu	[0.0306]
orf1ab	11732T>C	Met3911Thr	[0.03]
orf1ab	11734G>A	Val3912Ile	[0.0303]
orf1ab	11735T>C	Val3912Ala	[0.0303]
orf1ab	11738C>T	Ser3913Leu	[0.03]
orf1ab	11756T>C	Leu3919Pro	[0.0466]
orf1ab	11760C>T	Ser3920Ser	[1.0, 1.0]
orf1ab	11764C>T	Gln3922*	[0.0305]
orf1ab	11815G>A	Ala3939Thr	[0.0554]
orf1ab	11845A>G	Ser3949Gly	[0.5720000000000001]
orf1ab	11904T>C	Val3968Val	[0.0333]
orf1ab	11966C>T	Ser3989Phe	[0.0526]
orf1ab	1198G>A	Gly400Ser	[0.9352]
orf1ab	11995C>T	Arg3999Cys	[0.0373]
orf1ab	12031C>T	Gln4011*	[0.03]

orf1ab	12048T>C	Ala4016Ala	[0.0398]
orf1ab	12063G>T	Lys4021Asn	[0.0512]
orf1ab	12066G>A	Arg4022Arg	[0.036000000000000004]
orf1ab	1206C>T	Arg402Arg	[1.0]
orf1ab	12092C>A	Thr4031Lys	[0.0324]
orf1ab	12097C>T	Leu4033Phe	[0.0433]
orf1ab	12109C>T	Leu4037Phe	[0.031, 0.03]
orf1ab	1212T>C	Ile404Ile	[0.0602]
orf1ab	12138C>T	Asn4046Asn	[0.0326]
orf1ab	12194C>T	Thr4065Ile	[0.6927000000000001]
orf1ab	12260C>T	Thr4087Ile	[0.0544]
orf1ab	12292A>G	Ile4098Val	[0.0315]
orf1ab	12305T>C	Val4102Ala	[0.0907]
orf1ab	12335G>T	Ser4112Ile	[0.0329]
orf1ab	12336T>C	Ser4112Ser	[0.0333]
orf1ab	12404C>T	Ser4135Phe	[0.24855]
orf1ab	12418C>T	Gln4140*	[0.0377]
orf1ab	12426T>C	Asn4142Asn	[0.0396]
orf1ab	12436C>T	Pro4146Ser	[0.0329]
orf1ab	1245T>C	Gly415Gly	[0.0333]
orf1ab	1248C>T	Cys416Cys	[0.03]
orf1ab	1249C>T	His417Tyr	[0.05855]
orf1ab	1250A>G	His417Arg	[0.0325]
orf1ab	12554T>C	Leu4185Ser	[0.035]
orf1ab	12555A>G	Leu4185Leu	[1.0]
orf1ab	12590C>T	Pro4197Leu	[0.08495]
orf1ab	12615C>T	Ile4205Ile	[1.0, 1.0, 1.0]
orf1ab	12658C>T	Pro4220Ser	[0.06659999999999999]
orf1ab	12687C>T	Tyr4229Tyr	[0.0349]
orf1ab	12795T>G	Thr4265Thr	[0.0644]
orf1ab	12853G>T	Ala4285Ser	[0.0334]

orf1ab	12914G>T	Gly4305Val	[0.0318]
orf1ab	12918G>T	Gln4306His	[0.0305]
orf1ab	12932C>T	Thr4311Ile	[0.0331]
orf1ab	12955G>A	Glu4319Lys	[0.0332]
orf1ab	12960C>T	Ser4320Ser	[0.0437]
orf1ab	1297T>C	Cys433Arg	[0.9691, 0.9596]
orf1ab	1299T>C	Cys433Cys	[0.06355]
orf1ab	13138G>T	Gly4380Cys	[0.0331]
orf1ab	13158C>T	Leu4386Leu	[0.0338]
orf1ab	13165C>A	Pro4389Thr	[0.9663]
orf1ab	13184A>C	As395Ala	[0.0331]
orf1ab	13225G>C	Ala4409Pro	[0.0381]
orf1ab	13226C>T	Ala4409Val	[0.0381]
orf1ab	13227C>T	Ala4409Ala	[0.0383]
orf1ab	13228C>A	Arg4410Ser	[0.0381]
orf1ab	13229G>C	Arg4410Pro	[0.0381]
orf1ab	13239G>T	Pro4413Pro	[0.0637]
orf1ab	13277C>T	Ala4426Val	[0.0476]
orf1ab	13290C>T	Tyr4430Tyr	[0.0305]
orf1ab	13301T>C	Val4434Ala	[0.071]
orf1ab	13307G>T	Gly4436Val	[0.0542, 0.06225]
orf1ab	13312G>T	Ala4438Ser	[0.1096]
orf1ab	13313C>T	Ala4438Val	[0.1096]
orf1ab	13325A>T	Lys4442Ile	[0.0305]
orf1ab	1333G>C	Gly445Arg	[0.0326]
orf1ab	1334G>A	Gly445Asp	[0.033]
orf1ab	1336C>G	Leu446Val	[0.0334]
orf1ab	13491A>G	Arg4497Arg	[0.0637]
orf1ab	13535C>T	Thr4512Ile	[0.0301]
orf1ab	13548G>T	Met4516Ile	[0.0317]
orf1ab	13572G>A	Arg4524Arg	[0.0654]

orf1ab	13584A>T	Glu4528Asp	[0.0349]
orf1ab	13586G>T	Gly4529Val	[0.0328]
orf1ab	13637A>C	As546Ala	[0.0646]
orf1ab	13731T>C	Ala4577Ala	[0.0405]
orf1ab	13734G>T	Leu4578Phe	[0.0407]
orf1ab	13809T>C	As603Asp	[1.0, 1.0, 1.0, 1.0, 1.0, 1.0, 1.0, 1.0, 1.0]
orf1ab	13854G>A	Thr4618Thr	[0.1484]
orf1ab	13856C>T	Pro4619Leu	[0.1047]
orf1ab	13886A>C	Tyr4629Ser	[0.0402]
orf1ab	13927T>C	Leu4643Leu	[1.0, 1.0, 1.0]
orf1ab	13984T>C	Leu4662Leu	[0.0304]
orf1ab	1398C>T	As66Asp	[0.1793]
orf1ab	14078G>T	Cys4693Phe	[0.0477]
orf1ab	14132C>T	Thr4711Ile	[0.0352]
orf1ab	1413A>G	Glu471Glu	[0.04635]
orf1ab	14143C>T	Pro4715Ser	[1.0]
orf1ab	14144C>T	Pro4715Leu	[1.0, 1.0, 1.0, 1.0, 1.0, 1.0, 1.0, 1.0, 1.0, 1.0, 1.0, 1.0, 1.0, 1.0, 1.0, 1.0, 1.0, 1.0, 1.0, 1.0, 1.0, 1.0, 1.0, 1.0, 1.0, 1.0, 1.0, 1.0, 0.95775, 1.0, 1.0, 1.0, 1.0, 1.0, 1.0, 0.8492999999999999, 0.0552, 0.0572, 0.9194, 1.0, 1.0, 1.0, 1.0, 1.0, 1.0, 0.9627, 0.91355, 1.0, 1.0, 0.90985, 1.0, 1.0, 0.95815, 1.0, 1.0, 1.0, 1.0, 1.0, 1.0, 1.0, 1.0, 1.0, 1.0, 1.0, 1.0, 1.0, 1.0, 1.0, 1.0, 1.0, 1.0, 1.0, 1.0, 0.96675, 1.0, 1.0, 1.0, 1.0, 1.0, 1.0, 1.0, 1.0, 1.0]
orf1ab	14160A>G	Pro4720Pro	[0.10905]
orf1ab	14168G>A	Arg4723Lys	[0.0452]
orf1ab	14235T>C	Val4745Val	[0.0306]
orf1ab	14236G>T	Val4746Leu	[0.0398]
orf1ab	14256C>T	Asn4752Asn	[0.0453]
orf1ab	14276G>T	Ser4759Ile	[0.0479]
orf1ab	14424C>T	Asn4808Asn	[0.0331]
orf1ab	14463T>C	Phe4821Phe	[0.0644]
orf1ab	14467G>T	Glu4823*	[0.1227]
orf1ab	14488A>T	Lys4830*	[0.0465]
orf1ab	14522C>T	Ala4841Val	[0.0361]
orf1ab	1458T>C	Ala486Ala	[0.0787]
orf1ab	1465G>A	Glu489Lys	[0.0713, 0.0309]

orf1ab	14673C>T	As891Asp	[1.0, 1.0, 1.0, 0.9694, 0.922, 1.0, 1.0, 0.9667, 1.0, 0.96025]
orf1ab	14697T>A	Asn4899Lys	[0.0358]
orf1ab	14700A>T	Lys4900Asn	[0.0362]
orf1ab	14703G>T	Tr901Cys	[0.0367]
orf1ab	14704G>A	Gly4902Ser	[0.0362]
orf1ab	14713A>G	Arg4905Gly	[0.0337]
orf1ab	14746C>A	Gln4916Lys	[0.0437]
orf1ab	14747A>C	Gln4916Pro	[0.0426]
orf1ab	14756T>C	Leu4919Pro	[0.0336]
orf1ab	14773C>T	Arg4925Cys	[0.0498]
orf1ab	14775T>A	Arg4925Arg	[0.0443]
orf1ab	14802G>A	Met4934Ile	[0.0304]
orf1ab	14838T>C	Ala4946Ala	[0.0366]
orf1ab	1484A>G	As95Gly	[0.0302]
orf1ab	14875A>C	Thr4959Pro	[0.0325, 0.0336, 0.0334, 0.0319, 0.0312, 0.0305]
orf1ab	14877C>A	Thr4959Thr	[0.0341, 0.0352, 0.0309, 0.0315, 0.0319, 0.0335, 0.03375, 0.0309, 0.0312, 0.0314, 0.0323, 0.03465, 0.0321, 0.03, 0.0307, 0.0332, 0.0307, 0.0312, 0.0331, 0.0333, 0.0324, 0.0344, 0.0379, 0.038, 0.032]
orf1ab	14886G>T	Gln4962His	[0.0313, 0.0314, 0.0362, 0.0334, 0.0333, 0.0303, 0.031, 0.0331, 0.0509, 0.0315, 0.0364, 0.0308, 0.0583, 0.0367, 0.032, 0.0365, 0.0427, 0.03, 0.0391, 0.0438, 0.0331, 0.0467, 0.0449, 0.0329, 0.0405, 0.0411, 0.0432, 0.0427, 0.037000000000000005, 0.0517, 0.0302, 0.033, 0.04515, 0.0463, 0.0311, 0.0316, 0.046450000000000005, 0.0564, 0.0492, 0.0392, 0.0388, 0.03885, 0.0474, 0.0494, 0.0393, 0.054000000000000006, 0.0573, 0.0424, 0.0367, 0.0439, 0.0331, 0.0513, 0.0388, 0.0537, 0.0546, 0.0402, 0.0603, 0.0428, 0.0493, 0.0436, 0.0305, 0.0612, 0.0523, 0.043]
orf1ab	14893C>A	Gln4965Lys	[0.077, 0.05305, 0.0454, 0.0593, 0.0604, 0.0444, 0.05445, 0.05165, 0.0552, 0.060550000000000014, 0.0823, 0.1275, 0.0772, 0.1013, 0.1009, 0.0806, 0.0859, 0.0886, 0.0851, 0.0947, 0.0773, 0.0584, 0.0349]
orf1ab	14903T>C	Leu4968Ser	[0.0503]
orf1ab	14904G>A	Leu4968Leu	[0.0497, 0.0498, 0.0325, 0.0526, 0.0371, 0.0528, 0.0375, 0.0527, 0.06605, 0.0412, 0.0457, 0.0764, 0.07085, 0.0332, 0.0338, 0.0902, 0.0323, 0.0322, 0.03885, 0.0708, 0.0347, 0.0446, 0.0365, 0.0392, 0.0392, 0.08460000000000001, 0.0449, 0.0441, 0.06405, 0.0567, 0.0502, 0.062050000000000015, 0.06445, 0.0547, 0.06459999999999999, 0.0885, 0.0589, 0.0701, 0.0944, 0.0644, 0.06115, 0.06570000000000001, 0.0558, 0.0907, 0.0643, 0.0615, 0.0651, 0.0592, 0.066, 0.1001, 0.0925, 0.0378, 0.0651, 0.0453, 0.06205, 0.06265, 0.05995, 0.0386]
orf1ab	1496T>C	Phe499Ser	[0.0405]
orf1ab	15016C>T	Leu5006Phe	[0.0358]
orf1ab	15024T>C	Gly5008Gly	[0.1724]
orf1ab	15031T>C	Tyr5011His	[0.0303]
orf1ab	15063G>T	Met5021Ile	[0.0672]
orf1ab	15068G>A	Arg5023Lys	[0.0601]

orf1ab	15090T>C	Leu5030Leu	[1.0, 1.0, 0.8969499999999999, 0.93905, 1.0, 1.0, 1.0, 1.0, 1.0, 0.9511, 1.0, 1.0, 0.9653, 1.0, 1.0, 1.0, 1.0, 1.0]
orf1ab	15102T>C	His5034His	[1.0, 0.9426, 1.0, 1.0]
orf1ab	1542A>G	Gly514Gly	[0.0314]
orf1ab	15430C>G	His5144Asp	[0.0585]
orf1ab	15468G>T	Val5156Val	[1.0, 1.0]
orf1ab	15474C>T	Phe5158Phe	[1.0, 0.0821]
orf1ab	15496G>A	Gly5166Ser	[1.0, 1.0, 0.9613]
orf1ab	15541C>T	Gln5181*	[0.0542]
orf1ab	15596A>G	Lys5199Arg	[0.0624]
orf1ab	15678A>C	Pro5226Pro	[1.0, 0.41, 0.82545]
orf1ab	15688C>G	Leu5230Val	[0.0687]
orf1ab	15701G>T	Cys5234Phe	[0.055]
orf1ab	15719T>A	Val5240Glu	[0.0438]
orf1ab	15785C>T	Thr5262Ile	[0.0412]
orf1ab	15809C>T	Ala5270Val	[0.0383]
orf1ab	15842G>A	Arg5281Lys	[0.0441]
orf1ab	15988G>A	Val5330Ile	[0.0559]
orf1ab	15996C>T	Cys5332Cys	[1.0, 0.94535, 1.0, 1.0, 0.9677, 1.0, 0.95715]
orf1ab	16033C>A	Arg5345Ser	[0.0806]
orf1ab	16065C>T	Tyr5355Tyr	[0.13915]
orf1ab	16095A>G	Leu5365Leu	[0.0405]
orf1ab	16096G>T	Val5366Phe	[0.0401]
orf1ab	16111C>T	Pro5371Ser	[0.35200000000000004]
orf1ab	16117G>A	Val5373Ile	[0.9679, 0.915, 0.9119, 1.0, 1.0, 1.0, 1.0, 0.92565, 1.0, 1.0, 0.94865, 0.93945, 0.96715, 0.93005, 1.0, 1.0, 0.0308, 1.0, 0.8986000000000001, 0.91]
orf1ab	16201C>T	Pro5401Ser	[0.0472]
orf1ab	16285G>A	As429Asn	[0.4242]
orf1ab	16287C>T	As429Asp	[0.4242]
orf1ab	16292A>T	Asn5431Ile	[0.4242]
orf1ab	16296A>T	Ala5432Ala	[0.4242]
orf1ab	16303A>C	Thr5435Pro	[0.4242]
orf1ab	16304C>T	Thr5435Ile	[0.4242]

orf1ab	16322C>T	Ala5441Val	[0.0404]
orf1ab	16329T>A	As443Glu	[0.4242]
orf1ab	16330T>G	Tyr5444Asp	[0.4242]
orf1ab	16331A>G	Tyr5444Cys	[0.4242]
orf1ab	16401G>T	Glu5467Asp	[0.0329]
orf1ab	16402A>C	Thr5468Pro	[0.0446]
orf1ab	16403C>G	Thr5468Arg	[0.0446]
orf1ab	16404A>T	Thr5468Thr	[0.0384]
orf1ab	16407T>A	Phe5469Leu	[0.0442]
orf1ab	16408A>T	Lys5470*	[0.0446]
orf1ab	16409A>G	Lys5470Arg	[0.0442]
orf1ab	16415C>G	Ser5472Cys	[0.0442]
orf1ab	16416T>A	Ser5472Ser	[0.0446]
orf1ab	16417T>C	Tyr5473His	[0.0341]
orf1ab	16420G>C	Gly5474Arg	[0.0442]
orf1ab	16421G>A	Gly5474Asp	[0.0446]
orf1ab	16422T>G	Gly5474Gly	[0.0442]
orf1ab	16424T>C	Ile5475Thr	[0.0446]
orf1ab	16425T>A	Ile5475Ile	[0.0442]
orf1ab	16426G>A	Ala5476Thr	[0.0447]
orf1ab	16427C>T	Ala5476Val	[0.0325]
orf1ab	16435C>G	Arg5479Gly	[0.0442]
orf1ab	16436G>T	Arg5479Leu	[0.0466]
orf1ab	1644C>T	Phe548Phe	[0.0538]
orf1ab	16465C>T	Leu5489Phe	[0.0404]
orf1ab	1647C>T	Ser549Ser	[0.04]
orf1ab	16582G>T	As528Tyr	[0.4901]
orf1ab	16592A>G	As531Gly	[0.03305]
orf1ab	16602T>C	Val5534Val	[0.0416]
orf1ab	16613C>T	Thr5538Ile	[0.0465]
orf1ab	16633G>T	Val5545Phe	[0.0517]

orf1ab	1664C>T	Ala555Val	[0.9582]
orf1ab	16720A>G	Thr5574Ala	[0.91705]
orf1ab	16785G>A	Lys5595Lys	[0.0345]
orf1ab	16858G>T	Ala5620Ser	[1.0, 0.0708]
orf1ab	16903C>T	His5635Tyr	[0.0338]
orf1ab	16914T>G	Val5638Val	[0.0387]
orf1ab	17035T>C	Tyr5679His	[0.0874]
orf1ab	17077G>A	As693Asn	[0.0408]
orf1ab	17109C>T	Ala5703Ala	[0.0318]
orf1ab	17111C>T	Thr5704Ile	[0.0806]
orf1ab	17197C>A	Arg5733Ser	[0.0426]
orf1ab	17229	17230isCGTAGGAATGTGGCAACTTTACAAGCTGAAAATGTAACAGGACTCTTTAAA	[0.0486]
orf1ab	17232A>T	Glu5744Asp	[0.051]
orf1ab	17238C>T	Phe5746Phe	[0.0307]
orf1ab	17247G>T	Val5749Val	[0.0394]
orf1ab	17259G>A	Met5753Ile	[0.0591]
orf1ab	17259G>T	Met5753Ile	[0.03325]
orf1ab	17260A>G	Lys5754Glu	[0.0301]
orf1ab	17286C>T	Leu5762Leu	[0.0601]
orf1ab	17296C>T	Arg5766Trp	[0.0389]
orf1ab	17324C>T	Thr5775Ile	[0.0405]
orf1ab	17375C>T	Ser5792Leu	[0.0414]
orf1ab	17513C>T	Pro5838Leu	[0.040999999999999995]
orf1ab	17594A>G	Tyr5865Cys	[0.90665, 1.0]
orf1ab	175G>T	Gly59Cys	[0.0348]
orf1ab	17602G>C	Val5868Leu	[0.0338]
orf1ab	17613T>C	Thr5871Thr	[0.94075, 0.87055, 0.8836999999999999, 1.0, 1.0, 0.9191, 1.0, 0.9465, 1.0, 1.0, 0.9572, 0.9313, 0.96445, 0.94045, 1.0, 1.0, 1.0, 0.8799, 0.92005]
orf1ab	17622T>C	Thr5874Thr	[0.0434]
orf1ab	17638T>C	Cys5880Arg	[0.0409]
orf1ab	17646A>T	Val5882Val	[0.0695]

orf1ab	17648A>C	Asn5883Thr	[0.0695]
orf1ab	17649C>T	Asn5883Asn	[0.0695]
orf1ab	17650A>G	Arg5884Gly	[0.0695]
orf1ab	17651G>A	Arg5884Lys	[0.0695]
orf1ab	17652A>C	Arg5884Ser	[0.0695]
orf1ab	1770G>T	Leu590Phe	[1.0, 1.0]
orf1ab	17743G>A	Glu5915Lys	[0.0357]
orf1ab	17803G>T	As935Tyr	[0.0418]
orf1ab	17824G>A	Gly5942Arg	[0.0304]
orf1ab	17868T>A	Thr5956Thr	[0.0323]
orf1ab	17965T>C	Tyr5989His	[0.0873]
orf1ab	17967T>C	Tyr5989Tyr	[0.0579]
orf1ab	17978G>T	Gly5993Val	[0.1305]
orf1ab	18050A>T	Glu6017Val	[0.0511]
orf1ab	18113C>T	Thr6038Ile	[1.0]
orf1ab	1811G>T	Gly604Val	[0.0338]
orf1ab	18148G>T	Val6050Phe	[0.0335]
orf1ab	18160A>G	Asn6054Asp	[0.037000000000000005]
orf1ab	18180A>T	Arg6060Ser	[0.0308]
orf1ab	18183	18184isGTCAATGCCAGATTA	[0.03]
orf1ab	18193C>G	Pro6065Ala	[0.0308]
orf1ab	18207T>C	As069Asp	[0.1291]
orf1ab	18217C>T	His6073Tyr	[1.0]
orf1ab	18291C>T	As097Asp	[0.0389, 0.0319, 0.0649]
orf1ab	18293C>T	Thr6098Ile	[0.033600000000000005]
orf1ab	18309T>C	Ser6103Ser	[0.051]
orf1ab	18321A>G	Val6107Val	[0.04315]
orf1ab	18423C>T	Cys6141Cys	[0.0465]
orf1ab	18439G>T	As147Tyr	[0.032]
orf1ab	18459T>A	His6153Gln	[0.0551]
orf1ab	1848C>T	Ile616Ile	[0.57955]

orf1ab	18559C>T	Gln6187*	[0.0336]
orf1ab	18562G>T	Val6188Phe	[0.07]
orf1ab	18577C>T	His6193Tyr	[0.0385]
orf1ab	18600C>T	Ile6200Ile	[0.0401]
orf1ab	18613C>T	Leu6205Leu	[1.0, 1.0, 1.0, 1.0, 1.0, 1.0, 1.0, 1.0, 1.0, 0.8632500000000001, 0.9203, 1.0, 1.0, 1.0, 1.0, 1.0, 0.9346, 1.0, 1.0, 0.9552, 0.96635, 1.0, 1.0, 0.9579, 1.0]
orf1ab	18616G>T	Ala6206Ser	[0.0444]
orf1ab	18626A>G	Glu6209Gly	[0.0458]
orf1ab	18631T>C	Phe6211Leu	[0.0306]
orf1ab	1866A>G	Glu622Glu	[0.0393]
orf1ab	18750A>T	Lys6250Asn	[0.0557]
orf1ab	1879G>C	Val627Leu	[0.0629]
orf1ab	18805G>T	Ala6269Ser	[0.0462]
orf1ab	1881C>T	Val627Val	[0.0629]
orf1ab	1882C>T	Leu628Phe	[0.0303]
orf1ab	18879T>C	Tyr6293Tyr	[1.0, 1.0]
orf1ab	18893A>T	His6298Leu	[0.0534]
orf1ab	18907A>G	Thr6303Ala	[0.0506]
orf1ab	18975A>T	Arg6325Ser	[0.0482]
orf1ab	18983C>T	Thr6328Ile	[0.0413]
orf1ab	19080T>C	Ala6360Ala	[0.0384]
orf1ab	19111T>C	Tyr6371His	[0.0411]
orf1ab	19131T>C	Cys6377Cys	[0.2123]
orf1ab	19136C>T	Ser6379Phe	[0.0479]
orf1ab	19161T>C	As387Asp	[0.0328]
orf1ab	19184C>A	Ser6395Tyr	[0.0453]
orf1ab	19192T>C	Cys6398Arg	[0.0447]
orf1ab	19223T>C	Val6408Ala	[0.0564]
orf1ab	19254G>T	Leu6418Phe	[0.0346]
orf1ab	19413G>T	Gln6471His	[1.0, 1.0, 1.0, 1.0, 1.0, 1.0, 1.0, 1.0, 1.0, 1.0]
orf1ab	1945G>T	Val649Phe	[0.0307]
orf1ab	19483T>G	Phe6495Val	[0.0307]

orf1ab	19542C>T	Asn6514Asn	[1.0, 0.96975]
orf1ab	19560G>A	Glu6520Glu	[0.0304]
orf1ab	19575T>C	Asn6525Asn	[1.0, 1.0]
orf1ab	19604C>T	Thr6535Ile	[0.0955]
orf1ab	19687C>A	Pro6563Thr	[0.03495]
orf1ab	19694A>G	Glu6565Gly	[0.0394]
orf1ab	19709C>T	Pro6570Leu	[0.0571]
orf1ab	19719C>T	Val6573Val	[0.0356]
orf1ab	19733G>T	Arg6578Ile	[0.0772]
orf1ab	19774G>A	Gly6592Ser	[0.1228]
orf1ab	19792G>A	Gly6598Ser	[1.0, 0.95975, 1.0]
orf1ab	19820T>C	Val6607Ala	[0.0512]
orf1ab	19840C>T	Leu6614Phe	[0.03]
orf1ab	19846G>T	Gly6616*	[0.0772]
orf1ab	19858A>T	Ile6620Phe	[0.031]
orf1ab	19879C>T	Gln6627*	[0.0442, 0.0328]
orf1ab	19881G>A	Gln6627Gln	[0.0383]
orf1ab	19884C>T	Phe6628Phe	[0.9577, 1.0, 0.9648, 0.9444, 0.88185, 0.9512, 0.9584, 0.9638, 0.96295, 0.9486, 0.0532]
orf1ab	19885A>G	Asn6629Asp	[0.0383]
orf1ab	198G>C	Gln66His	[0.2283]
orf1ab	20004A>G	Leu6668Leu	[0.9673, 0.9338, 0.94435, 0.96405]
orf1ab	20052C>T	Phe6684Phe	[0.94375, 0.8966, 0.871, 0.9561, 1.0, 0.9245, 1.0, 0.90435, 0.96965, 0.96235, 0.89645, 0.9244, 0.9226, 0.94245, 0.96165, 1.0, 1.0, 0.85555, 0.8989]
orf1ab	20062G>A	Val6688Ile	[0.49405, 0.14265]
orf1ab	20076T>A	Phe6692Leu	[0.032]
orf1ab	20077A>C	Ser6693Arg	[0.032]
orf1ab	20078G>A	Ser6693Asn	[0.032]
orf1ab	20080C>T	His6694Tyr	[0.0629]
orf1ab	20081A>T	His6694Leu	[0.032]
orf1ab	20083A>T	Ser6695Cys	[0.0329]
orf1ab	20084G>C	Ser6695Thr	[0.032]
orf1ab	20085T>A	Ser6695Arg	[0.032]

orf1ab	20086C>T	Gln6696*	[0.032]
orf1ab	20087A>G	Gln6696Arg	[0.032]
orf1ab	20089T>G	Leu6697Val	[0.032]
orf1ab	2008G>A	Glu670Lys	[0.0306]
orf1ab	20090T>A	Leu6697*	[0.032]
orf1ab	20093G>T	Gly6698Val	[0.032]
orf1ab	20101C>T	His6701Tyr	[0.0312]
orf1ab	20102A>G	His6701Arg	[0.0312]
orf1ab	20110A>G	Ile6704Val	[0.0312]
orf1ab	20115A>T	Gly6705Gly	[0.032]
orf1ab	20134G>A	Glu6712Lys	[0.0493]
orf1ab	20161A>G	Ile6721Val	[0.1544]
orf1ab	20177C>T	Thr6726Ile	[0.033]
orf1ab	2026C>T	Gln676*	[0.0338]
orf1ab	20273T>C	Ile6758Thr	[0.0433]
orf1ab	20398A>G	Ser6800Gly	[0.0512]
orf1ab	2040G>T	Lys680Asn	[0.94765]
orf1ab	2041C>T	Leu681Phe	[0.043]
orf1ab	20422G>A	Ala6808Thr	[0.0591]
orf1ab	20451A>G	Arg6817Arg	[0.0452]
orf1ab	20455C>T	Leu6819Leu	[0.03]
orf1ab	20488G>T	As830Tyr	[1.0]
orf1ab	20491A>C	Ser6831Arg	[0.9686]
orf1ab	20495C>T	Ala6832Val	[0.0534]
orf1ab	20509G>T	Gly6837Cys	[0.036000000000000004]
orf1ab	205G>A	Val69Met	[0.0327]
orf1ab	20653C>T	Gln6885*	[0.0636]
orf1ab	20655G>T	Gln6885His	[0.0636]
orf1ab	20873A>G	Lys6958Arg	[1.0]
orf1ab	20907A>T	Ile6969Ile	[0.0668]
orf1ab	20957C>T	Ala6986Val	[0.0511]

orf1ab	20968G>T	Ala6990Ser	[0.0447]
orf1ab	20978C>T	Thr6993Ile	[0.1103]
orf1ab	209T>C	Phe70Ser	[0.2283]
orf1ab	21040C>A	Arg7014Ser	[0.05525]
orf1ab	21041G>A	Arg7014His	[0.05525]
orf1ab	21244G>A	Glu7082Lys	[0.0337]
orf1ab	21254G>C	Arg7085Thr	[0.0395]
orf1ab	21259G>A	Val7087Ile	[0.1056]
orf1ab	2138C>A	Ser713*	[0.0597]
orf1ab	2149T>C	Tyr717His	[0.04]
orf1ab	2151C>T	Tyr717Tyr	[1.0, 1.0, 1.0, 1.0, 1.0]
orf1ab	2205C>T	Ala735Ala	[0.62595]
orf1ab	2212G>A	Glu738Lys	[0.0308]
orf1ab	2221T>G	Phe741Val	[0.0512]
orf1ab	2284G>A	As62Asn	[1.0, 1.0]
orf1ab	2347G>C	Val783Leu	[0.1002]
orf1ab	2357A>G	Asn786Ser	[0.0826]
orf1ab	2367G>T	Met789Ile	[0.0472]
orf1ab	2374G>A	Glu792Lys	[0.0352]
orf1ab	2404C>T	Leu802Phe	[0.0306, 0.0468]
orf1ab	2433T>C	Asn811Asn	[0.0465]
orf1ab	255G>A	Met85Ile	[0.0396]
orf1ab	256G>T	Val86Phe	[0.0348]
orf1ab	2614G>T	Ala872Ser	[0.0838]
orf1ab	261G>T	Glu87Asp	[0.0304]
orf1ab	2674C>T	Pro892Ser	[1.0, 1.0]
orf1ab	2708C>T	Ala903Val	[0.0374]
orf1ab	2715C>T	Tyr905Tyr	[0.0378]
orf1ab	2772C>T	Phe924Phe	[0.96855, 1.0, 1.0, 0.96385, 1.0, 0.95695, 1.0, 1.0, 1.0, 1.0, 1.0, 1.0, 1.0, 1.0, 1.0, 1.0, 1.0, 1.0, 0.9641, 1.0, 0.91795, 1.0, 1.0, 1.0, 1.0, 1.0, 1.0, 1.0, 0.93995, 0.9696, 1.0, 1.0, 1.0, 1.0, 0.9267, 0.90875, 0.89975, 1.0, 1.0, 1.0, 1.0, 0.96895, 1.0, 1.0, 0.946, 1.0, 1.0, 0.9615, 0.9628, 0.95165, 1.0, 1.0, 1.0, 1.0, 1.0, 1.0, 1.0, 1.0, 1.0, 0.9652, 1.0, 1.0, 0.9512, 1.0, 1.0, 1.0, 0.96885, 1.0, 1.0, 1.0, 1.0, 0.96265, 1.0, 1.0, 1.0, 1.0, 1.0, 1.0, 1.0, 1.0, 0.96275]
orf1ab	2800G>T	Gly934Cys	[0.0328, 0.0561]

orf1ab	2964T>C	Val988Val	[0.0302]
orf1ab	2978G>A	Gly993Asp	[0.0324]
orf1ab	2994G>T	Gln998His	[0.0353]
orf1ab	3002C>T	Thr1001Ile	[0.0835]
orf1ab	3007C>T	Gln1003*	[0.039]
orf1ab	3021G>A	Glu1007Glu	[0.0395]
orf1ab	3077A>G	Asn1026Ser	[0.0324]
orf1ab	3102T>G	Leu1034Leu	[0.0611]
orf1ab	323T>C	Val108Ala	[0.0317]
orf1ab	3299G>T	Gly1100Val	[0.0311]
orf1ab	3317G>A	Ser1106Asn	[0.03955]
orf1ab	3373G>A	Gly1125Ser	[0.1018]
orf1ab	337G>A	Glu113Lys	[0.0307]
orf1ab	3399T>C	Ser1133Ser	[0.053]
orf1ab	3427G>T	Val1143Phe	[1.0, 1.0, 1.0, 1.0, 0.96215]
orf1ab	3433C>T	Leu1145Phe	[0.037000000000000005]
orf1ab	3440C>T	Pro1147Leu	[0.0496]
orf1ab	3448T>C	Ser1150Pro	[0.0731]
orf1ab	3451G>T	Ala1151Ser	[0.0746]
orf1ab	3452C>T	Ala1151Val	[0.1269]
orf1ab	3531C>T	Val1177Val	[0.0339]
orf1ab	354C>T	Tyr118Tyr	[1.0, 1.0, 0.96725, 1.0]
orf1ab	3601C>T	Gln1201*	[0.0771]
orf1ab	3606G>T	Lys1202Asn	[1.0, 1.0, 0.96725, 1.0, 1.0, 1.0, 0.96825, 1.0, 1.0, 1.0, 0.95965, 0.9627]
orf1ab	3609C>T	Ile1203Ile	[0.12055]
orf1ab	3650A>C	Glu1217Ala	[0.0478]
orf1ab	3659C>T	Pro1220Leu	[0.0307]
orf1ab	3666T>C	Val1222Val	[1.0, 1.0, 0.9679, 1.0, 1.0, 1.0, 0.96805, 1.0, 1.0, 1.0, 0.9590000000000001, 0.9628]
orf1ab	3674G>A	Arg1225Lys	[0.0667]
orf1ab	370C>T	Arg124Cys	[0.0304]
orf1ab	3751G>A	Glu1251Lys	[0.0469]

orf1ab	3763C>T	Leu1255Phe	[0.0346]
orf1ab	3830C>T	Thr1277Ile	[0.0333]
orf1ab	3832T>C	Phe1278Leu	[0.0305]
orf1ab	3953G>C	Arg1318Thr	[0.0388]
orf1ab	3961C>T	Pro1321Ser	[1.0, 1.0, 1.0, 1.0, 1.0, 1.0, 1.0, 1.0, 1.0, 1.0, 1.0]
orf1ab	3982A>C	Thr1328Pro	[0.0309]
orf1ab	3983C>T	Thr1328Ile	[0.0311]
orf1ab	3989C>A	Pro1330Gln	[0.0325]
orf1ab	4000T>C	Leu1334Leu	[0.0357]
orf1ab	4017A>T	Val1339Val	[0.0756]
orf1ab	4070C>T	Pro1357Leu	[0.0617]
orf1ab	4121A>C	Asn1374Thr	[0.0325]
orf1ab	4136T>C	Leu1379Pro	[0.0302]
orf1ab	413C>T	Ala138Val	[0.0321]
orf1ab	4163T>G	Leu1388*	[0.0705]
orf1ab	4188A>G	Lys1396Lys	[0.0709]
orf1ab	4189G>T	Ala1397Ser	[0.0493]
orf1ab	4191C>A	Ala1397Ala	[0.0709]
orf1ab	4258G>A	Ala1420Thr	[0.95695]
orf1ab	4277C>T	Thr1426Ile	[0.0334]
orf1ab	4280G>T	Ser1427Ile	[0.0828]
orf1ab	4286C>T	Thr1429Ile	[0.0356]
orf1ab	4299A>G	Ser1433Ser	[0.0587]
orf1ab	431A>C	As44Ala	[1.0, 1.0, 1.0, 1.0, 1.0, 1.0, 1.0, 1.0, 1.0, 1.0, 1.0, 1.0]
orf1ab	4327G>A	Glu1443Lys	[0.1441]
orf1ab	4447G>C	Val1483Leu	[0.0323]
orf1ab	4489C>T	Pro1497Ser	[0.0324]
orf1ab	4507G>A	Glu1503Lys	[0.0448]
orf1ab	4521T>C	Leu1507Leu	[0.047]
orf1ab	4568T>C	Leu1523Pro	[0.0325]
orf1ab	4570G>T	Gly1524Cys	[0.135]

orf1ab	4573A>G	Ile1525Val	[1.0, 1.0, 1.0, 1.0, 0.94305, 1.0, 1.0, 1.0, 1.0, 1.0]
orf1ab	4579T>A	Phe1527Ile	[0.1048]
orf1ab	4620T>G	Asn1540Lys	[0.0383]
orf1ab	4636C>T	Leu1546Leu	[0.07569999999999999]
orf1ab	4649T>C	Val1550Ala	[0.0301]
orf1ab	4652T>C	Ile1551Thr	[1.0]
orf1ab	468T>A	As56Glu	[0.0657]
orf1ab	4814C>T	Thr1605Ile	[0.0316]
orf1ab	4879C>T	Leu1627Leu	[0.32825]
orf1ab	48C>T	Leu16Leu	[0.965]
orf1ab	4913C>T	Thr1638Ile	[0.0444]
orf1ab	4915G>T	As639Tyr	[0.0444]
orf1ab	4938C>T	Tyr1646Tyr	[0.0324]
orf1ab	4943C>T	Ser1648Leu	[0.0375]
orf1ab	4959T>A	Thr1653Thr	[0.0503]
orf1ab	5071A>G	Asn1691Asp	[0.0392]
orf1ab	5074C>T	Pro1692Ser	[1.0, 1.0, 1.0, 0.96655]
orf1ab	5100C>G	Tyr1700*	[0.0401]
orf1ab	5134G>T	Ala1712Ser	[0.0484]
orf1ab	5176G>A	Gly1726Ser	[0.1138]
orf1ab	5188G>A	Glu1730Lys	[0.045]
orf1ab	5247C>T	Asn1749Asn	[0.03665]
orf1ab	5256T>C	Cys1752Cys	[0.1678]
orf1ab	5269C>T	Gln1757*	[0.0495]
orf1ab	5280A>G	Thr1760Thr	[0.0669]
orf1ab	5386T>A	Tyr1796Asn	[0.033]
orf1ab	5386T>C	Tyr1796His	[0.0466]
orf1ab	5389C>T	Leu1797Leu	[0.0428]
orf1ab	5407C>T	Pro1803Ser	[1.0, 1.0, 1.0, 1.0, 1.0, 1.0, 1.0, 1.0, 1.0, 1.0, 1.0]
orf1ab	5434G>T	Ala1812Ser	[0.0509]
orf1ab	5436T>C	Ala1812Ala	[0.0509]

orf1ab	5445A>C	Glu1815Asp	[0.0539]
orf1ab	5470G>T	Ala1824Ser	[0.0303]
orf1ab	5514T>C	His1838His	[0.0499]
orf1ab	5519C>T	Thr1840Ile	[0.0411]
orf1ab	5520T>C	Thr1840Thr	[0.08245]
orf1ab	5561C>T	Thr1854Ile	[0.92305]
orf1ab	5569T>C	Ser1857Pro	[0.0463]
orf1ab	564C>T	Asn188Asn	[0.8724000000000001, 0.04005]
orf1ab	5665T>C	Cys1889Arg	[0.0431]
orf1ab	5666G>A	Cys1889Tyr	[0.0436]
orf1ab	5667T>C	Cys1889Cys	[0.0442, 0.0436]
orf1ab	5713T>G	Ser1905Ala	[0.0387]
orf1ab	5719T>A	Phe1907Ile	[0.0391]
orf1ab	5719T>G	Phe1907Val	[0.1507]
orf1ab	5720T>A	Phe1907Tyr	[0.1507]
orf1ab	5723C>G	Thr1908Arg	[0.1507]
orf1ab	5724A>C	Thr1908Thr	[0.1507]
orf1ab	5725G>A	Glu1909Lys	[0.1507]
orf1ab	5727G>A	Glu1909Glu	[0.0337]
orf1ab	5750A>G	Asn1917Ser	[0.15605000000000002]
orf1ab	5762C>A	Pro1921Gln	[0.0489]
orf1ab	5774T>A	Phe1925Tyr	[0.0816]
orf1ab	5775C>T	Phe1925Phe	[0.08199999999999999, 0.1544, 0.05925]
orf1ab	5776G>A	As926Asn	[0.0816]
orf1ab	5777A>G	As926Gly	[0.08199999999999999]
orf1ab	5809T>C	Phe1937Leu	[0.1345]
orf1ab	5819A>T	As940Val	[0.0427]
orf1ab	5865T>A	Leu1955Leu	[0.0491]
orf1ab	5872A>G	Thr1958Ala	[0.0512]
orf1ab	6047C>T	Thr2016Ile	[0.0336]
orf1ab	6052C>T	Pro2018Ser	[0.0908]

orf1ab	6117T>C	Leu2039Leu	[0.0547]
orf1ab	6137C>A	Pro2046Gln	[0.3248, 0.71935]
orf1ab	6155T>G	Val2052Gly	[0.1131]
orf1ab	6180C>T	As060Asp	[1.0, 1.0]
orf1ab	6184C>T	Leu2062Phe	[0.0472]
orf1ab	6189G>T	Glu2063Asp	[1.0]
orf1ab	6235C>T	Pro2079Ser	[1.0, 1.0]
orf1ab	6319A>G	Ile2107Val	[0.72085]
orf1ab	6330T>C	Pro2110Pro	[0.0708]
orf1ab	6331A>T	Asn2111Tyr	[0.0715]
orf1ab	6340T>C	Ser2114Pro	[0.1094]
orf1ab	6346G>T	Val2116Leu	[0.0735]
orf1ab	6348A>T	Val2116Val	[0.1099]
orf1ab	6351A>T	Leu2117Phe	[0.1087]
orf1ab	6352G>A	Gly2118Ser	[0.1131]
orf1ab	6357G>A	Leu2119Leu	[0.03065, 0.0428]
orf1ab	6358A>G	Lys2120Glu	[0.1087]
orf1ab	6359A>T	Lys2120Ile	[0.1087]
