# Supplementary material for: Acute SARS-CoV-2 infections harbor limited within-host diversity and transmit via tight transmission bottlenecks
Source: PLoS Pathog. 2021 Aug 23;17(8):e1009849. doi: 10.1371/journal.ppat.1009849 (PMC8412271; doi:10.1371/journal.ppat.1009849)
Supplement: Attachment — Submitted filename: response-to-reviewer-2021-07-24.docx [file ppat.1009849.s018.docx]

We thank the reviewers for their careful reading of the manuscript and helpful comments. We found these comments quite helpful and feel that they have improved the manuscript. Reviewer comments are shown in italics and responses are shown in plain text. For large sections of the manuscript that have been revised/added, we have included those sections in indented text. Please note that line numbers included here correspond to the revised manuscript that has been uploaded with the figures still embedded. We chose to do this for ease of reviewer comparison. We have also included a version of the revised manuscript with all revisions highlighted in tracked changes (with embedded figures) to highlight changes.

### Reviewer #1

1. *Reviewer #1: This study looks to understand the intra-host diversity (iSNVs) and transmission bottlenecks of SARS-CoV-2 during acute infection. The authors sequence SARS-CoV-2 genomes present in nasal swabs from acutely infected individuals obtained March to July 2020. They apply an array of well thought out controls to analyze their data and conclude that there is little diversity within hosts and the iSNVs present do not contribute significantly to global consensus changes. Moreover, they analyze household transmission pairs and conclude that transmission bottlenecks are likely narrow. Together, the authors conclude that due to low intra-host diversity and narrow transmission bottlenecks, SARS-CoV-2 evolution or emergence is limited in acutely infected individuals.*

We thank the reviewer for this succinct summary of our study.

2. *This study is technically well done with extreme care in their data analysis and controls. I think this work is important and confirms previously published studies. However, I’m finding it hard to see the absolute novelty of the conclusions when comparing this study to several others the authors mention. In addition, there are several major points to be addressed for publication.*

*My major concern with this study are with the conclusions drawn from the data. First, the title of this manuscript implies mechanism. As written, it states that limited within-host diversity actually limits SARS-CoV-2 evolution. I don’t think the authors have shown that here and from the manuscript they think the limited diversity implies the limited evolution. The title should be changed to show what the study found. Maybe something like “Individuals acutely infected with SARS-CoV-2 have limited within-host diversity and tight transmission bottlenecks”.*

This point is well taken. We have edited the title to more accurately reflect the results of our study. The title is now “Acute SARS-CoV-2 infections harbor limited within-host diversity and transmit via tight transmission bottlenecks.”

3. *Second, the way the manuscript is written, the authors conclusions are not very defined. For example, they write “Variation is shared among some household samples, but is likely insufficient…” “transmission bottlenecks are likely narrow”. Writing it this way makes me wonder, is it or isn’t it? There are a few other vague statements throughout the manuscript “Figure 4: Household pairs share a modest degrees of within-host variation”. What does modest degree mean? The authors did a great job analyzing the data and it’s correct, the conclusions should be strong.*

We agree with the reviewer and have clarified and strengthened our conclusions throughout the manuscript. We have made a concerted effort to remove ambiguous language and replace it with quantitative measures when possible. We made these changes extensively throughout the manuscript, which can be most easily viewed in the highlighted/tracked changed text in revised copy of the manuscript file.

4. *Last, some conclusions don’t seem to match up and the authors don’t justify why they conclude these things. For example, in the section “Most within-host variation does not contribute to consensus diversity” the authors state that 42% of iSNVs they find are found globally, yet in the caption of Supplemental Figure 7 they state that 1/3rd of iSNVs were detected. Which is it? I suppose that because this number is less than 50% the conclusion is correct but to me 42% is a lot and suggests that these iSNVs are out there.*

We thank the reviewer for catching this typo. The correct percentage is 42%, which has been fixed in the Supplemental Figure 7 legend. Regarding the interpretation of this fraction, the reviewer brings up a very salient point, and it is one that we have given some thought. Given the difficulty of determining a null expectation for the fraction of within-host variants that should make it to consensus frequency and be sampled in the tree, we agree that interpreting the meaning of this percentage is tricky. We have therefore opted to remove some of the over-interpretation we had in this selection of the results, and instead focus on reporting the percentage along with the distribution of variants on internal/terminal nodes. **Lines 259-261** now read,

“This suggests that although putative functional mutations may arise within a host, we do not see evidence for most iSNVs as selectively beneficial, in agreement with patterns observed in other viral pathogens [[35]](https://paperpile.com/c/gUWjkV/ENsRT).”

We have also changed the title of this section to be “Within-host variants are found only once in phylogenetically linked infections,” which we feel may better represent the results presented.

5. *In addition, they conclude in the discussion “our results imply that the accumulation of multiple iSNVs is unlikely during typical, acute infection” Why do you conclude that? In figure 1 you show that many individuals have 2+ mutations. Is there a reason for this conclusion beyond the data? I could look at these data as even though most iSNVs are not found globally and most people have 1 iSNV, there are still many people with multiple iSNVs and lots of iSNVs found globally. I suggest editing the manuscript significantly to make sure all your conclusions are backed up by your strong data.*

We thank the reviewer for this comment, and have amended the manuscript, and extensively revised the discussion to clarify our conclusions and bring them in line with our results. We also recognize that we were not entirely clear in how we presented our interpretation and have modified it. Our interpretation is that our data, as well as findings from others, suggest that while multiple iSNVs may *arise* within an individual infection, that these mutations are usually low frequency and are rarely transmitted onward. Therefore, the likelihood that multiple de novo mutations will arise, be selected within-host to a high frequency, and be subsequently transmitted, is likely a rare event. Of course, even rare events can occur given sufficient chances, and our dataset represents only a small fraction of SARS-CoV-2 infections in Wisconsin. We have clarified the language in the introduction and results to clarify our interpretation, and added in these important caveats. We have also extensively revised the discussion to include a reframing of our results, add in caveats, and to respond to reviewer 2’s suggestions to shorten it. We feel that these changes have greatly improved the manuscript, and we hope you agree.

In the abstract, we have replaced the final sentence in **lines 32-33** to now read, “These findings suggest that most variation generated within-host is lost during transmission.”

In the introduction, we have amended **lines 110-120** to highlight that future studies will be necessary to confirm and extend our results and hypotheses. These lines now read:

“Our results imply that during typical, acute SARS-CoV-2 infections, the combination of limited intrahost genetic diversity and narrow transmission bottlenecks may slow the pace by which novel variants arise, are selected, and transmit onward. Finally, our findings are consistent with the hypothesis that novel variants are more likely to be selected to high frequencies during the course of prolonged infections, and that a minority of infections, either acute or prolonged, may play outsize roles in the emergence of novel variants. Followup studies that continue to dissect the degree with which individual infections, including both acute and chronic, contribute to global evolution will be necessary for confirming this hypothesis. Finally, future examination of how SARS-CoV-2 evolution proceeds in individuals with prior exposure via vaccination or infection will be necessary for extending these results as SARS-CoV-2 continues to circulate and evolve.”

In the discussion, we have revised the first paragraph, **lines 381-390**, to clarify our interpretation and add in caveats regarding limited sample size and the possibility or rare events given sufficient chances:

“The emergence of divergent SARS-CoV-2 lineages has called into question the role of within-host selection in propagating novel variants. Our results suggest that very limited variation is generated and transmitted during acute SARS-CoV-2 infection. Most infections in our dataset are characterized by fewer than 5 total intersection iSNVs, the majority of which are low-frequency. Less than half of iSNVs are not detected in global consensus genomes, and are rarely detected in downstream branches on the phylogenetic tree. We show that even among putative household transmission pairs, iSNVs are shared infrequently, and we estimate that a small number of viruses found infection after transmission. The combination of low within-host diversity, tight transmission bottlenecks, and infrequent propagation along transmission chains may slow the rate of novel variant emergence among acutely infected individuals.”

We have also cut some sections of the discussion and replaced them with 2 new paragraphs that highlight caveats to our study on **lines 436-464**:

“Our results lead to two main findings. The first is that in individual, acute infections, within-host diversity is limited. The second is that most iSNVs that are present within hosts are lost during transmission. Our results imply that the de novo generation, within-host selection, and subsequent transmission of mutations during the confines of an acute infection is rare. Of course, our dataset represents only a small fraction of the SARS-CoV-2 infections in Wisconsin, and even the combination of every existing within-host dataset represents only a marginal fraction of the SARS-CoV-2 transmission events that have occurred. Indeed, with 17 transmission pairs, our study is currently the second-largest to investigate SARS-CoV-2 evolution within and between hosts. Therefore, while our findings and those from others suggest that evolution is minimal during acute infection, even rare events can occur given sufficient opportunity. No individual study to date, including our own, is sufficiently powered to define how rare these events may be. High rates of SARS-CoV-2 transmission increase the likelihood of rare transmission events in which low-frequency variants are transmitted. Decreasing the number of infections globally through vaccination and non-pharmaceutical interventions are critical for reducing the opportunities for rare evolutionary events to occur. Ongoing assessment of SARS-CoV-2 evolution within and between individuals, especially in the face of growing immunity, will inform our understanding of how novel variants arise and sweep to fixation in the community and beyond.

Our findings are also consistent with, though do not prove, a regime in which most acute infections play a limited role in the generation and spread of new SARS-CoV-2 variants. Prolonged infections permit additional cycles of viral replication, lead to greater accumulation of intrahost mutations [[4–8]](https://paperpile.com/c/gUWjkV/fVYui+8vAnJ+iMYqA+kJfme+DDHD7), and allow more time for within-host selection. Even a modest increase in frequency enhances the likelihood of a beneficial variant being transmitted onward, and election during transmission may further propagate beneficial variants between hosts [[25]](https://paperpile.com/c/gUWjkV/QIxzs). It is therefore possible that the rare instances of prolonged infections play an outsize role in novel variant emergence. Future studies that characterize how variants arise, evolve, and transmit during persistent infections will be important for teasing apart the degree with which acute and prolonged infections contribute to global SARS-CoV-2 evolution.”

6. *On this note, try to distinguish your study from others done before. I found this hard to see reading through.*

The reviewer is correct in that many of the conclusions of our manuscript are supported by other published manuscripts. Unlike most other studies, we designed our study from the outset with the explicit focus on controlling for bioinformatic and laboratory error, and we took great care to ensure that our variant calls were reproducible. We feel that this is a major strength of the paper, and have highlighted this in the introduction on **lines 99-102**, which read: “Unlike other existing studies, we explicitly designed our investigation with controlling for bioinformatic and laboratory error in mind, sequencing every sample in technical replicate and validating our variant calls with an entirely separate bioinformatic pipeline,” and on **lines 398-402** in the discussion, “Our study is unique in that we designed our protocols with the specific intent of mitigating sources of bioinformatic and laboratory error. To this end, we have attempted to employ multiple, overlapping controls to mitigate errors that could arise from sample preparation, bioinformatic processing, and improper variant thresholds.”

7. *I found the way the iSNVs were presented a bit confusing. First, are there consensus changes in individuals? From the data, yes there are. The authors call them “Wuhan reversions” but they are actually consensus changes. These changes are very important but they are not talked about in Figure 1d. Along these same lines, in Figure 2, it would be very helpful to know the frequency of the variants in 2b. I think a Table showing all of the variants found, their frequencies, and their protein changes would be very helpful. It would also be helpful to have a genome schematic in the figure(s) to show where exactly on the genome each SNP is found.*

The reviewer is correct in that our “Wuhan reversions” are indeed consensus changes, however, we looked at the minor frequency of consensus change during this analysis. “For example, if a consensus change from C to T was found at 95%, we called the C at 5% an iSNV in our dataset.” has now been added on **lines 198-199s** to clarify.

We have added a new supplemental table and figure (**supplemental figure 12 and supplemental table 5**) depicting all SNVs, their genome location, and frequency in our dataset. These are referenced in **lines 203-205**: “**Supplemental table 5** lists all iSNV frequencies, genome locations, and protein changes (amino acid changes) detected in our dataset. A visualization of this information is shown in **supplemental figure 12.**”

8. *Finally, one thing that is important to address is how specific do you think these conclusions are to the samples you analyzed? You are analyzing swabs from March-July 2020, right at the beginning of the pandemic. If I remember, the D614G mutation is not in your reference genome and you don’t find it yet. So SARS-CoV-2 has not really started evolving. Do you think if you did the same analysis with samples from 2021 you’d get the same results? Maybe another study did this? I think this is important to address, as it is possible that an emerging variant in the SARS-CoV-2 nonstructural proteins could have changed the fidelity of SARS-CoV-2 to increase evolution. This is merely hand waving but I’m curious as to whether SARS-CoV-2 has increased its evolutionary capacity by adaptive mutations not in the Spike (which we tend to focus on). So maybe at the beginning of the pandemic your conclusions are correct yet, as the pandemic evolved, so did SARS-CoV-2 diversity/transmission bottlenecks. How your conclusions hold up over time would be important to discuss.*

The reviewer brings up an important point, and we agree that this is an important caveat that we did not address initially. We have added a new paragraph into the discussion to discuss this caveat and call attention to the need for future work to dissect how changes in the viral consensus may impact evolution and how evolution proceeds in response to population immunity. This new paragraph is on **lines 466-478**:

“An important limitation of our study is that all of our samples were collected between March and June of 2020. Our analysis therefore represents a snapshot in time prior to the emergence of variants of concern, and before a significant fraction of the population was immune. Dissecting how the fixation of variant of concern mutations and lineages may have impacted the evolutionary patterns of SARS-CoV-2 will be important future work. It is also important to note that patterns of within-host evolution may differ in individuals with vaccine or infection-induced immunity. Whether antigenic escape mutations arise and are selected within individuals with prior immune exposure remains an open question. Understanding the degree to which within-host evolution is shaped by vaccine and infection-induced immunity, both within-host and globally, will be critical for evaluating the pace of SARS-CoV-2 evolution in the future. Finally, while we did not have access to longitudinal samples in this study, characterization of viral populations over time in individual infections will provide superior resolution regarding the dynamicity of iSNVs and their frequencies throughout the course of infection.”

We have also acknowledge this caveat to the introduction on **lines 117-120**:

“Finally, future examination of how SARS-CoV-2 evolution proceeds in individuals with prior exposure via vaccination or infection will be necessary for extending these results as SARS-CoV-2 continues to circulate and evolve.”

### Reviewer #2

1. *This is an interesting study, which address an important question. In general, the study is technically sounded, but the authors should be more careful at the interpretations of the results and general implications. It would make a stronger report if the authors explicitly recognize the limitation of the study (i.e. number of patients, depth of the sequencing data, etc). See specific comments, but I also recommend to carefully revise the text to provide a more complete report of the caveat on the current study.*

We agree with this reviewer and have taken this comment to heart. We have added limitations and caveats into our paper in multiple sections, including several new paragraphs. In the discussion on **lines 436-453**, we highlight that our dataset is small, and that it is not powered to detect rare events. We also acknowledge that future work is necessary to refine rates of within-host evolution:

“Our results lead to two main findings. The first is that in individual, acute infections, within-host diversity is limited. The second is that most iSNVs that are present within hosts are lost during transmission. Our results imply that the de novo generation, within-host selection, and subsequent transmission of mutations during the confines of an acute infection is rare. Of course, our dataset represents only a small fraction of the SARS-CoV-2 infections in Wisconsin, and even the combination of every existing within-host dataset represents only a marginal fraction of the SARS-CoV-2 transmission events that have occurred. Indeed, with 17 transmission pairs, our study is currently the second-largest to investigate SARS-CoV-2 evolution within and between hosts. Therefore, while our findings and those from others suggest that evolution is minimal during acute infection, even rare events can occur given sufficient opportunity. No individual study to date, including our own, is sufficiently powered to define how rare these events may be. High rates of SARS-CoV-2 transmission increase the likelihood of rare transmission events in which low-frequency variants are transmitted. Decreasing the number of infections globally through vaccination and non-pharmaceutical interventions are critical for reducing the opportunities for rare evolutionary events to occur. Ongoing assessment of SARS-CoV-2 evolution within and between individuals, especially in the face of growing immunity, will inform our understanding of how novel variants arise and sweep to fixation in the community and beyond.”

In the discussion on **lines 466-478**, we added a paragraph to acknowledge that our dataset is limited in that it only includes samples from early in the pandemic, a caveat suggested by reviewer 1. We also acknowledge that we only have access to a single timepoint for each individual in our dataset:

“An important limitation of our study is that all of our samples were collected between March and June of 2020. Our analysis therefore represents a snapshot in time prior to the emergence of variants of concern, and before a significant fraction of the population was immune. Dissecting how the fixation of variant of concern mutations and lineages may have impacted the evolutionary patterns of SARS-CoV-2 will be important future work. It is also important to note that patterns of within-host evolution may differ in individuals with vaccine or infection-induced immunity. Whether antigenic escape mutations arise and are selected within individuals with prior immune exposure remains an open question. Understanding the degree to which within-host evolution is shaped by vaccine and infection-induced immunity, both within-host and globally, will be critical for evaluating the pace of SARS-CoV-2 evolution in the future. Finally, while we did not have access to longitudinal samples in this study, characterization of viral populations over time in individual infections will provide superior resolution regarding the dynamicity of iSNVs and their frequencies throughout the course of infection.”

In the discussion on **lines 427-434**, where placing our bottleneck estimates in context with findings from others, we have added in an explicit acknowledgement that our analysis of household pairs is a small dataset, and that it lacks epidemiologic data about alternative transmission routes beyond household membership:

“While current evidence is converging to support a narrow transmission bottleneck for SARS-CoV-2, similar to influenza virus [[18,43,44]](https://paperpile.com/c/gUWjkV/kHneS+g5OYy+s7BSz), more data is needed. Our analysis relied on anecdotal evidence of transmission, without epidemiological data to investigate alternative infection histories beyond household membership. It is therefore plausible that some of our transmission pairs were infected independently or from a common source. Future studies examining a larger number of transmission events will be necessary to further refine bottleneck estimates, and to determine whether factors like route of transmission [[45]](https://paperpile.com/c/gUWjkV/yMzX3) impact transmission bottleneck size.”

2. *The authors should remove the phrase “enhanced transmissibility” because this has not been thoroughly demonstrated for any of the variants. The main observations about variants of concern are their ability to replace some of the older variants in the population and/or evade to some degree neutralization by circulating antibodies. The 2 references (2 and 3) in the paper actually say the same thing.*

We agree with the reviewer and have removed this statement and implication throughout the manuscript. We have instead opted to write that these lineages have replaced other circulating variants, as suggested by the reviewer.

3. *Could the authors describe the dataset from households? In particular, how many samples/household? Were there more than one sample/individual? At what day post symptom onset? Also, in order to understand the rate of variant emergence during acute infection, the authors should detail when samples were collected after symptom onset.*

We have clarified the metadata around the dataset generated from the households on **lines 265-270** and have also explicitly called out that additional information about these households can be found in **supplemental table 4**. These lines now read: “We analyzed 44 samples collected from 19 households from which multiple individuals were infected with SARS-CoV-2 (more information on household structure, accession numbers, and days post symptom onset can be found in **supplemental table 4**). Only a single timepoint from each individual, with collection times relative to their symptomatic date ranging from asymptomatic or never symptomatic to 15 days after symptom onset, were collected and analyzed for this study.”

4. *What is the unit of bottleneck size?*

We have clarified this in the results on **lines 330-332,** “In this method, the unit of the transmission bottleneck is the number of viruses that found infection in a recipient host following transmission.” We have also added this into the Methods on **lines 655-656**: “The unit of the transmission bottleneck, as described by Sobel-Leonard and Koelle is the number of viruses that found infection in a recipient host following transmission.”

5. *Overall, the analysis done in this study is well controlled but one main question regarding the sample size of the study remains: Is the dataset big enough to rule out intrahost diversity’s role in variant emergence? A few chronic infections might play a major role in variant emergence but considering the large numbers of acute infections in the world population, even rare events in acute infection might play a significant role in it too.*

The reviewer brings up an excellent point and they are absolutely correct. In the discussion, we have reorganized several paragraphs and re-written them to explicitly lay out these possibilities. **Lines 436-464** now read:

“Our results lead to two main findings. The first is that in individual, acute infections, within-host diversity is limited. The second is that most iSNVs that are present within hosts are lost during transmission. Our results imply that the de novo generation, within-host selection, and subsequent transmission of mutations during the confines of an acute infection is rare. Of course, our dataset represents only a small fraction of the SARS-CoV-2 infections in Wisconsin, and even the combination of every existing within-host dataset represents only a marginal fraction of the SARS-CoV-2 transmission events that have occurred. Indeed, with 17 transmission pairs, our study is currently the second-largest to investigate SARS-CoV-2 evolution within and between hosts. Therefore, while our findings and those from others suggest that evolution is minimal during acute infection, even rare events can occur given sufficient opportunity. No individual study to date, including our own, is sufficiently powered to define how rare these events may be. High rates of SARS-CoV-2 transmission increase the likelihood of rare transmission events in which low-frequency variants are transmitted. Decreasing the number of infections globally through vaccination and non-pharmaceutical interventions are critical for reducing the opportunities for rare evolutionary events to occur. Ongoing assessment of SARS-CoV-2 evolution within and between individuals, especially in the face of growing immunity, will inform our understanding of how novel variants arise and sweep to fixation in the community and beyond.

Our findings are also consistent with, though do not prove, a regime in which most acute infections play a limited role in the generation and spread of new SARS-CoV-2 variants. Prolonged infections permit additional cycles of viral replication, lead to greater accumulation of intrahost mutations [[4–8]](https://paperpile.com/c/gUWjkV/fVYui+8vAnJ+iMYqA+kJfme+DDHD7), and allow more time for within-host selection. Even a modest increase in frequency enhances the likelihood of a beneficial variant being transmitted onward, and election during transmission may further propagate beneficial variants between hosts [[25]](https://paperpile.com/c/gUWjkV/QIxzs). It is therefore possible that the rare instances of prolonged infections play an outsize role in novel variant emergence. Future studies that characterize how variants arise, evolve, and transmit during persistent infections will be important for teasing apart the degree with which acute and prolonged infections contribute to global SARS-CoV-2 evolution.”

6. *The study is technically well done and the controls to identify iSNV are robust. I think the discussion is lacking focus on some of the study limitation like not knowing the viral input, the small sample size and not having epidemiological evidence on transmission pairs.*

To address comments from both reviewers, we have rewritten large portions of the discussion section and attempted to make it more focused. We thank the reviewer for this comment, and feel that the new discussion is much improved. We hope you agree.

7. *For instance, are infections with very high viral loads likely to harbor more diversity. The authors suggest that this is not the case, but this seems to be counterintuitive.*

The reviewer is correct that this is not intuitive. However, our finding agrees with those of 2 previously published studies that examined the number of variants in relation to viral copies. In Valesano et al, the authors explicitly compare the number of iSNVs to the viral copy numbers and find no relationship (Supplemental Figure 3). In Lythgoe et al, the authors compare the mean number of iSNVs to read depth as a correlate of viral input copies and similarly find no relationship (Figure 1B). Although this is not initially intuitive, our primary concern with this analysis was to ensure that low copy number samples were not enriched with spurious iSNVs. Our finding of limited impact of copy number of the number of iSNVs suggests that our protocols and examination of only intersection iSNVs effectively controls for this effect. We have clarified this intention added these references into the results in **lines 162-168**, which now read:

“Samples with low viral RNA copy numbers can sometimes result in an excess of spuriously detected iSNVs [[22]](https://paperpile.com/c/gUWjkV/b7Lc8), so we wanted to ensure that we did not detect a greater number of iSNVs in samples in our dataset with low RNA concentrations. Using input data from two different clinical assay platforms, we find no correlation between viral input copies and the number of intersection iSNVs detected, consistent with findings reported by others, and supporting the robustness of our iSNV calls to RNA copy numbers [[21,32]](https://paperpile.com/c/gUWjkV/qUxkK+IoQXL) (**Supplemental Figure 3d** and **Supplemental Figure 3e**).”

8. *As the samples were from public health surveillance, they are taken at a single time point in the infection. We still don’t know with this dataset if diversity changes over time in an individual and if this will impact on the capacity for non-dominant variants to be transmitted.*

We agree that this is an important caveat, and have added this to the discussion on **lines 475-478**:

“Finally, while we did not have access to longitudinal samples in this study, characterization of viral populations over time in individual infections will provide superior resolution regarding the dynamicity of iSNVs and their frequencies throughout the course of infection.”

9. *The limitations of the transmission pair permutation test are not discussed. How certain are we of the directionality of transmission correct?*

The permutation test does not assume directionality, and is a more coarse metric than the bottleneck estimates with the beta-binomial method. Instead, it is simply a comparison of whether iSNVs are shared between 2 samples. In contrast, the beta-binomial method explicitly assumes that one individual is the donor and the other is the recipient, and models transmission bottleneck size under this assumption. The fact that the permutation test does not assume or attempt to infer transmission direction, or compare variant frequencies are definite downsides. We now acknowledge these in **lines 323-328**:

“While the above permutation test compares the presence and absence of iSNVs between individuals, it does not assume transmission directionality or make use of variant frequencies. It is therefore a crude metric of whether samples share iSNVs, rather than a quantitative measure of transmission stringency. We therefore next inferred transmission bottleneck sizes using the beta-binomial inference method [[38]](https://paperpile.com/c/gUWjkV/2wNkn).”

10. *In general the text is long and could be made more brief and to the point.*

In the process of addressing other comments, we have attempted to shorten the manuscript and streamline text whenever possible. In particular, we have rewritten the discussion and attempted to provide more focus. We hope these revisions improve the manuscript.
